# Mechanism of the Cu^II^-catalyzed benzylic oxygenation of (aryl)(heteroaryl)methanes with oxygen†
†Electronic supplementary information (ESI) available. See DOI: 10.1039/c5sc03530a


**DOI:** 10.1039/c5sc03530a

**Published:** 2015-09-29

**Authors:** Hans Sterckx, Johan De Houwer, Carl Mensch, Ignacio Caretti, Kourosch Abbaspour Tehrani, Wouter A. Herrebout, Sabine Van Doorslaer, Bert U. W. Maes

**Affiliations:** a Department of Chemistry , University of Antwerp , Groenenborgerlaan 171 , B-2020 Antwerp , Belgium . Email: bert.maes@uantwerpen.be; b Department of Physics , University of Antwerp , Universiteitsplein 1 , B-2610 Antwerp , Belgium

## Abstract

A mechanistic study of the copper-catalyzed oxidation of the methylene group of aryl(di)azinylmethanes was performed. Initial reaction rates were measured making use of *in situ* IR reaction monitoring and a kinetic analysis of the reaction was executed. The reaction proved to be first order in oxygen concentration. For substrate and acid concentration, saturation kinetics due to O_2_ mass transfer limitation were observed. The occurrence of mass transfer limitation was further confirmed by examining the effect of the stirring rate on the initial reaction rate. Interestingly, the effect of the concentration of the catalyst on the rate shows that higher loadings result in a maximal initial rate, followed initially by a steady decrease and subsequently a rate plateau when the concentration is increased further. Mass transfer limitation and increased concentration of dinuclear catalytically active species rationalizes this hitherto unprecedented rate behavior. Continuous-wave and pulsed electron paramagnetic resonance methods were used to characterize the catalytic species present in the solution during the reaction and confirmed the presence of both mono- and dinuclear copper species. Analysis of a diverse substrate scope points towards imine–enamine tautomerization as a crucial process in the oxidation reaction. DFT calculations of these equilibrium constants (p*K*_eq_) provided us with a qualitative tool to predict whether or not a substrate is viable for oxidation under the reaction conditions developed.

## Abstract 2

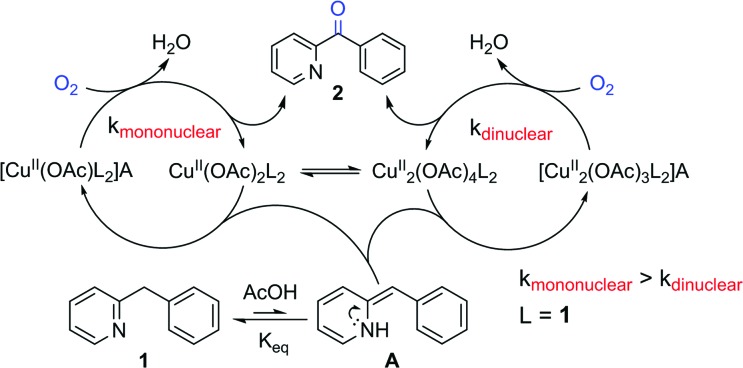
A mechanistic study of the copper-catalyzed oxidation of the methylene group of aryl(di)azinylmethanes was performed.

## Introduction

Two decades ago benzylic oxidation reactions made use of unsustainable oxidants such as potassium permanganate or chromium acid derivatives. This produced vast quantities of hazardous waste.^1^ While Mn^II^ and Cr^III^ are present as trace elements in the human body as essential nutrients, Cr^VI^ is a known carcinogen and exposure to Mn^VII^ can lead to ‘manganism’, a central nervous system disorder.^2^ Nowadays these classical oxidants are mostly shunned and a major shift has taken place towards less toxic and sustainable oxidants like peroxides or molecular oxygen preferably in combination with a cheap transition-metal catalyst.^3^ Recently substantial progress has been made in the development of preparative base metal-catalyzed aerobic oxidation reactions of benzylic alcohols.^4^ The direct benzylic methylene oxygenation is however far less studied.^5^ Notable examples using peroxides as the terminal oxidant on active methylenes are reported by the groups of Bolm, Punniyamurthy, Chen and Sasson using respectively FeCl_3_, a Cu^II^ Salen-H_4_, a Mn(OAc)_2_·4H_2_O complex and CuCl_2_ as the catalyst.^6^,^7^ Methylene oxidations using copper and iron with molecular oxygen as the terminal oxidant are of special interest. After all, these metals are abundant, feature a low toxicity, and oxygen is the most sustainable oxidant on earth.^8^ There is a great number of important articles available on the structure and reactivity of various Cu- and, to a lesser degree, Fe-complexes with molecular oxygen.^9^ The stoichiometric study of these complexes provides a simplified mimic of the active site of several important metalloproteins (for instance: tyrosinase, dopamine β-monooxygenase, galactose oxidase or cytochrome P450) and are therefore easier to study. These complexes are usually very well defined and are formed by reaction of a reduced metal center (Cu^I^ and Fe^II^) with O_2_ to form the reactive intermediate, which reacts directly with the substrate. Alternatively, they can also be formed by using metals in a higher oxidation state (Cu^II^ and Fe^III^) in combination with H_2_O_2_. A stabilizing nitrogen-containing ligand (bidentate or higher) is a prerequisite for these systems to be stable enough to be studied. Furthermore the ligand provides a mimic of the polypeptide chain around the metal center and can be used to tune the reactivity of the complex.

**Scheme 1:**
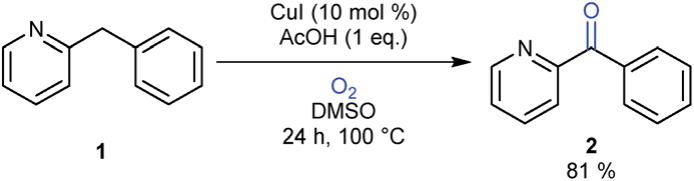
Oxidation of model substrate 2-benzylpyridine (**1**) to 2-benzoylpyridine (**2**) under the standard reaction conditions.

In comparison to the reports describing well characterized model complexes in stoichiometric reactions, preparative protocols for oxygenation of active methylenes involving *in situ* formed copper and iron catalysts with oxygen as the stoichiometric oxidant are still limited.^10^,^11^ In 2012 our group published a communication describing a synthetic protocol for the copper- and iron-catalyzed oxidation of the methylene group of aryl(di)azinylmethanes with acetic acid as promoter (Scheme 1).11*a*

Several groups have made creative use of these reaction conditions and some extended them to other substrate systems. Kappe successfully translated our protocol into an efficient flow process allowing scale up and continuous production.^12^ By slightly modifying our disclosed reaction conditions Touré achieved selective oxidation of C–H bonds in drug candidates and APIs delivering drug metabolites. The oxidation protocol could also be applied to oxygenate carbons alpha to a tertiary amine and therefore mimic or complement cytochrome P450.^13^ Very recently Zhuo and Lei disclosed a similar oxidation protocol in which ethyl chloroacetate was used as a promoter instead of acetic acid.^14^ A final interesting and notable example of an extension towards other functionalizations is the copper-catalyzed α-methylenation of benzylpyridines using DMA as a one-carbon source.^15^ When Na_2_S_2_O_8_ was used as an oxidant instead of O_2_, chemoselective methylation occurred over oxygenation of the methylene. Although there are several publications describing preparative methylene oxidation protocols based on copper or iron and O_2_, no detailed mechanistic studies (involving kinetics) have been performed yet to support the proposed catalytic cycles. As copper and iron salts easily form polynuclear species, the nature of the catalyst in these reactions remains also unknown. To allow further developments in this field, knowledge of the catalytically active species and the catalytic cycle is crucial. In this paper we disclose the results of mechanistic studies on the oxidation of 2-benzylpyridine under copper catalysis with oxygen as oxidant as model system (Scheme 1), leading to an insight into the actual reaction mechanism of this intriguing transformation. DMSO was chosen as the solvent for this mechanistic study to avoid solubility problems considering the large ranges of concentrations of the different reaction components that had to be used.

## Results and discussion

### Substrate scope: structural requirements

The previously developed reaction conditions (Scheme 1) allowed to oxidize 2- (**1**) and 4-benzylpyridine (**6**), while no oxidation of 3-benzylpyridine (**4**) and diphenylmethane (**8**) occurred.11*a* From this was concluded that the oxidation is not a directed reaction since coordination of the metal catalyst to the nitrogen in 4-benzylpyridine would bring the metal too far away from the benzylic position to promote an intramolecular reaction. Instead, these observations point to an imine–enamine tautomerization as a prerequisite for the reaction to proceed. Besides, the acidity of the methylene protons is also important since 2-methylpyridine (**10**) could not be oxidized. Further support that a combination of these two factors is necessary was gathered by performing experiments on a selected set of substrates (Table 1). When the acidity of the methylene in **10** is increased by introducing an electron-withdrawing group, either on the pyridine ring or on the methylene group, the substrate can be oxidized again as exemplified by methyl 2-(pyridin-2-yl)acetate (**14**) and 6-methylnicotinonitrile (**12**). A control experiment with methyl 2-phenylacetate (**16**) proves that the imine moiety is still necessary and that activation by the methyl ester alone is not sufficient for this reaction to proceed. To test whether this oxidation protocol can also be used beyond azine type systems it was applied to azoles; 2-benzyl-1-methyl-1*H*-imidazole (**18**) and 1,2-dimethylimidazole (**20**). The phenyl-activating group appears to still be a necessary structural element since we were only able to oxygenate the former substrate, similarly to what has been observed for **10**. Other 2-benzylazoles can also be used, as shown by the oxidation of 2-benzylbenzoxazole (**22**) and 2-benzylbenzothiazole (**24**), further underlining the general applicability of the oxidation method.

**Table 1:** Calculated p*K*_eq_ values and Cu-catalyzed oxidation reaction for structurally different substrates

Entry	Substrate	Product	p*K*_eq_	Yield^*a*^ (%)
1	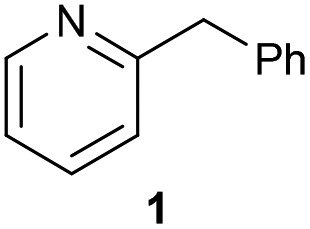	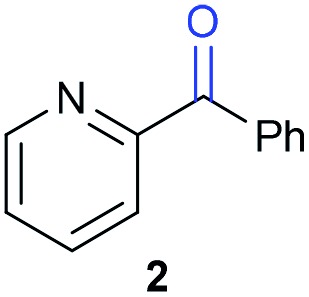	11.5	92^*d*^
2	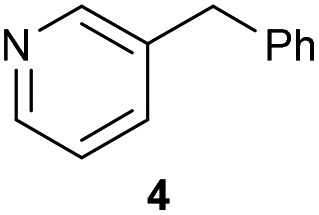	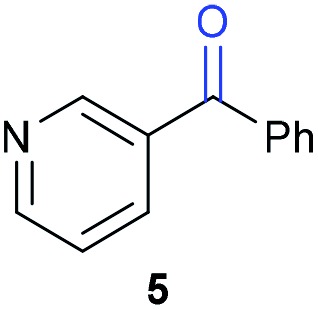	20.4	0^*d*^
3	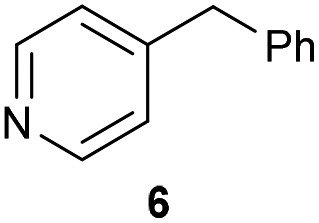	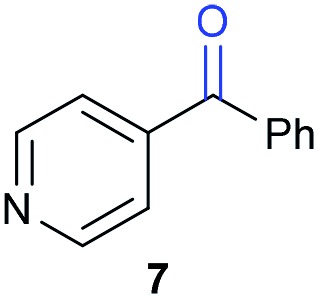	11.0	89^*d*^
4	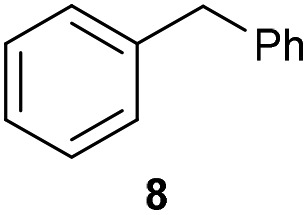	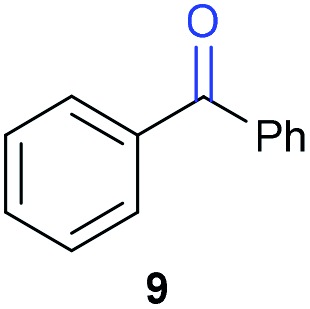	21.7	0^*d*^
5	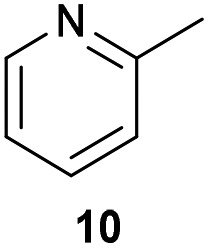	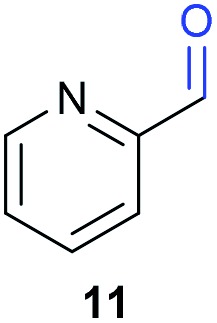	15.9	0^*d*^
6	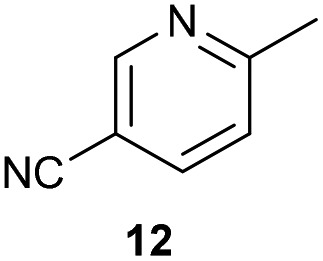	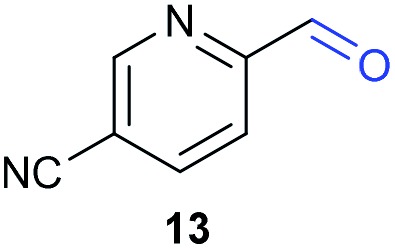	13.0	27 (60)^*b*^
7	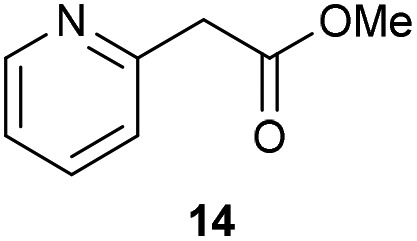	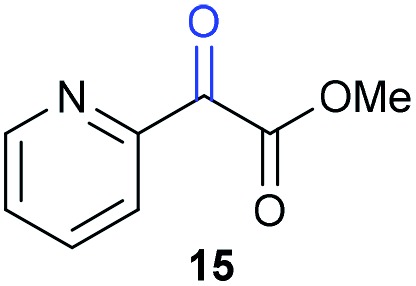	6.2	23 (83)^*c*^
8	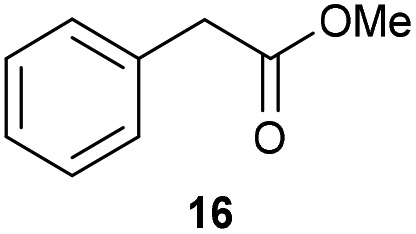	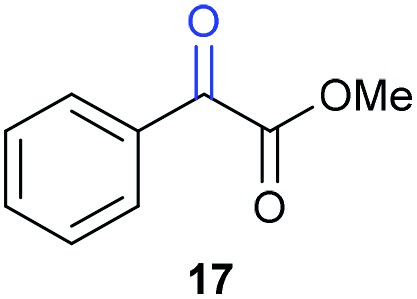	18.8	0
9	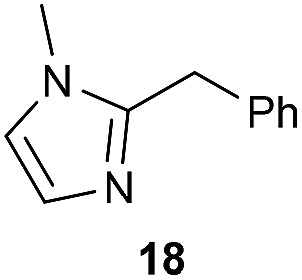	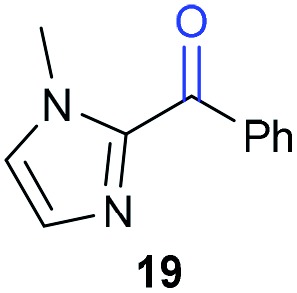	13.9	34 (85)^*b*^
10	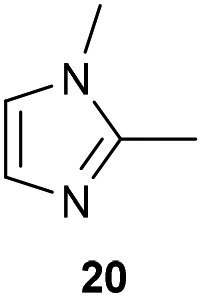	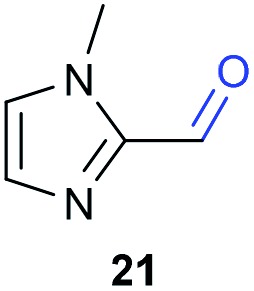	19.0	0
11	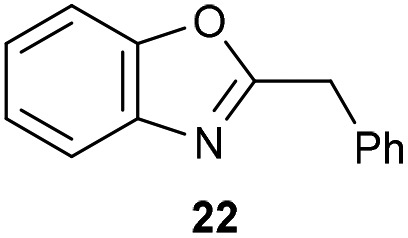	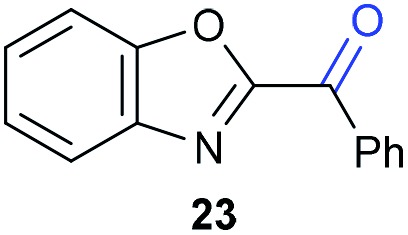	9.1	98
12	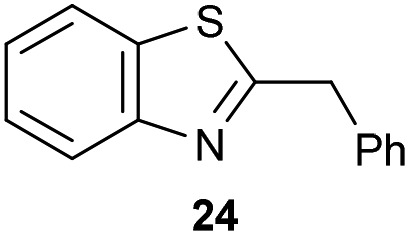	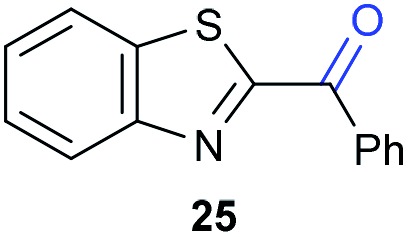	8.1	99

^*a*^Substrate (0.5 mmol), CuI (10 mol%) AcOH (1 eq.), DMSO (1 mL), O_2_ (balloon), 100 °C, 24 h.

^*b*^At 120 °C.

^*c*^In *n*-BuOAc (1 mL) with addition of molecular sieves.

^*d*^Compound reported in our communication, see ref. 11*a*.

### Imine–enamine tautomerization: computational study

**Fig. 1:**
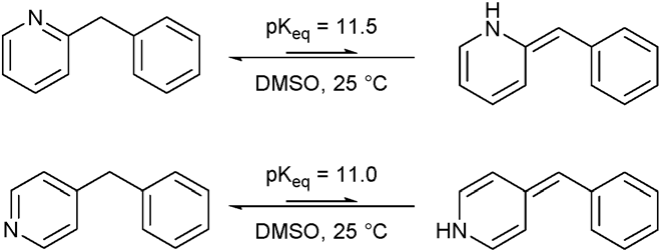
Imine–enamine equilibrium of 2- and 4-benzylpyridine.

The structural features of substrates suitable for oxidation under our protocol (*vide supra*) indicated that the mechanism of the oxidation seems to revolve around an (initial) imine–enamine tautomerization. The imine–enamine tautomerization can be thermodynamically quantified by an equilibrium constant *K*_eq_ (Fig. 1). To gain more insight in these tautomerizations, the p*K*_eq_ values for the different experimentally tested substrates were calculated using density functional theory (DFT, Table 1).

First, the geometries of the different conformations of both the imine- and the enamine-form were optimized at the B3PW91/aug-cc-pVDZ level of theory. Due to the rather large dipole moment of the molecules and the difference in dipole moment of the imine and the respective enamine tautomer (Table S5†), the self-consistent reaction field (scrf) model was used to account for solvent–solute interactions with DMSO. In a second phase, the most stable imine and respective enamine conformer were used to calculate the Gibbs free energy (Δ*G*°) and the desired equilibrium constant *K*_eq_ (see ESI† for more details). All calculations were performed using the Gaussian09 program. In Table 1 the results of these calculations for the different experimentally tested benzylic systems are summarized. These calculated values do not have a direct absolute meaning but can be compared relatively between different substrates.

Benzylpyridines (**1** and **6**) have a p*K*_eq_ value of 11.5 and 11.0, respectively, which is up to 9 orders of magnitude lower than 3-benzylpyridine (**4**), with a p*K*_eq_ value of 20.4. This is because in 3-benzylpyridine stabilization by an enamine tautomeric form is not possible, as is also the case for diphenylmethane (**8**). Furthermore, without the second aromatic ring attached to the methylene center the p*K*_eq_ rises with about 4 orders of magnitude, as exemplified by 2-methylpyridine (**10**). Introducing an electron withdrawing group either on the pyridine or on the methylene moiety results in a higher equilibrium constant (**12** and **14**). Although a keto–enol tautomerization can be drawn for the methylester functionality of methyl 2-phenylacetate (**16**) it still provides a relatively high p*K*_eq_ value (18.8) (see Table S4† for more details on the tautomerization equilibria).

Interestingly, from Table 1, a clear correlation exists between the calculated p*K*_eq_ values and whether oxidation occurs or not for each substrate. Only the substrates with a sufficiently low p*K*_eq_ or high *K*_eq_ value are reactive enough to be oxidized. Empirically, based on Table 1 and the p*K*_eq_ values calculated for substrates previously published, one can deduce the maximum p*K*_eq_ value for the reaction to proceed under our standard reaction conditions to be around 15.

Homolytic benzylic C–H bond dissociation energies (BDE) of the molecules in Table 1 were also calculated (see Table S6†). If a correlation exists this would be an indication for a mechanism in which the active catalyst abstracts a hydrogen atom from the substrate before quenching with O_2_. However, no correlation could be found since the bond dissociation energies of diphenylmethane (**8**), 2-, 3-, and 4-benzylpyridine (**1**, **4**, and **6**) are similar while there is a major difference in their reactivity in our oxidation protocol. DFT calculations thus provide an interesting qualitative tool to predict whether or not a substrate will be viable for oxygenation under our reaction conditions and strongly suggest that the oxidation reaction proceeds *via* the enamine tautomer as the reactive species.

### Kinetic experiments

**Fig. 2:**
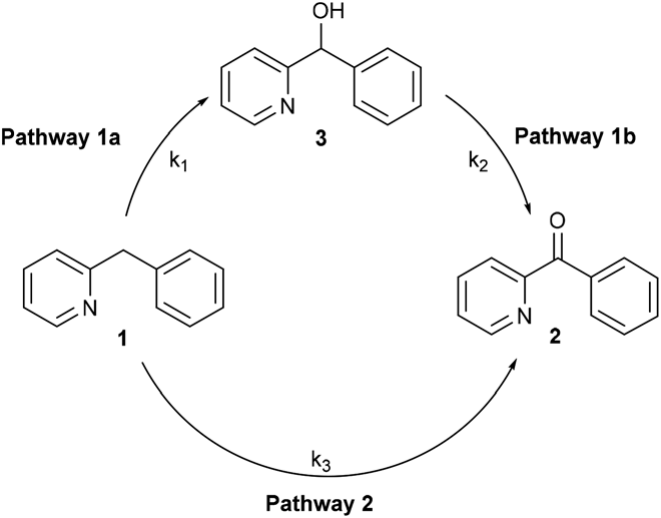
Two possible reaction pathways in the oxidation of **1**.

In the oxidation reaction of **1**, small amounts of phenyl(pyridin-2-yl)methanol (**3**) were observed besides the desired 2-benzoylpyridine (**2**).11*a* This raised the question whether formation of the 2-benzoylpyridine (**2**) proceeds *via* phenyl(pyridin-2-yl)methanol (**3**) as an intermediate (Fig. 2, pathway 1a/1b) or *via* a pathway directly from 2-benzylpyridine (**1**) (Fig. 2, pathway 2), in which case the alcohol is merely a formed side product.

**Scheme 2:**
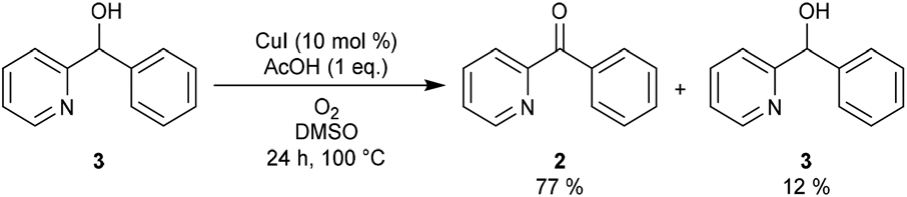
Copper-catalyzed oxidation reaction of **3** under standard conditions.

As β-hydride elimination of copper alkoxide complexes is known but rare, we first tested whether alcohol **3** could be oxidized under our reaction conditions.^16^ When the optimal reaction conditions were applied (Scheme 2), ketone **2** was isolated in 77% yield with 12% recovery of **3**.

**Fig. 3:**
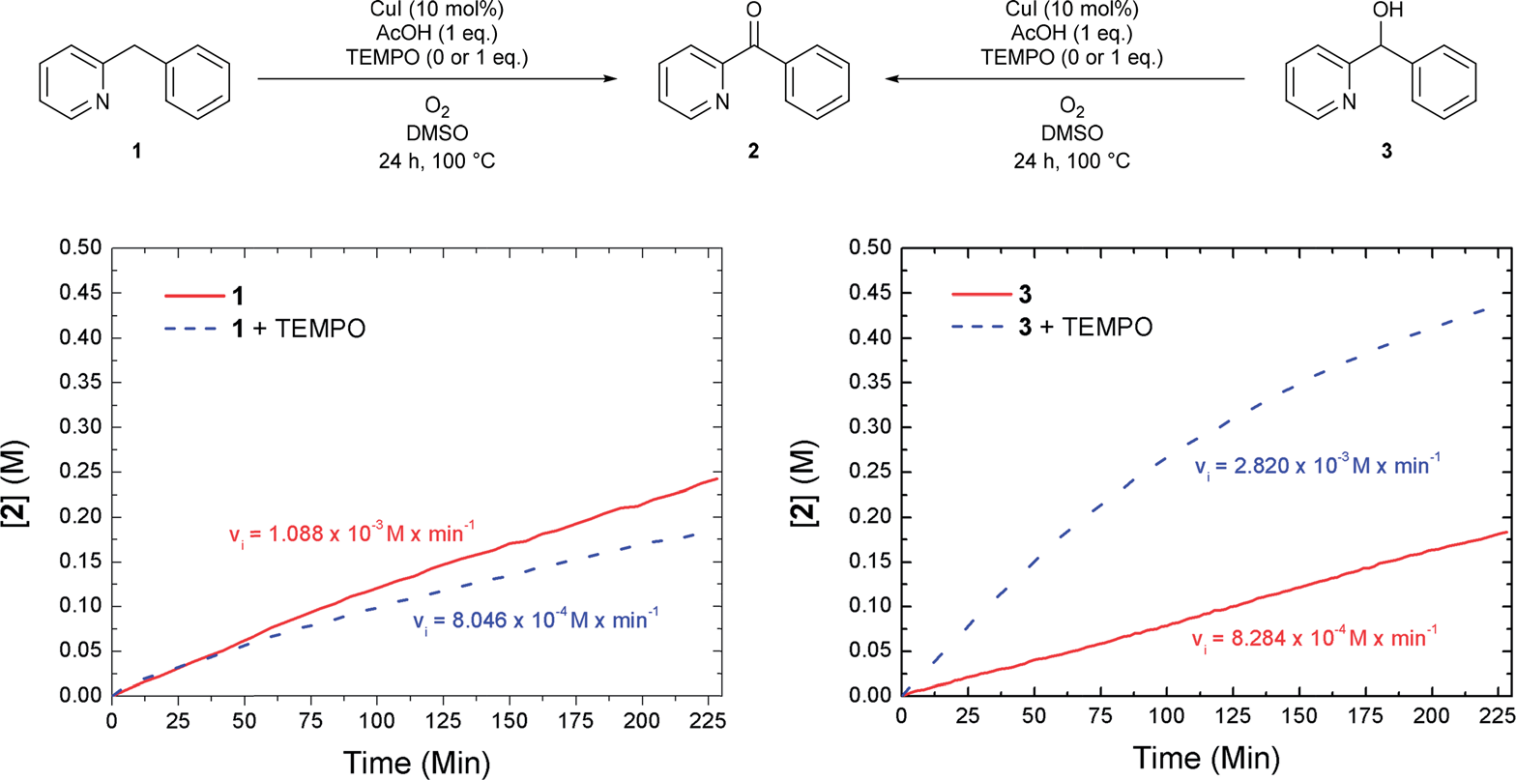
Effect of TEMPO on the rate of the oxidation reaction of **1** and **3**.

To gain more insight we performed a kinetic study using *in situ* IR monitoring. The concentration of ketone **2** was monitored over time (CO stretch frequency) and the initial reaction rate was determined. Both the oxidation of **1** and **3** (Fig. 3) were followed in function of time. For each of these transformations the results were compared to a similar reaction where 1 equivalent of the radical scavenger 2,2,6,6-tetramethylpiperidinyloxyl (TEMPO) was added to the reaction mixture. Fig. 3 (left) shows that the presence of TEMPO has almost no influence on the formation of ketone **2**, starting from **1**. However, the oxidation of alcohol **3** is significantly accelerated in the presence of the radical scavenger (Fig. 3, right). Stahl already reported that smooth oxidation of alcohols can be achieved by the use of a radical catalyst, such as TEMPO, in combination with a copper catalyst and oxygen as the stoichiometric oxidant, which accounts for these observations.^17^ Assuming that the reaction proceeds exclusively *via* pathway 1a/1b the first step (1a) should be rate determining to account for a benzylic oxidation in **1** to ketone **2** which is not influenced by a radical scavenger, while the oxidation of alcohol **3** (pathway 1b) to the same product is significantly accelerated by its addition. Contradictorily, a comparison between the initial rate of oxidation of **1** and **3**, shows that the oxidation of the latter is in fact slower than the oxidation of the former. On the other hand, if the second step (pathway 1b) would be rate determining, one expects a build-up of alcohol in the reaction mixture. This is not the case since only small amounts are present during the reaction course (*vide supra*). These contradictions imply that an indirect pathway 1a/1b can be excluded and our reaction proceeds *via* a direct pathway 2 with the formation of **3** as side compound. In addition to the kinetic experiments using TEMPO we also tried to isolate TEMPO-adducts to find out whether stable radical species were formed during the reaction.^18^ No such adducts could be observed *via* LC-MS analysis. When using 1,1-diphenylethylene as an alternative radical scavenger the oxidation reaction was also not inhibited and **1** was fully converted into ketone **2** and 1,1-diphenylethylene was quantitatively recovered.19*a* Next to these soluble radical scavengers activated carbon was used as a solid scavenger. Also in this case no inhibition could be detected (Fig. S27†).19*b* These results suggest that no stable organic radical species are formed in solution during the course of this reaction. This is also supported by EPR analysis (*vide infra*).

**Scheme 3:**
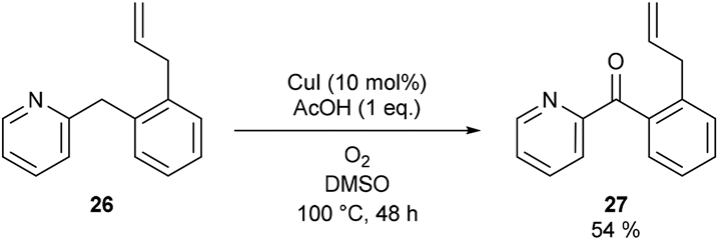
Benzylic radical clock reaction.

Considering that TEMPO, 1,1-diphenylethylene and activated carbon are bimolecular probes and oxygen is also a radical trap, we synthesized a substrate which can act as an intramolecular probe for a benzylic radical. 2-(2-Allylbenzyl)pyridine (**26**) was selected for this purpose. While benzylic radical clocks are known to be relatively slow due to the inherent stability of the benzylic radical, the reported unimolecular rate constant (*k* = 3 × 10^2^ s^–1^) is still a factor of 10^7^ faster than our overall maximum reaction rate (2 × 10^–5^ M s^–1^) (*vide infra*).19*c* (2-Allylphenyl)(pyridin-2-yl)methanone (**27**) was formed and no ring closed products due to radical cyclization could be detected in the reaction mixture (Scheme 3). Formation of **27** further supports that a free benzylic radical is not involved in our reaction protocol.

**Fig. 4:**
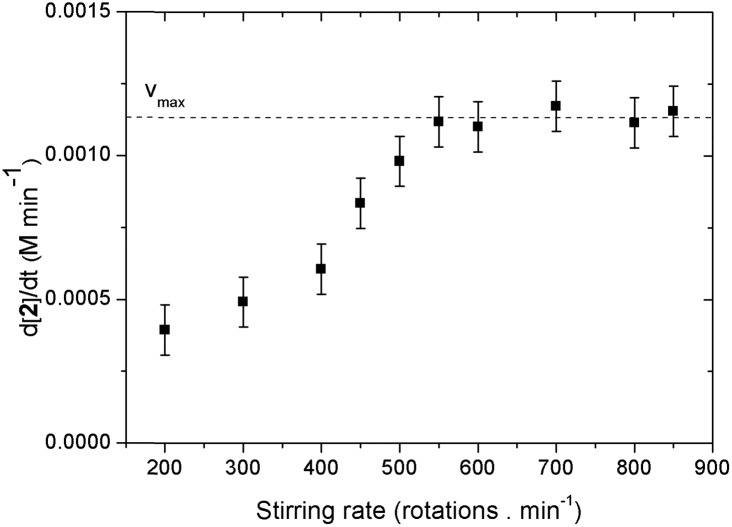
Effect of the stirring rate on the initial reaction rate.

It is important to determine whether mass-transfer effects are in play since O_2_ is a gaseous reagent.^20^ The effect of the stirring on the initial reaction rate was examined at 0.2 M of catalyst (Fig. 4). It was found that up until 550 rpm there is a first order relation of the stirring rate on the initial rate of the reaction. At higher stirring rates the *v*_i_ becomes independent. From this we can conclude that at a certain reaction rate (*v*_max_) the uptake of O_2_ becomes rate limiting. From the stirring rate experiment we see that the value of *v*_max_ is approximately 1.2 mM min^–1^. Under our standard reaction conditions we work at a stirring rate of 700 rpm and thus no mass-transfer effects are in play.

**Fig. 5:**
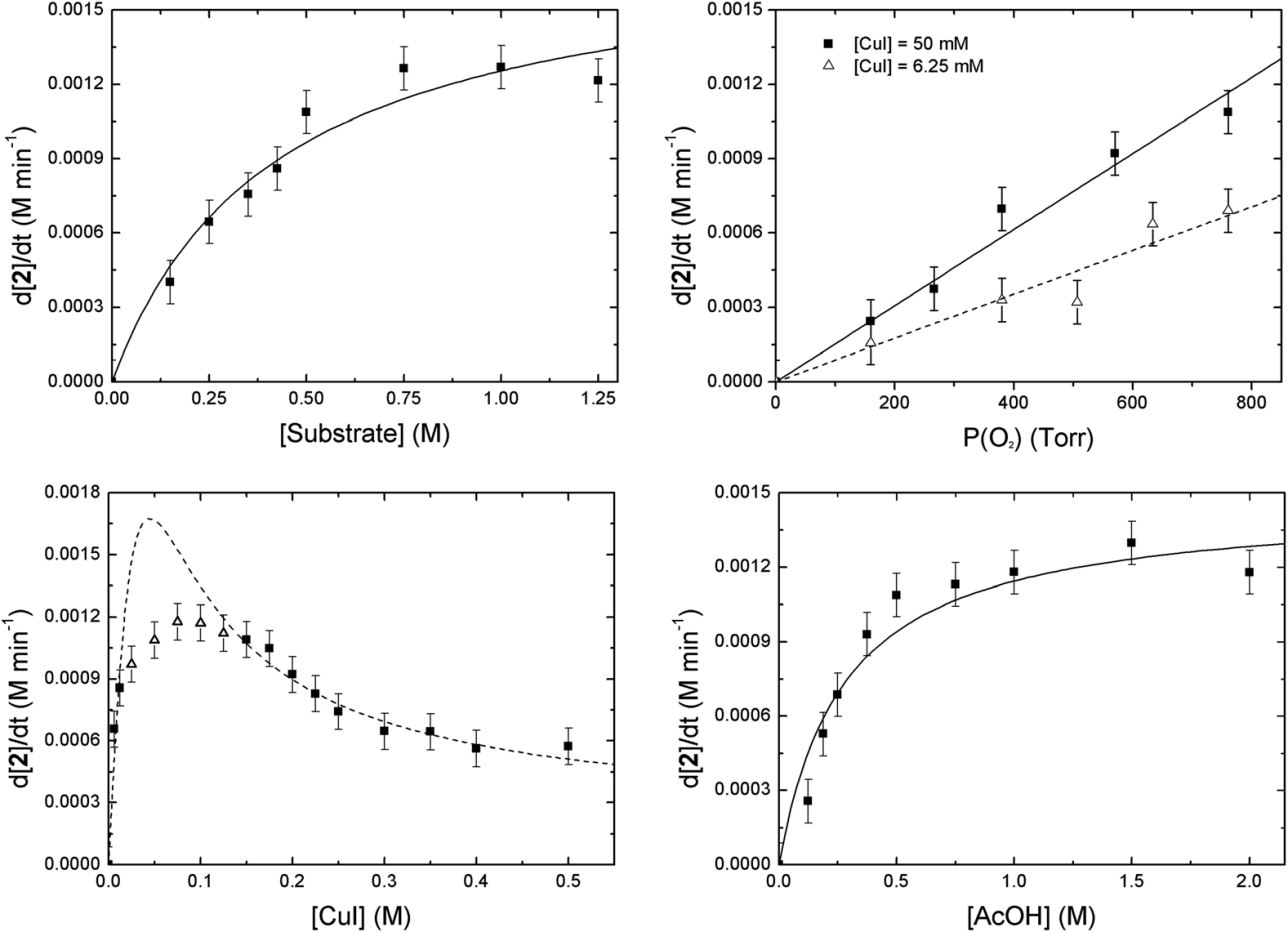
Dependence of the copper-catalyzed oxidation of 2-benzylpyridine (**1**) on [**1**] (upper left), O_2_ partial pressure (upper right), [Cu] (lower left) and [AcOH] (lower right) on the initial reaction rate. The dashed curve for the [CuI] (lower left) is a fit of the following equation: d[**2**]/d*t* = (*c*_1_[Cu]^2^ + *c*_2_[Cu])/(*c*_3_[Cu]^2^ + *c*_4_[Cu] + *c*_5_) to the datapoints (■). The data points for the [AcOH] and [**1**] respectively fit to the following equations: d[**2**]/d*t* = *c*_1_[AcOH]/(*c*_2_[AcOH] + *c*_3_) and d[**2**]/d*t* = *c*_1_[**1**]/(*c*_2_[**1**] + *c*_3_). Reaction conditions: **1** (5 mmol), CuI (10 mol%), AcOH (1 eq.), O_2_ (balloon), DMSO (10 mL), 100 °C.

A first order dependence was found for the O_2_ partial pressure (Fig. 5, upper right, solid line). Since this first order relation might also derive from the observed mass transfer limitation effects we also examined the influence of the O_2_ partial pressure at a very low catalyst concentration (6.25 mM *versus* 50 mM) to make sure that no mass transfer limitation is in play. In this instance again a first order relation was found (dashed line). From this we can conclude that the observed first order relation derives directly from the involvement of O_2_ in or before the rate determining step.

For the substrate (**1**) as well as for the acetic acid concentration saturation kinetics are seen. A quasi linear (first order) dependence is observed when less than 1 equivalent (0.5 M) of acetic acid or **1** is used (Fig. 5, upper left and lower right). When higher concentrations are used the reaction approaches zeroth order for the acid and **1** concentrations. This saturation behavior with *v*_max_ at approximately 1.2 mM min^–1^ is due to the occurrence of O_2_ mass transfer limitation conditions as was the case for the stirring experiments (*vide supra*).^20^ The measured data points are in accordance with hyperbolic functions (see ESI† for derivations). An alternative explanation for the saturation behavior might be the formation of a pre-equilibrium between substrate, AcOH and catalyst before rate determining oxygenation.17*b* This scenario, however, can be excluded. After all, when the concentration of these three components was raised simultaneously, thereby keeping their respective ratios constant, the initial rate again showed the same *v*_max_ value (Fig. S16†).

The dependence of the reaction rate on the catalyst concentration follows a more complex pattern. At very low catalyst concentrations the rate increases very fast in a hyperbolic fashion. At a rate of 1.2 mM min^–1^ it also reaches a maximum and after that the reaction rate becomes invariant of the catalyst concentration. The value of this maximum is determined by the O_2_ mass transfer limitation and corresponds to the *v*_max_ that was observed for [AcOH] and [**1**]. However, when the catalyst loading is further increased, the reaction rate diminishes significantly (Fig. 5, lower left) to finally settle at a constant rate independent of the catalyst concentration. As the occurrence of mass transfer limitation would result in a hyperbolic curve approaching 1.2 mM min^–1^ as *v*_max_, the significant drop in initial rate cannot be explained by the occurrence of mass transfer limitation and another factor has to be taken into account. Other examples of non-linear dependence on [catalyst] for aerobic oxidations have previously been reported by the groups of Stahl and Sheldon.17b,^21^ We attribute the continuous drop in rate to the existence of dinuclear Cu-species. Trapping the active catalyst in a dinuclear species can inhibit the reaction since adding more catalyst will only result in the formation of more dimer *via* a second order process in [catalyst]. The non-zero reaction rate at very high catalyst loadings can only be explained by considering that these dinuclear species are also catalytically active (rate constant *k*_d_), however substantially less reactive than the mononuclear species (rate constant *k*_m_) (*k*_m_ > *k*_d_). This is interesting as usually non-reactive dinuclear complexes are considered. A rate law (eqn (1)) was derived (see ESI†) which takes into account reactive di-nuclear complexes but no mass transfer limitation.^22^ A very good fit could be obtained for the data points before and after mass transfer limitation (dashed line). Between a copper concentration from 25 mM to 125 mM (triangles) where O_2_ diffusion is at play the simulated rate is much higher than the measured data points as expected.
1

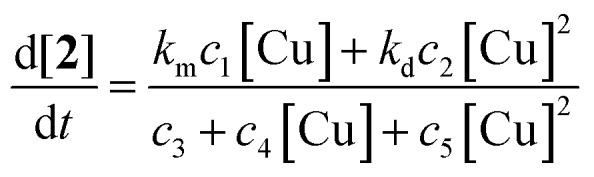




**Scheme 4:**
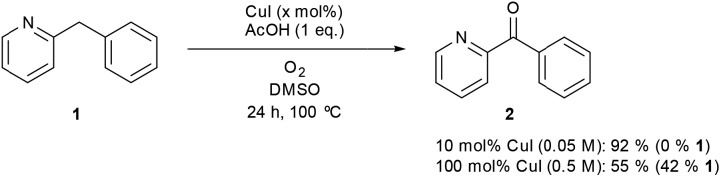
Control experiments with (super) stoichiometric amounts of catalyst.

To support the catalytic activity at very high catalyst loading an experiment was performed with a stoichiometric catalyst loading. Significant conversion and a moderate yield was seen after 24 hours (Scheme 4) which is in accordance with the kinetic measurements. No significant side reactions take place at high catalyst loading as the reaction gave a good mass balance (>90%).

To exclude the possibility of any catalyst degradation the reaction was monitored with *in situ* IR and, after the reaction was complete, another batch of substrate (**1**) was added. The second reaction proceeded with no significant loss in reaction rate (see Fig. S21†). Work-up of this reaction mixture provided ketone **2** in 92% yield with no starting material remaining. From this we conclude that no significant degradation of the catalyst to inactive (dimeric) species is occurring.

### Structure of the catalytic species: an EPR-study

In order to identify the copper species present during the reaction, continuous-wave (CW) and pulsed EPR experiments were performed on the oxidation reaction of the model substrate **1** shown in Scheme 1.^23^ In the initial screening of catalysts it was shown that the reaction worked well for most copper sources, irrespective of the oxidation state (Cu^0^, Cu^I^, Cu^II^).11*a* As we are working under O_2_ atmosphere in the presence of acetic acid, the active catalytic species is likely to contain a Cu^II^ center and the reaction therefore involves initial oxidation when salts at lower oxidation state are used as precatalyst (eqn (2)).^24^
2

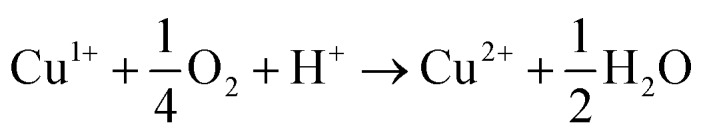




The oxidation of Cu^I^ in the presence of pyridine to hydroxide-bridged polynuclear complexes has been well-documented.^25^ The situation in our case is different: since we are able to start from a Cu^II^ source. This would require the use of H_2_O_2_ in stead of O_2_ to end up with the same reactive species. Possible anions for the Cu^II^ species are iodide, acetate and hydroxide. The latter is a result from the oxidation of Cu^I^ while the former two are deliberately added to the reaction. Since no ligand is added it is likely that DMSO will ligate (S- or O-ligation) to the copper center to stabilize it in solution.^26^ Alternatively the substrate (**1**) or reaction product (**2**) can also act as a ligand. The intermediate reacting with O_2_ likely contains a Cu^II^–C(sp^3^) bond with the methylene carbon (Fig. 10, **B** and **B′**). As stated above this bond can be formed through initial tautomerization of substrate followed by reaction with copper species. However, since the equilibrium of this tautomerization is highly shifted towards the imine tautomer we expect the concentration of this intermediate to be very low during the course of the reaction.

Firstly, control CW-EPR measurements at 100 K were performed on a solution of 0.05 M CuI in DMSO heated to 100 °C during 10 minutes under O_2_ atmosphere (Fig. S1(a)†). The Cu^II^ complex observed (species I) (Table 2, entry 1) has *g* and copper hyperfine values that are similar to those reported earlier for CuBr_2_·2H_2_O in DMSO and is in accordance with the complexation of Cu^II^ with DMSO ligands.^27^ The same experiment was performed, but now in the presence of 0.5 M AcOH (Fig. S1(b)†); copper(ii) acetate is known to exist mainly in a dinuclear form Cu_2_(OAc)_4_ in acetic acid and other solvents, with only a small amount of mononuclear Cu(OAc)_2_ complex present.^28^ Both types of Cu^II^ species (monomeric and multimeric) can be distinguished by EPR. Indeed, two Cu^II^ mononuclear complexes (species I′ and II) are detected (Table 2, entry 2, Fig. S1(b)†), together with high-field signals from Cu^II^ dimers (Fig. S2†). Species I′ has parameters similar to those of the complex observed without acetic acid and can be assigned to the same complex. The small shifts in the parameters are induced by the change in the dielectric constant of the mixture. Species II features EPR parameters similar to those observed earlier for Cu(OAc)_2_·2H_2_O in different solvents.^29^ With regard to the dinuclear species, the strong antiferromagnetic coupling within the pair of copper ions in Cu_2_(OAc)_4_ generally results in a triplet state that lies approximately 250–300 cm^–1^ (=|*J*|) above the singlet state, with typical zero-field splitting (*D*) for the triplet of the order of 0.34 cm^–1^ and characteristic EPR spectra.^30^ Fig. S2† shows the temperature dependence of one of the high-field signals of this contribution, from which the exchange coupling *J* can be determined (*J* = –274 cm^–1^), in agreement with what was earlier reported for Cu_2_(OAc)_4_(H_2_O)_2_, namely *J* = –269 cm^–1^ in frozen acid solution.^31^ In the EPR spectra of the mixture without addition of acetic acid (Table 2, entry 1), no such dinuclear copper complex could be found, in accordance with the absence of bridging ligands.

**Table 2:** Principal *g* and copper hyperfine values of the mononuclear Cu^II^ complexes in the different reaction mixtures heated to 100 °C^*a*^

Entry		[CuI] (M)	Heating time		*g*_*x*_ ± 0.002	*g*_*y*_ ± 0.002	*g*_*z*_ ± 0.001	|*A*_*x*_| (MHz) ± 15	|*A*_*y*_| (MHz) ± 15	|*A*_*z*_| (MHz) ± 5
1	CuI/DMSO	0.05	10′	I	2.082	2.087	2.405	45	55	385
2	CuI/DMSO/AcOH	0.05	10′	I′	2.081	2.083	2.403	26	25	375
				II	2.080	2.080	2.374	30	26	430
3	CuI/DMSO/AcOH/(**1**)	0.05	5′	III^*b*^	2.070	2.070	2.333	25	25	440
				IV	2.062	2.063	2.286	35	35	510
4	CuI/DMSO/AcOH/(**1**)	0.05	10′	III^*b*^	2.070	2.070	2.333	25	25	440
				IV	2.062	2.063	2.286	35	35	510
5	CuI/DMSO/AcOH/(**1**)	0.05	1 h	IV	2.063	2.063	2.285	30	30	510
6	CuI/DMSO/AcOH/(**1**)	0.05	4 h	IV′	2.063	2.063	2.290	30	30	505
				V	2.067	2.067	2.262	35	35	560
7	CuI/DMSO/AcOH/(**1**)	0.25	10′	IV	2.066	2.066	2.286	35	35	510
				III^*c*^	2.072	2.072	2.330	25	25	450
8	CuI/DMSO/AcOH/(**1**)	0.25	4 h	IV	2.062	2.063	2.285	35	35	510
				III^*c*^	2.075	2.072	2.334	25	25	455

^*a*^The relative contributions of the different species to the experimental spectra are given in the ESI.

^*b*^For the simulation of the contribution of species III in reaction mixture with starting concentration [CuI] = 0.05 M, the interaction with two ^14^N nuclei with a isotropic hyperfine coupling of 40 ± 5 MHz had to be taken into account.

^*c*^The ^14^N hyperfine interaction was not resolved due to the increased linewidth, but was considered in the simulation.

**Chart 1:**
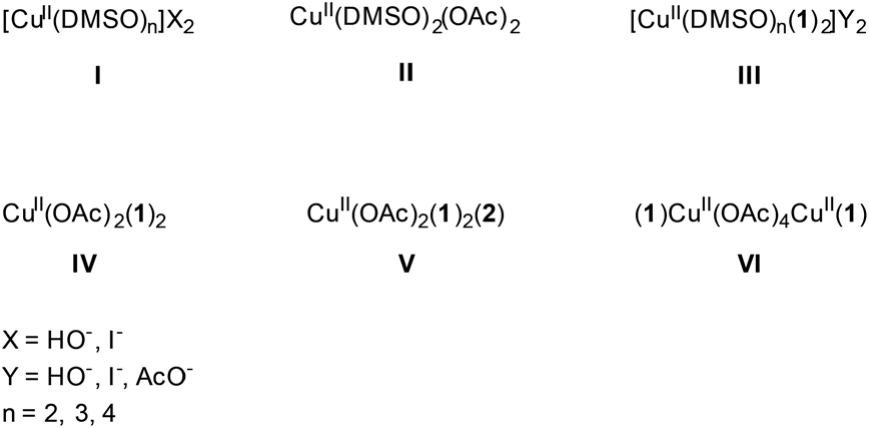
Mono- and dinuclear copper species present in the reaction.

**Fig. 6:**
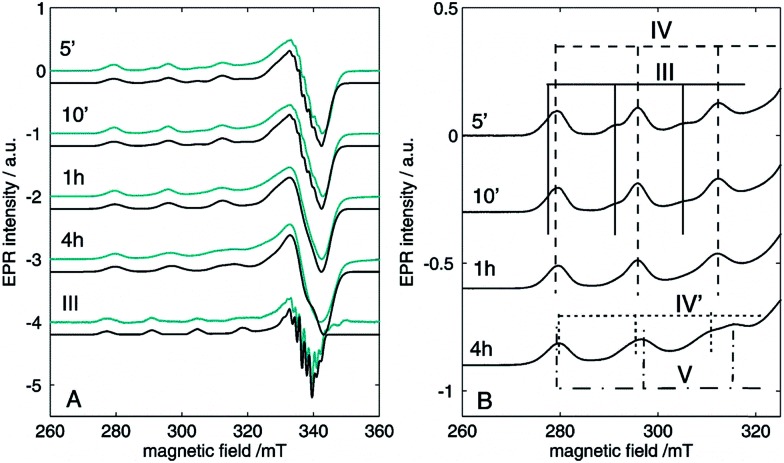
Experimental (cyan) and simulated (black) CW-EPR spectra of the reaction mixture with 0.05 M CuI and 0.5 M 2-benzylpyridine (**1**), 0.5 M acetic acid and DMSO heated to 100 °C under O_2_ for 5 min, 10 min, 1 h and 4 h. The spectra were recorded at 100 K with a microwave power of 0.47 mW and a microwave frequency of 9.73 GHz. (A) The spectrum of species III, obtained by subtraction of the spectra obtained after 5 and 10 minutes is shown at the bottom with its corresponding simulated spectrum. (B) Zoom on the low-field components revealing the time-dependent evolution of the different species. The experimental spectra are normalized for comparison.

Next, EPR measurements were done on reaction mixtures. Fig. 6A shows the EPR spectra observed after heating a reaction mixture for 5 min, 10 min, 1 h and 4 h. The field area specific for mononuclear Cu^II^ complexes is shown for the spectra recorded at 100 K. None of the EPR spectra showed signals typical of organic radicals. Instead, a clear evolution of mononuclear Cu^II^ complexes is observed in function of the reaction time, with different species contributing to the spectra, as is best visible in the low-field range (Fig. 6B). At short reaction times (5 and 10 min), the EPR spectra revealed two species, labeled III and IV, with the contribution of III decreasing over time. After 1 h, only component IV is left and as the reaction progresses, a contribution of a new species V starts to emerge. The EPR parameters of species IV are slightly changing with time (species IV′), which may be related to changes in the direct neighborhood of the complex. The EPR spectrum of species III can be obtained by a weighted subtraction of the spectra recorded after 5 and 10 minutes (bottom spectrum in Fig. 6A). In the high-field region, clearly resolved hyperfine couplings are visible that can be satisfactorily simulated considering the interaction of the unpaired electron with the copper nucleus and with two ^14^N surrounding nuclei. The hyperfine coupling of the latter interaction (40 MHz) is similar to that obtained for the hyperfine value of the pyridine nitrogen nuclei in Cu(py)_4_ complexes.^32^ Given that species III is observed at an early stage of the reaction it can be ascribed to an initial complexation of the copper ion with two 2-benzylpyridine (**1**) molecules, [Cu(DMSO)_*n*_(**1**)_2_]Y_2_ (Fig. 6), where *n* = 2, 3 or 4 and Y = ^–^OH, ^–^I or ^–^OAc. Moreover, when the same reaction is carried out in toluene instead of DMSO, the CW-EPR signal after 5 minutes reaction (Fig. S12†) shows only species with *g* and *A* parameters similar to species IV (see Table S3†), without contribution from DMSO-related species III. The EPR parameters of species IV are very close to those reported for a powder of Cu(O_2_CCPh_3_)_2_·2py and for a frozen solution of CaCu(OAc)_4_·6H_2_O in pyridine.^33^,^34^ Addition of pyridine to dinuclear copper carboxylates has been shown to yield dinuclear complexes in equilibrium with mononuclear species of the form Cu(O_2_CR)_2_py_2_.^31^ The structure of IV can therefore be assigned to Cu(OAc)_2_(**1**)_2_ (Fig. 6). This is further supported by the decrease of *g*_*z*_ and increase of |*A*_*z*_| compared to complex I and II, which is expected if more N-bases are ligated to the Cu^II^ than in the former case. The pulsed EPR analysis also confirmed the involvement of 2-benzylpyridine (**1**) ligation in the mononuclear species IV (see ESI†). The influence of acetic acid on the N-coordination of **1** is negligible (Fig. S13 and S14†). Furthermore, it has been reported that substitution of one N by one C in copper(ii) porphyrins significantly lowers the *g*_*z*_ value and increases |*A*_*x*,*y*_| to values significantly different from those observed for species III and IV.^35^ Hence, ligand to catalyst coordination through a Cu–C bond can be discarded in species III and IV.

**Fig. 7:**
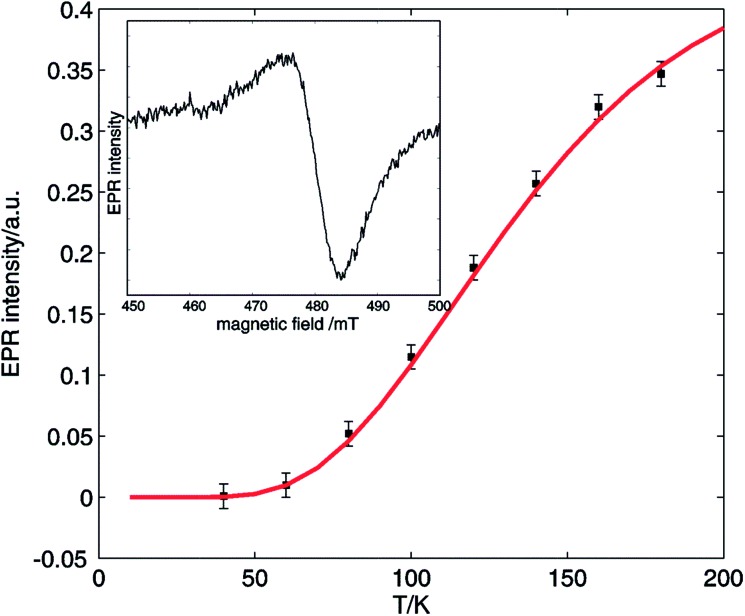
Temperature evolution of the EPR intensity of one of the high-field EPR signals typical for copper dimers (■) formed in the reaction mixture: 2-benzylpyridine (**1**) 0.5 M, 0.05 M CuI, 0.5 M acetic acid and DMSO heated to 100 °C under O_2_ for 1 h. The red curve shows the fit using eqn (3). The inset shows the high-field EPR signal used for the evaluation. The spectra were recorded using a microwave frequency of 9.73 GHz and a microwave power of 0.047 mW.

Additionally, EPR signals in agreement with dinuclear copper species could be found in the reaction mixture at all times during the reaction. Fig. 7 shows a temperature analysis of one of the high-field EPR signals from copper dimers displayed in the inset. The EPR intensity variation can be fit using the Bleaney–Bower equation (eqn (3)), yielding a value for the exchange coupling *J* of –306 cm^–1^.^36^
3

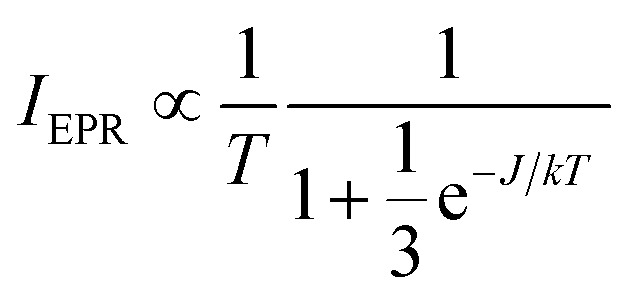




This is larger in absolute value than the one found for the copper acetate dimer, agreeing with the earlier observation that addition of pyridine to copper carboxylates yields dinuclear Cu_2_(O_2_CR)_4_py_2_ complex with increased |*J*| value.^31^ Furthermore, slight changes in the position of EPR peaks are observed, indicative of a change in the *D* values of the triplet state. The changes in *J* and *D* prove the presence of dinuclear copper species with ligation of 2-benzylpyridine (**1**), in accordance with Cu_2_(OAc)_4_(**1**)_2_ (species VI).

Interestingly, mononuclear complexes III and IV are also found in the EPR spectra of the reaction mixture using a higher copper concentration of 0.25 M (Fig. S5†). However, here the contribution of species III dominates the EPR spectrum even after 4 h of reaction (Table 2, entries 7 and 8, spectrum not shown), while it disappears within the first hour in the reaction mixture with a 0.05 M copper concentration (Fig. 6B, Table 2, entries 3–6). This can be rationalized when considering that the Cu(OAc)_2_(**1**)_2_ complex (IV) is readily formed for high [**1**]/[Cu^II^] and [AcOH]/[Cu^II^] ratios, as is the case for our standard reaction conditions. However, when the copper concentration is increased, the relative amount of acetate per copper is lower and mononuclear copper complexes involving **1** and/or DMSO with I^–^ or HO^–^ as anions, instead of **1** and acetate may be formed at higher relative quantities.

Complex V is only resolved in the EPR spectra of the reaction mixture with low copper content after a longer (4 h) reaction time (Table 2, entry 6, Fig. 6B). Since the [**2**]/[Cu^II^] ratio can already be significant at this stage of the reaction, the ligation of **2** to the copper complex becomes competitive. In the reaction with high copper content, the [**2**] may be too small for the complex to be formed. Unfortunately, the copper amount was too high at this stage of the reaction for both mixtures to allow for pulsed EPR analyses to verify this possibility (too fast electronic relaxations). However, CW-EPR spectra recorded of a reference mixture of CuI, AcOH and ketone **2** revealed the presence of a type-V species (see ESI, Fig. S6–S7†) supporting our hypothesis. The trend in the EPR parameters suggests a higher number of nitrogen ligands in the case of species V than for species III and IV, suggesting an extra ligation of **2** to a copper ion that is already ligated to two other nitrogen ligands **1**.^37^ The assignment of species I–VI is summarized in Chart 1

**Fig. 8:**
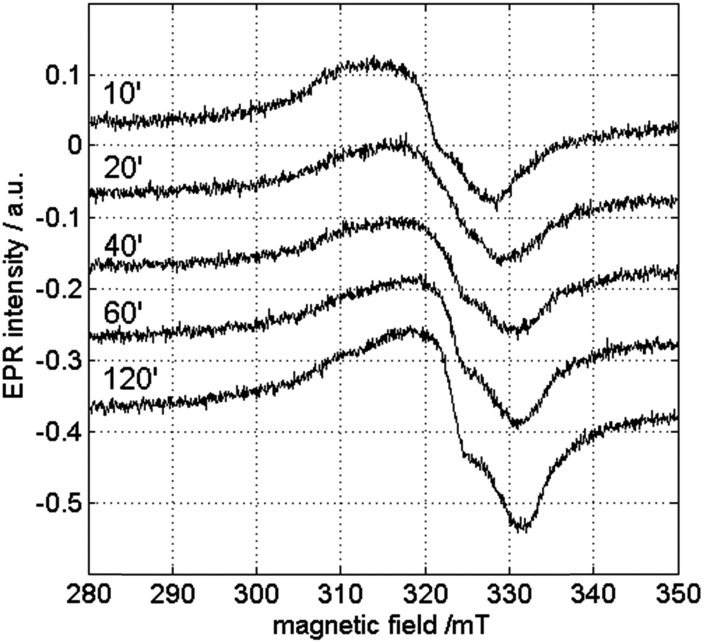
Room-temperature X-band CW-EPR spectra of the copper-catalyzed oxidation of **1** with 0.05 M CuI recorded in function of time. The spectra are recorded with a microwave power of 0.047 mW and a microwave frequency of 9.73 GHz.

**Fig. 9:**
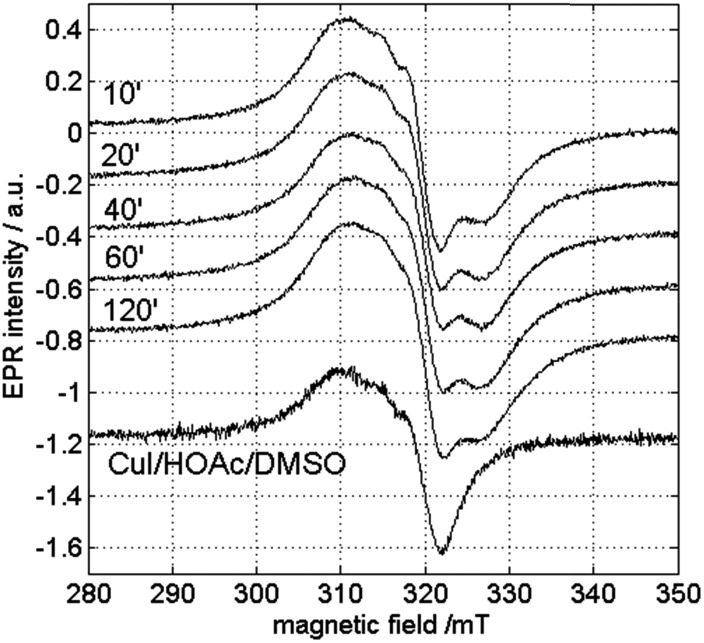
Room-temperature X-band CW-EPR spectra of the copper-catalyzed oxidation of **1** with 0.25 M CuI in function of time. At the bottom, the corresponding spectrum of the reaction mixture without the substrate **1** is shown. The spectra are recorded with a microwave power of 0.047 mW and a microwave frequency of 9.73 GHz.

In order to check the influence of the measurement temperature on the occurrence of the different copper species, CW-EPR experiments were performed at different temperatures. Fig. 8 and 9 show the room-temperature CW-EPR spectra of the copper-catalyzed oxidation of 2-benzylpyridine (**1**) with starting concentrations of 0.05 M and 0.25 M CuI, respectively, recorded on aliquots of the reaction mixture taken at different times in the reaction. Again, none of the spectra show contributions of organic radicals, confirming the low-temperature EPR results and the kinetic experiments using radical scavengers. In line with the low-temperature EPR (Fig. 6 and S5†), a significant variation of the EPR spectrum can be seen during the course of the reaction involving a lower copper loading due to contributions of species III and IV (Fig. 8), whereas a negligible variation is seen when a higher amount of copper is used (Fig. 9) (see also detailed comparison in ESI, Fig. S8 and S9†). Comparison of the latter spectra with the EPR spectrum of the solution without substrate **1** (bottom Fig. 9) suggests a strong contribution of Cu^II^ complexes that are related to species I and II. This is surprising, since no contributions of these species were observed in the spectra at 100 K (Fig. S5†). This indicates that the equilibrium of reaction (4) shifts to the right as temperature decreases.
4

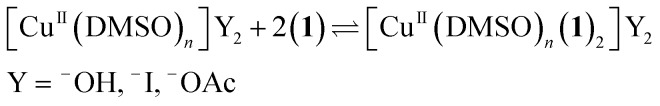




A high [**1**]/[Cu^II^] ratio evidently also pushes the equilibrium to the right, explaining why the contribution of species III and IV can still be seen and I and II are less dominant in the room-temperature spectra of the reaction mixture at lower CuI concentration (Fig. 8).

Although clear evidence of the presence of dinuclear species in the reaction mixture with 0.05 M CuI (Table 2, entries 3–6) and the CuI/DMSO/AcOH system (Table 2, entry 2) is found at low temperatures (Fig. S2† and 7), no signals due to dimers are observed in the EPR spectra at room temperature (Fig. S10†). This is not due to the absence of the dimers, but is simply due to spectral broadening effects as reported earlier by others for dinuclear copper acetate species.^38^ Large Cu^II^ concentrations are required to be able to observe the typical EPR features stemming from dinuclear copper species at room temperature (Fig. S11,† signals indicated with arrows). The amount of dinuclear species is clearly increasing during the reaction time. Furthermore, the signal change at a later stage of the experiment (see for instance the appearance of a peak around 480 mT) is indicative of variation in the types of dinuclear complexes that are formed under these circumstances. This points to a variation in the axial ligands L of Cu_2_(OAc)_4_L_2_; **1**, **2** or solvent molecules. Further proof for these acetate bridged dinuclear Cu^II^ species was obtained using infrared spectroscopy. When working at increasing concentrations of added CuI, new peaks started to arise in the infrared spectrum of the reaction mixture. By deconvolution of the overall IR spectrum a new IR spectrum could be obtained. The resulting spectrum matches with that of Cu(OAc)_2_·2H_2_O in DMSO which is known to exist as a dinuclear species and a reported dinuclear acetate bridged Cu^II^ complex (see Fig. S23 and S24†).^39^ Furthermore, increasing amounts of CuI cause a decrease in the concentration of free AcOH in the reaction mixture. This can be monitored by following the decrease in area of the C=O bond vibration of AcOH (around 1718 cm^–1^). At the same time an increase in intensity is observed for the vibration appearing at 1623 cm^–1^ (see Fig. S25 and S26†). The lowering of the wavenumber of AcOH can be rationalized by its complexation, bridging two copper centers, hereby weakening it's C=O bond.

### Catalytic cycle

**Fig. 10:**
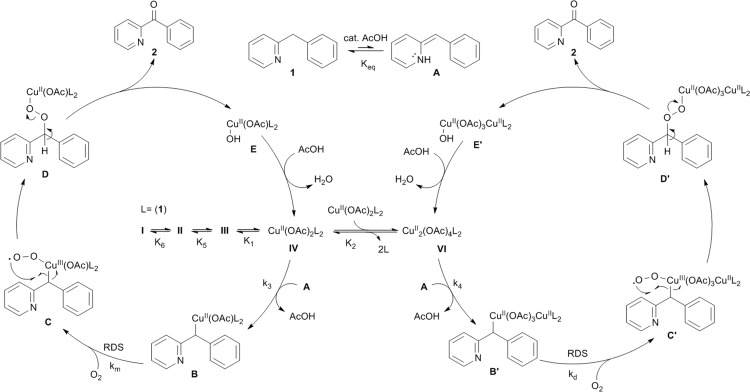
Mechanistic proposal for the aerobic Cu-catalyzed oxidation of **1** into **2**.

Based on all the insight gathered a catalytic cycle is proposed in Fig. 10. Aerobic oxidation of the added CuI brings the catalyst to the active +II oxidation state (complexes **I–IV**). An initial acid-catalyzed imine–enamine tautomerization, characterized by the equilibrium constant *K*_eq_ transforms the substrate **1** into the enamine tautomer **A**. Nucleophilic attack of **A** on an electrophilic Cu^II^ center (monomer **IV** or dimer **VI**) produces a C–Cu^II^ bond (**B** or **B′**). Acetic acid acts as a catalyst for the tautomerization. Although adding an acid does not change the equilibrium between **1** and **A**, it will lower the activation energy, thereby increasing the rate by which enamine **A** is replenished. All these steps are reversible and in rapid equilibrium characterized by their respective equilibrium constants. Their relative concentrations depend on the initial reaction conditions (the ratios between all the reaction components: substrate, catalyst, acid and solvent). In the next step the Cu^II^ center that is bound to the substrate molecule will be oxidized by O_2_ to a highly reactive Cu^III^-superoxide species (**C** or **C′**).^40^,^41^ The oxidation of **B**/**B′** is thought to be the rate determining step and it is herein that lies the difference in reaction rate for mononuclear (*k*_m_) *versus* dinuclear (*k*_d_) catalyst species (*k*_m_ > *k*_d_). The superoxide species will quickly rearrange *via* a radical pathway with an electron transfer to the catalyst producing the Cu^II^ organoperoxide **D** or **D′**. Heterolytic dissociation of these organoperoxides by deprotonation of the remaining benzylic proton produces the reaction product **2** and recovers the catalyst with a hydroxide rather than an acetate bound (**E** or **E′**).^42^**E** and **E′** can be converted back to **IV** and **VI** by anion exchange with acetate, hereby producing one equivalent of water.^43^

The amount of O_2_ required to make one molecule of ketone was determined by performing gas uptake experiments. It was found that when using CuI (10 mol%) as the catalyst the O_2_ : substrate stoichiometry is ∼1 (see ESI†). These results confirm that in accordance with our mechanistic proposal for each molecule of product one molecule of water is formed and that no extra oxygen is required to oxidize the catalyst during the catalytic cycle.

For our proposed catalytic cycle a kinetic isotope effect is expected even though C–H/D bond cleavage is not included in the rate-determining step. As the C–H bond cleavage (*via* imine–enamine tautomerization) is reversible and its *k*_–1_ is much faster than the *k*_2_ of the RDS (oxygenation), the observed KIE is the equilibrium isotope effect.^44^ When comparing the initial rates of the reaction on **1** using AcOH with that of **1**-d_2_ using AcOD-d_4_ a KIE value of 4.28 was found (Fig. S19†). As an additional experiment we tested if deuterium could be build-in *via* tautomerization under our reaction conditions. When mixing 2-benzylpyridine with d_4_-AcOD in deuterated DMSO in the absence of catalyst and O_2_ the singlet of the benzylic protons started to disappear in ^1^H-NMR and a broad triplet resulting from **1**-d_1_ started to appear further supporting the imine–enamine tautomerization (see ESI Fig. S20†). In contrast, diphenylmethane (**8**) could not be deuterated in the same manner as expected.

The EPR experiments (Table 2) indicate that the catalyst resting state is species **IV** with even at low [catalyst] a significant contribution of dinuclear species **VI**. When the [catalyst] is increased the equilibrium is significantly shifted towards the dinuclear species **VI**. Because the equilibrium between the imine (**1**) and enamine (**A**) tautomers is highly shifted towards the imine (**1**) the concentration of species **B** and **B′** is expected to be very low which explains why these species were never directly observed by EPR measurements. The reason why the dinuclear species oxidize slower than the mononuclear is unclear. The simplest explanation is the increased steric bulk of the catalyst (4 bridging acetates). However it is also possible that the second Cu^II^ in **C′** reacts with the formed Cu^III^-superoxide species producing a less reactive dioxygen bridged species.

Formation of side product **3** can occur *via* protonation of intermediate **D** or **D′** producing a hydroperoxide that can undergo a Fenton like Cu^I^-catalyzed homolytical cleavage of the O–O bond yielding alcohol **3** and a hydroxyl radical. This radical is known to be very reactive and thus very short-lived.^45^ Alcohol **3** is formed in such small quantities so that an in-depth study of the mechanism of its formation was not possible. Alcohol **3** can be transformed into **2** by copper-catalyzed β-hydride elimination (Scheme 2).^16^

## Conclusions

This research has provided deeper insight into the mechanism of Cu^II^-catalyzed aerobic oxygenations. Although the mechanism of Cu-catalyzed aerobic alcohol oxidation has been thoroughly studied, kinetic studies on the corresponding methylene oxidation are unprecedented. Only reactions on stoichiometric Cu-complexes with oxygen have been reported. In this study we have shown that the substrates are activated *via* an initial acid catalyzed imine–enamine tautomerization. DFT calculations can be used as useful method to predict the equilibrium constant (*K*_eq_) and can act as a tool to qualitatively predict whether or not a substrate can be oxidized. Alcohol **3** was excluded as an intermediate in this reaction and is a side product presumably generated by the homolytical breaking of the O–O bond in the hydroperoxide formed from complex **D**/**D′***via* protonation. Kinetic studies revealed O_2_ mass transfer limitation effects limiting the turnover of the reaction at high concentrations of substrate and acetic acid. A complex kinetic behavior was observed when increasing the catalyst loading. At intermediate loadings mass transfer is again occurring resulting in a maximum rate. The continued decrease of the reaction rate at higher concentrations of catalyst however can't be explained by the occurrence of mass transfer alone. When using transition metal catalysis in combination with (acetate) bridging ligands dinuclear species need to be considered. Although these are usually considered to be related to catalyst deactivation this study shows this is not necessarily the case as they explain the further reduction in rate observed at a higher concentration than at which the mass transfer determined *v*_max_ occurs. The formation of a dinuclear catalytically active species which is less reactive than the mononuclear catalyst explains the non-zero reaction rate at increased concentration of added catalyst. Such behavior is to the best of our knowledge unprecedented in literature. In addition to these kinetic experiments we performed an in-depth EPR-study to characterize the catalytic species present in the reaction. These measurements confirmed the presence of both different mono- and dinuclear Cu^II^-species. The results presented in this paper can directly be used in the mechanistic study of other active methylene oxygenations where the methylene moiety is activated by a tautomerization step.

## Supplementary Material

Supplementary information
