# Supplementary material for: Mechanism of the CuII-catalyzed benzylic oxygenation of (aryl)(heteroaryl)methanes with oxygen
Source: Chem Sci. 2015 Sep 29;7(1):346–57. doi: 10.1039/c5sc03530a (PMC5952523; doi:10.1039/c5sc03530a)
Supplement: Supplementary file 1 [file SC-007-C5SC03530A-s001.pdf]

Electronic Supplementary Material (ESI) for Chemical Science.
This journal is © The Royal Society of Chemistry 2015

Supporting information

Mechanism of the Cu^{II}-Catalyzed Benzylic Oxygenation of (Aryl)(heteroaryl)methanes with Oxygen

Hans Sterckx[1], Johan De Houwer[1], Carl Mensch[1], Ignacio Caretti[2], Kourosch Abbaspour Tehrani[1], Wouter A. Herrebout[1], Sabine Van Doorslaer[2], Bert U.W. Maes[1,*]

[1] Department of Chemistry, University of Antwerp, Groenenborgerlaan 171, B-2020 Antwerp, Belgium
[2] Department of Physics, University of Antwerp, Universiteitsplein 1, B-2610 Antwerp, Belgium

Table of Contents **Page**

I.	General considerations	S2
II.	EPR measurements	S2
III.	Methods for *in situ* IR measurements	S14
IV.	Involvement of radical intermediates?	S22
V.	Gas-Uptake measurements	S23
VI.	Derivation of the rate laws	S23
VII.	Synthetic experimental procedures and NMR data	S28
VIII.	DFT calculation of equilibrium constants and bond dissociation energies	S32
IX.	References	S72
X.	NMR spectra	S73

I. General considerations

Melting points were determined on an automated apparatus and are uncorrected. The ^{1}H NMR and ^{13}C-NMR spectra were recorded on a 400 MHz spectrometer with TMS as the internal standard in the solvent indicated. Coupling constants are given in Hertz and the chemical shifts are given in ppm. For high resolution mass-spectrometric analysis, samples were dissolved in CH_3OH and diluted to a concentration of approximately 10^{-5} mol/L. 2 µL Was injected using the CapLC system and electrosprayed through the nanoelectrospray source. The nanoelectrospray source was operated in positive ion mode at an electrospray potential of 1.7 kV. The eluent used was 30% A (H_2O 0.1% formic acid) and 70% B (CH_3OH/H_2O 95/5 0.1% formic acid) at a flow rate of 6 µL/min. Samples were injected with an interval of 3 minutes. Before analysis and after each seventh sample a 2 µL volume of a 0.025% H_3PO_4 solution (50/50 $MeOH/H_2O$) was injected and used as lock mass. The MS was calibrated prior to use with a 0.015% H_3PO_4 solution. The spectra were lock mass corrected using the known mass of the nearest H_3PO_4 cluster. Flash column chromatography was performed on an automated chromatography system with silica flash cartridges. All chemicals, which are not described in the experimental part, were obtained from commercial sources and used as such. The oxygen used was standard industrial oxygen.

II. EPR measurements

Experimental conditions

The X-band continuous wave (CW) EPR experiments were performed either on a spectrometer with a microwave (mw) frequency of ~9.44 GHz (Bruker ESP300E) or a spectrometer with a mw frequency of 9.76 GHz (Bruker E580 Elexsys), both equipped with a liquid helium cryostat. The EPR spectra were recorded with a modulation amplitude of 0.5 mT and a modulation frequency of 100 kHz. All other experimental details are given in the figure captions. For the room-temperature measurements, the solutions were inserted in a glass capillary to avoid spectrometer tuning problems. Blank samples were recorded (solvent only) and used to subtract the background signals.

Pulsed EPR experiments were performed on a Bruker E580 Elexsys spectrometer equipped with equipped with a gas-flow cryogenic system (Oxford Instruments) allowing for operation from room temperature down to 4 K. A pulse repetition rate of 1kHz was used. The HYSCORE (hyperfine sublevel correlation spectroscopy) experiments were performed using the $\pi/2$-τ-$\pi/2$-t_1-π- t_2-$\pi/2$-τ-echo sequence with pulse lengths $t_{\pi/2}$= 16 ns, t_π= 32 ns.[S1] The times t_1 and t_2 were varied from 96 to 4880 ns in steps of 16 ns. Experiments were performed for different τ values as specified in the figure captions.

The Davies-ENDOR (electron nuclear double resonance) experiments were performed using the microwave pulse sequence π-T-$\pi/2$-τ-π-τ-echo using different settings for the microwave pulses (see figure S4 caption).[S2] While a radiofrequency (rf) pulse of 10 µs was applied for the measurements after 10 min of reaction, an rf pulse of 5 µs needed to be applied for the reaction mixture at 1 h (due to faster electron relaxations). An ENI A-300 rf amplifier was used.

Simulations of all EPR spectra were performed with the EasySpin program, a MATLAB toolbox developed for EPR simulations.[S3] The HYSCORE data were processed with MATLAB 7.7.0. The time traces were baseline-corrected with a third-order polynomial, apodized with a Hamming window and zero-filled. After a two-dimensional Fourier transformation, the absolute spectra were computed. Spectra recorded with different τ values were added after Fourier transformation to eliminate blind-spot effects.

Additional Results

Chart 1. Mono- and dinuclear copper species present in the reaction

$[Cu^{II}(DMSO)_n]X_2$	$Cu^{II}(DMSO)_2(OAc)_2$	$[Cu^{II}(DMSO)_n(\mathbf{1})_2]Y_2$
**I**	**II**	**III**
$Cu^{II}(OAc)_2(\mathbf{1})_2$	$Cu^{II}(OAc)_2(\mathbf{1})_2(\mathbf{2})$	$(\mathbf{1})Cu^{II}(OAc)_4Cu^{II}(\mathbf{1})$
**IV**	**V**	**VI**

X = HO^-, I^-
Y = HO^-, I^-, AcO^-
n = 2, 3, 4

Table S1. Relative contributions of the different species detailed in Table 2 to the observed EPR signal of the mononuclear complexes as obtained from simulation.

Entry		[CuI] (M)	Heating time	Species	Relative contribution (%)
1	CuI/DMSO	0.05	10’	I	100
2	CuI/DMSO/AcOH	0.05	10’	I’	62
				II	38
3	CuI/DMSO/AcOH /(**1**)	0.05	5’	III	15
				IV	85
4	CuI/DMSO/AcOH /(**1**)	0.05	10’	III	11
				IV	89
5	CuI/DMSO/AcOH /(**1**)	0.05	1 h	IV	100
6	CuI/DMSO/AcOH /(**1**)	0.05	4 h	IV’	50
				V	50
7	CuI/DMSO/AcOH	0.25	10’	IV	25
				III	75
8	CuI/DMSO/AcOH	0.25	4 h	IV	20
				III	80

a) Control experiments

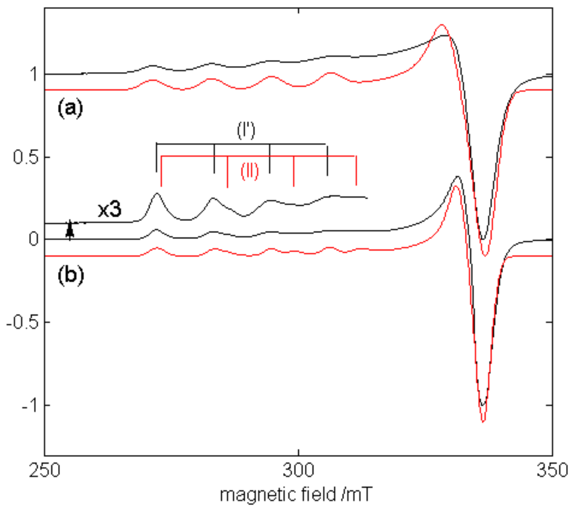

***Figure S1**. Experimental (black) and simulated (red) CW-EPR spectra of (a) 0.05 M CuI in DMSO and (b) 0.05 M CuI and 0.5 M AcOH in DMSO after 10 minutes heating to 100 ^{o}C under O_2. The spectra were recorded at 100 K with a microwave power of 0.47 mW and a microwave frequency of 9.73 GHz. They are shown normalized to facilitate comparison. The low-field part of spectrum (b) is shown enhanced to indicate the contributions of species I' and II.*

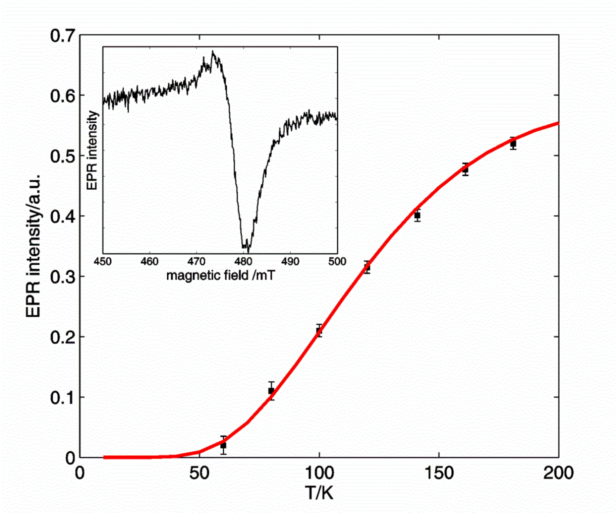

***Figure S2**. Temperature evolution of the EPR intensity of one of the high-field EPR signals typical for copper acetate dimers (■) in a solution of CuI (0.05 M) and AcOH (0.5 M) in DMSO. The red curve shows the fit using $I_{EPR} \propto \frac{1}{T}\frac{1}{1+\frac{1}{3}e^{-J/kT}}$. The inset shows the high-field EPR signal used for the evaluation. The spectra were recorded using a microwave frequency of 9.73 GHz and a microwave power of 0.047 mW.*

b) Identification of Cu-(**1**) ligation in species IV

The involvement of 2-benzylpyridine (**1**) ligation in the monomeric species IV can be derived from pulsed EPR experiments. Figure S3 shows the ^{1}H HYSCORE spectrum recorded at 282.8 mT (low field edge of spectra of species IV) for the reaction mixture with low copper content after 1 h in comparison with the spectrum taken of the solution of CuI (0.05 M) and AcOH (0.5 M) in DMSO under the same conditions. We clearly see that addition of 2-benzylpyridine (**1**) has led to the observation of new cross peaks that are analogous to the signals of the ortho protons of pyridine observed for copper-pyridine complexes.[S4] Similar results were obtained at other magnetic field settings (not shown). Davies ENDOR spectra using hard pulses have been shown to suppress the signals of the weakly coupled nuclei (here the ^{1}H contributions) and thus enhance the contributions of the strongly coupled nuclei.[S5] In Figure S4 we make use of this effect to reveal the interaction with the ^{14}N nuclei for species III and IV. Figure S4a,b shows the Davies ENDOR spectra of the reaction mixture with low CuI concentration (0.05 M) recorded after 10' of reaction at 282.8 mT. In the ENDOR spectrum recorded with soft pulses (Figure S4a) signals due to the weakly coupled protons and ^{13}C nuclei (in natural abundance) are visible. These interactions could also be detected by HYSCORE (not shown). There seems to be a signal around 15 MHz overlapping with the ^{1}H contribution. When hard pulses are used, the ^{1}H contribution is fully suppressed, leaving a signal at 15-17 MHz agreeing with the interaction with the contribution of at least one ^{14}N nucleus with hyperfine value of ~32 MHz. Similar signals were observed at other magnetic field settings, although overlap with ENDOR lines of the $^{63/65}Cu$ interactions are observed at the higher field orientations (^{Cu}A is in the order of 20-35 MHz at these observer postions (Table 2)). Although the fast electron relaxations (due to the higher Cu^{II} amount) render the recording of ENDOR spectra more difficult at later stages in the reaction, it is clear that the Davies ENDOR spectrum recorded with hard pulses for the reaction mixture after 1 h (where only species IV is contributing) still shows the presence of the ^{14}N ENDOR signals, confirming that also in species IV, at least one nitrogen base is coordinating to the copper ion.

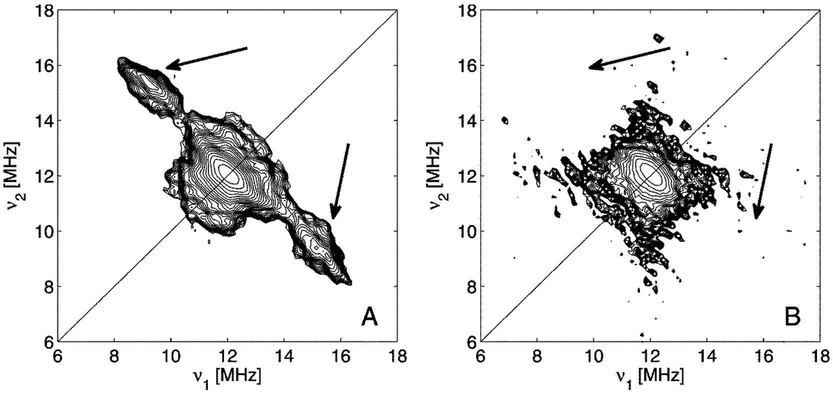

***Figure S3**. ^{1}H HYSCORE spectra of (A) the reaction mixture with 0.05 M CuI and 0.5 M **1** after 1 hour at 100 °C under O_2 (species IV) and (B) a solution of CuI (0.05 M) and AcOH (0.5 M) in DMSO (species I and II). The arrows indicate the position of the ortho-protons of the pyridine segment of **1**. The spectra were*

recorded at 8 K, and represent the sum of the spectra recorded with τ values 96 and 176 ns. The spectra were recorded at 282.8 mT, a position corresponding with the lowest field edge of the EPR spectrum of species IV.

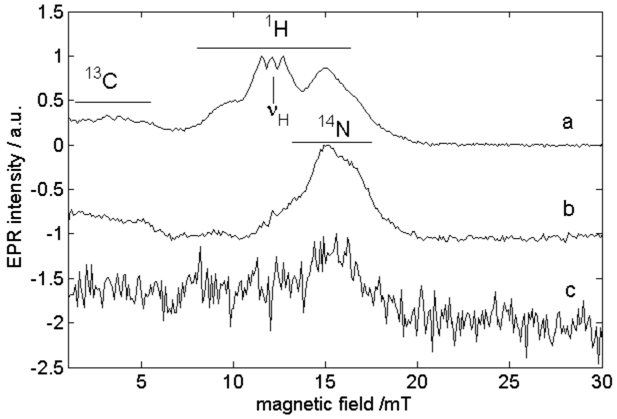

***Figure S4**. Davies ENDOR spectra recorded at 282.8 mT and 8 K for the reaction mixture with 0.05 M CuI and 0.5 M 2-benzylpyridine (**1**), 0.5 M AcOH in DMSO (1 mL) heated to 100 °C under O_2 for (a,b) 10 min and (c) 1 h. (a) Spectrum recorded with soft pulses ($t_{\pi/2}$=80 ns, t_{π}=160 ns), (b,c) Spectrum recorded with hard pulses ($t_{\pi/2}$=24 ns, t_{π}=48 ns). The signals due to the interactions of the unpaired electron of Cu^{II} with the ^{13}C, ^1H and ^{14}N nuclei are indicated.*

c) Influence of [Cu] on copper-complex formation

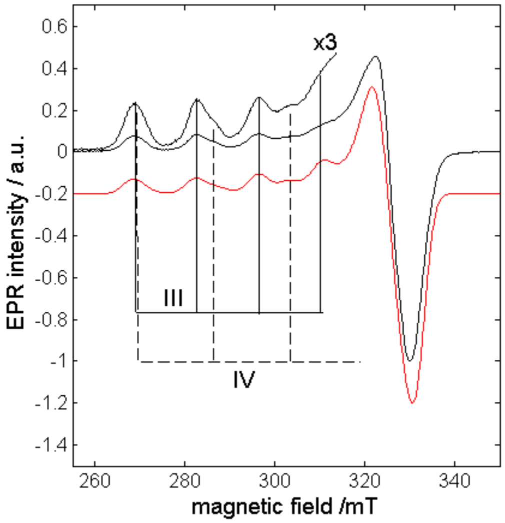

***Figure S5**. Experimental (red) and simulated (black) CW-EPR spectra of the reaction mixture with 0.5 M CuI and 0.5 M 2-benzylpyridine (**1**), 0.5 M AcOH in DMSO (1 mL) heated to 100 °C under O_2 for 10 minutes. The spectra were recorded at 100 K with a microwave power of 1 mW and a microwave frequency of 9.43 GHz.*

d) Analysis of possible Cu-(**2**) ligation

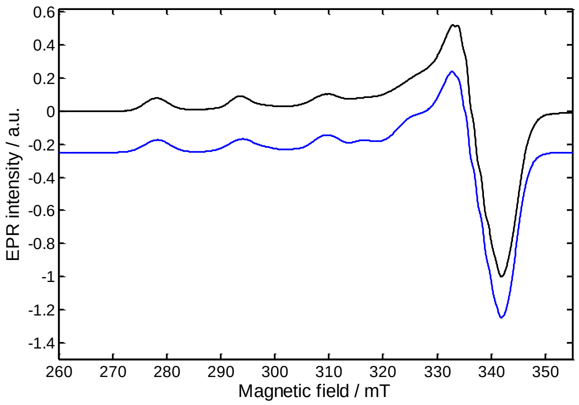

Figure S6. *Experimental (black) and simulated (blue) CW-EPR spectra recorded at 100 K of a solution of CuI (0.05 M) and AcOH (0.5 M) in DMSO with 2-benzoylpyridine (**2**) heated for 10' at 100 °C under O_2 atmosphere. The [CuI]/[(**2**)] ratio is 0.1. The species used for simulation are shown in table S2. The spectra were recorded with a microwave power of 0.47 mW and a microwave frequency of 9.73 GHz.*

Figure S6 shows the CW-EPR spectrum of a solution of CuI (0.05 M) and AcOH (0.5 M) in DMSO with the product 2-benzoylpyridine (**2**) (0.25 M) that was heated for 10 min at 100 °C to mimic the reaction conditions. Simulation of the experiment revealed the presence of four species: IV", III', V and VII (see Table S2). Figure S7 illustrates that a contribution of species V needs to be taken into account to simulate the experimental spectrum, *proving that species V may indeed be related to one of the binding modes of the reaction product **2** to copper*. The EPR parameters of III' and IV" are similar to the ones of species III and IV found after addition of **1** to the solution (see manuscript). This indicates similar binding modes in both cases (i.e. the direct binding of the pyridine nitrogens of two ligands to the copper ion). The *g* and hyperfine values of species V seem to indicate a larger ligation of nitrogen ligands (see manuscript). The EPR parameters of VII are in the same order of those found for IV and IV', and may indicate a small change in the ligation mode (**2** may bind copper either only via the pyridine nitrogen (as in IV') or may bind via both the carbonyl oxygen and pyridine nitrogen). Species III', IV'' and VII may be present in the actual reaction mixture (spectra recorded after 4h, Figure 6, manuscript), but are hard to distinguish from those of species III and IV.

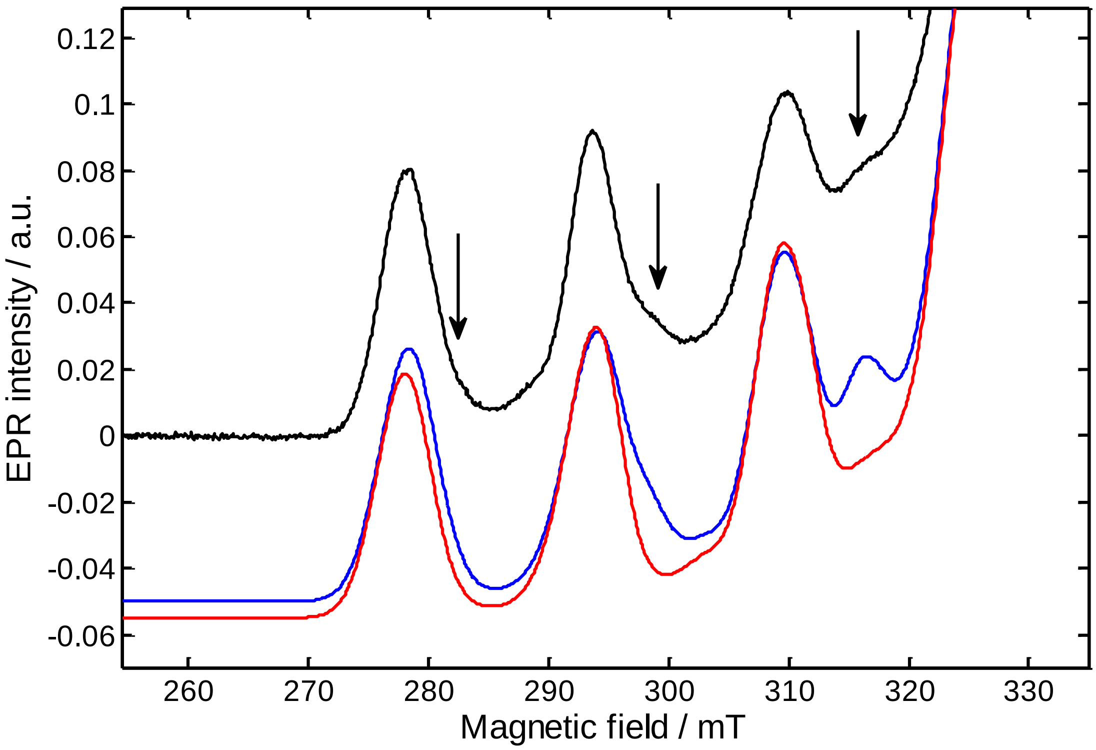

Figure S7. *Enlarged view of the low-field area of the expertimental (black) and simulated (blue) spectra shown in Fig. S6, together with a second simulated spectra (red) without considering species V. The arrows indicate the EPR intensity due to the* $^{Cu}A_z$ *hyperfine splitting of species V.*

Table S2. Principal *g* and copper hyperfine values of the mononuclear Cu^{II} complexes observed in the spectra of Figure S6.

**Entry**		**[CuI] (M)**	**Heating time**		g_x **±0.002**	g_y **±0.002**	g_z **±0.001**	**|A_x| (MHz) ±15**	**|A_y| (MHz) ±15**	**|A_z| (MHz) ±5**	**%**
**1**	CuI/DMSO/ AcOH/**2**	0.05	10’	**VII**	2.052	2.052	2.290	45	45	520	6
				**III’** [a)]	2.071	2.072	2.345	25	25	430	4
				**IV’**	2.063	2.064	2.305	35	35	495	75
				**V**	2.067	2.067	2.262	35	35	560	15

The last column gives the relative contribution of each component to the simulated spectrum. [a)] For the simulation of species III in the reaction mixture with starting concentration [CuI] = 0.05 M, the interaction with two ^{14}N nuclei with an isotropic hyperfine coupling of 40±5 MHz was considered,

e) Room temperature EPR analysis

Figure S8 shows the difference spectra obtained by subtracting the contribution of the substrate-less mixture from the EPR spectra of the reaction mixture with high copper content in Figure 9. For comparison, the spectra are overlaid with the spectra of mixtures with low copper content. At 10 minutes in the reaction, both spectra are similar. However, as the reaction evolves, the significant evolution in the EPR features found for the low [Cu] sample, is not observed in the difference spectra. In order to understand this evolution, Figure S9 shows the comparison of the spectra of the mixture with low copper content after 10′ and 120′ in the reaction, with the simulations of the room-temperature EPR spectra of species I-V, assuming a rotational correlation time of 0.4 10^{-10} s and line widths of 5 mT. From this comparison, it becomes clear that at early stages in the reaction, mainly species I,II and III can be observed, while the contributions of species IV and V start to dominate the spectra at longer reaction times. In the reaction mixture at high CuI concentration, no evidence of contributions of IV and V are found. In contrast, large amounts of dinuclear copper species can be found at high [Cu] (Figs. S10, S11; see also main text for further explanations).

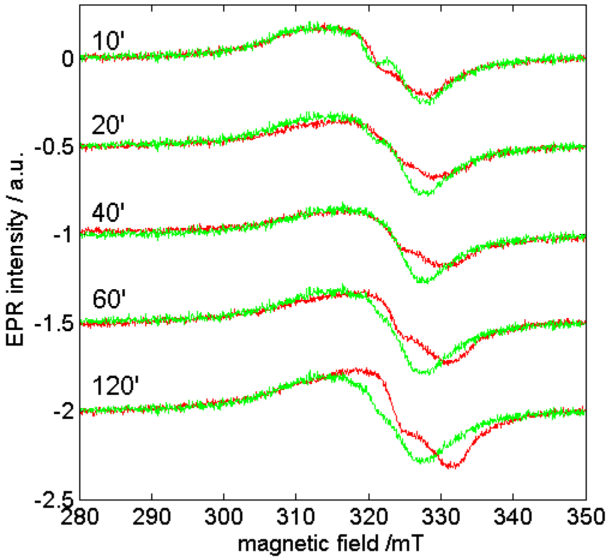

Figure S8. *Comparison between (red) the room-temperature EPR spectra of the copper-catalyzed oxidation of 2-benzylpyridine (**1**) with 0.05 M CuI recorded at different times after the beginning of the reaction and (green) the room-temperature EPR spectrum obtained by subtracting the spectrum of the substrate-less solution (bottom of Figure 9) from those obtained for the reaction mixture with 0.25 M CuI. The red spectra were scaled by a factor of 2 to allow comparison.*

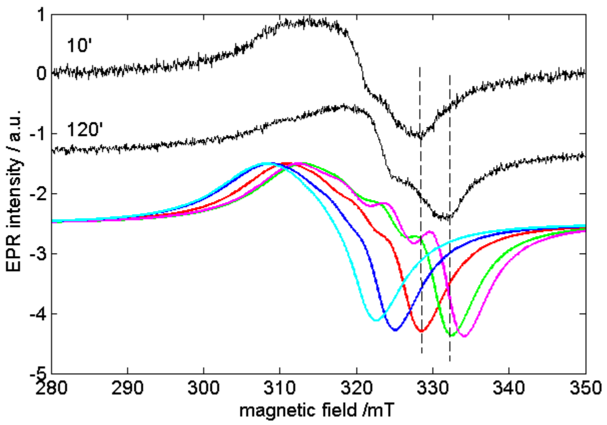

Figure S9. *Comparison between the experimental room temperature EPR spectra of the copper-catalyzed oxidation of 2-benzylpyridine (**1**) with 0.05 M CuI recorded at 10' and 120' after the beginning of the reaction (black) and the simulated EPR spectra of species I (cyan), II (blue), III (red), (IV) green and (V) magenta. The spectra were obtained using the g and copper hyperfine values of Table 2, and assuming a rotational correlation time of 0.4 10^{-10} s and linewidths of 5 mT.*

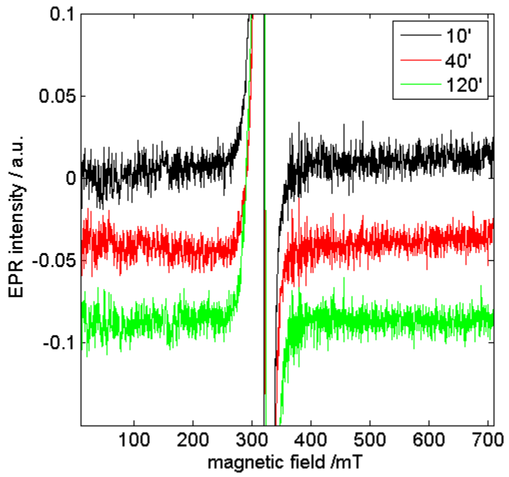

Figure S10. *Room temperature X-band EPR spectra of the copper-catalyzed oxidation of 2-benzylpyridine (**1**) with 0.05 M CuI recorded at different times in the reaction. The spectra are recorded with a microwave power of 15 mW. For each of the spectra, the background signal due to the Cr^{3+} signal of the cavity and the Fe^{III} signal of the glass capilary was subtracted.*

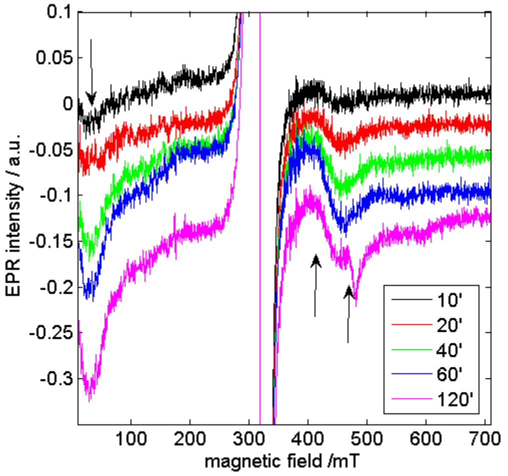

Figure S11*. Room temperature X-band EPR spectra of the copper-catalyzed oxidation of 2-benzylpyridine (****1****) with 0.25 M CuI recorded at different times in the reaction. The arrows indicate the features typical for dimeric copper acetate species. The spectra are recorded with a microwave power of 15 mW. For each of the spectra, the background signal due to the Cr^{3+} signal of the cavity and the Fe^{III} signal of the glass capilary was subtracted.*

f) Influence of the solvent

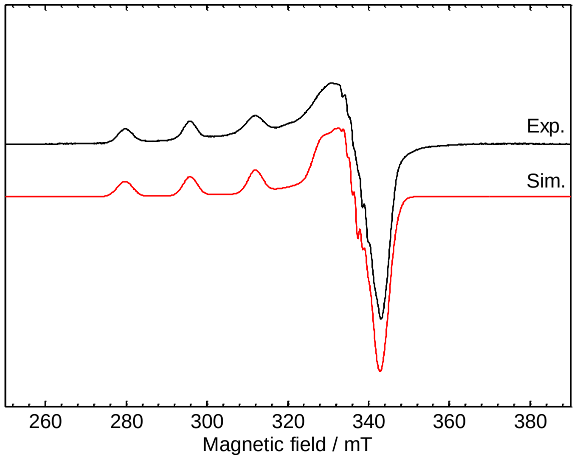

Figure S12. *X-band CW-EPR experimental (black) and simulated (red) spectra recorded at 100K of the copper-catalyzed oxidation of 2-benzylpyridine (****1****) in toluene after 5' reaction with 0.05 M CuI.*

Table S3. Principal *g* and copper hyperfine values of the mononuclear Cu^{II} complexes observed in the spectrum of Figure S12.

Entry		[CuI] (M)	Heating time		g_x ±0.002	g_y ±0.002	g_z ±0.001	$\|A_x\|$ (MHz) ±15	$\|A_y\|$ (MHz) ±15	$\|A_z\|$ (MHz) ±5	%
**1**	CuI/Tol/AcOH /(**1**)	0.05	1h	**IV''**	2.060	2.060	2.285	45	45	505	65
				**IV''** [a]	2.065	2.065	2.285	30	30	505	14

The last column gives the relative contribution of each component to the simulated spectrum. [a] For the simulation of of species IV in the reaction mixture with starting concentration [CuI] = 0.05 M, the interaction with two ^{14}N nuclei with an isotropic hyperfine coupling of 35±5 MHz was considered.

The copper-catalyzed oxidation of 2-benzylpyridine (**1**) was carried out in toluene instead of DMSO under the same experimental conditions at a concentration of 0.05 M CuI. The X-band CW-EPR spectrum of the reaction mixture after 5' was measured at 100 K (Figure S12). The ***g*** and ***A*** parameters obtained from simulation are summarized in Table S3. Two species IV''with similar values to species IV and IV' are detected after 5', whilst no DMSO-related species are observed (species III). This result confirms the ligation of DMSO to Cu when the latter solvent is used in the reaction.

g) Analysis of the effect of AcOH on the affinity of Cu-N ligation between **1** and catalyst

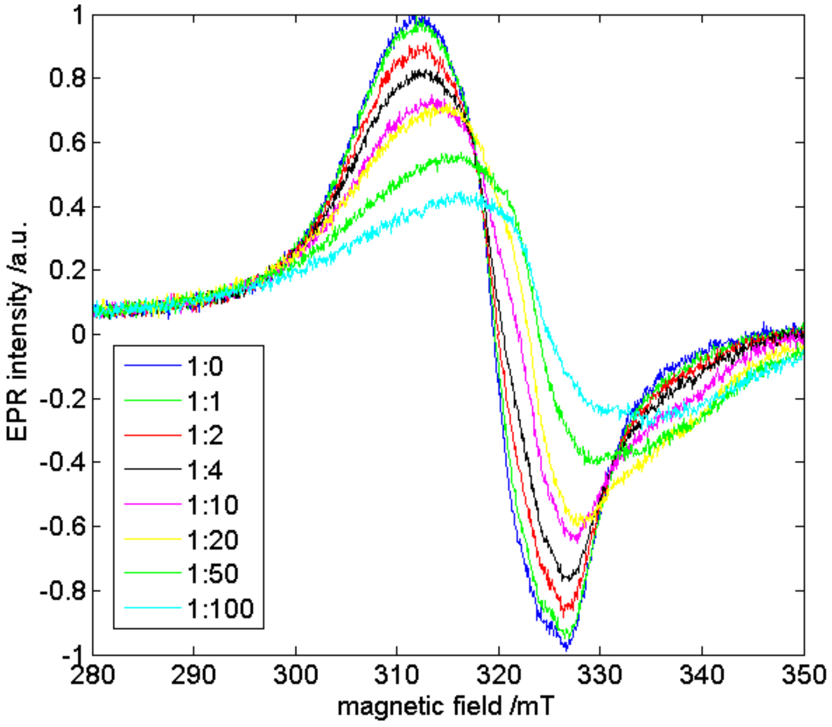

Figure S13. *Room-temperature X-band EPR spectra of 0.05 M CuBr$_2$ in DMSO with increasing 2-benzylpyridine (**1**) concentration. The inset legend shows the [Cu]:[**1**] ratio.*

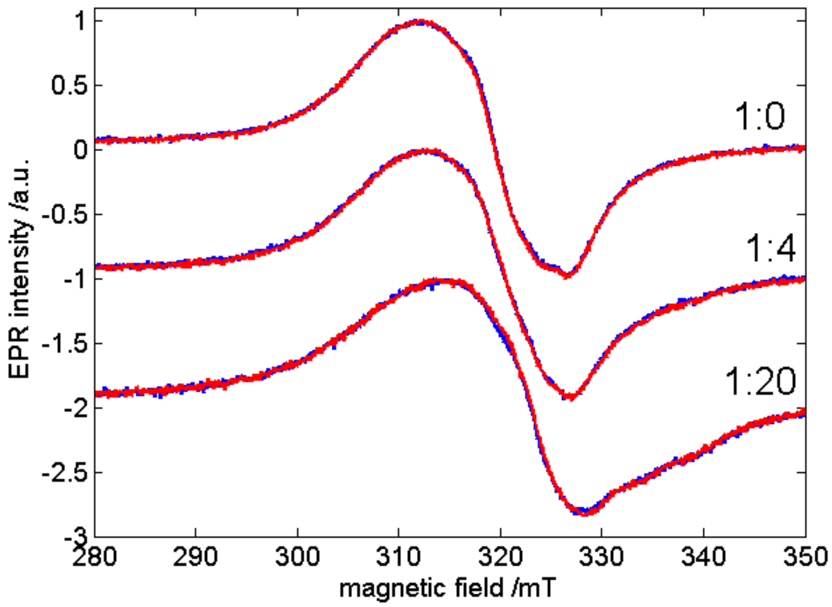

Figure S14. *The room-temperature X-band EPR spectra of 0.05 M $CuBr_2$ in DMSO with different [Cu]:[**1**] ratios. The spectra from solutions with (red) and without (blue) 0.05 M acetic acid are shown for three [Cu]:[**1**] ratios.*

Figure S13 illustrates the EPR spectral changes observed when increasing amounts of **1** are added to a 0.05 M $CuBr_2$ solution in DMSO. The complex spectral changes reflect the gradual replacement of coordinated DMSO by 2-benzylpyridine ligands in the copper complexes. The spectra cannot be described as simple combinations of two components, in line with the expected occurrence of copper complexes with zero up to four 2-benzylpyridine ligands, each with their specific EPR signature. Figure S14 shows that addition of acetic acid has no effect on the spectral features of these solutions. Hence, for a given [Cu]:[**1**] ratio the same proportionality of the different copper complexes is found, irrespective of the presence of acetic acid. This implies that acetic acid does not have an effect on the binding affinity of **1** to Cu^{II} in the catalytic reactions under study.

III. Methods for *in situ* IR measurements

a) general information

Infrared reaction monitoring experiments were performed on a Matrix-MF and a ReactIR spectrometer using respectively a Diamond ATR fiber probe (IN355, Ø 6.3 mm) and a custom made (DST, Ø 6.35 mm) DiComp AgX probe.

b) IR-spectra of the reaction components

The initial reaction rate was determined by following the formation of 2-benzoylpyridine (**2**) over time. More specifically, the carbonyl C=O stretch of the ketone was chosen as a viable peak for integration due to the fact that it is intense and has no overlap with peaks of other reaction components. Integration was executed for the full area under the curve (integration mode A) between 1653 cm^{-1} and 1685 cm^{-1} (Figure S12, top left, marked in blue).

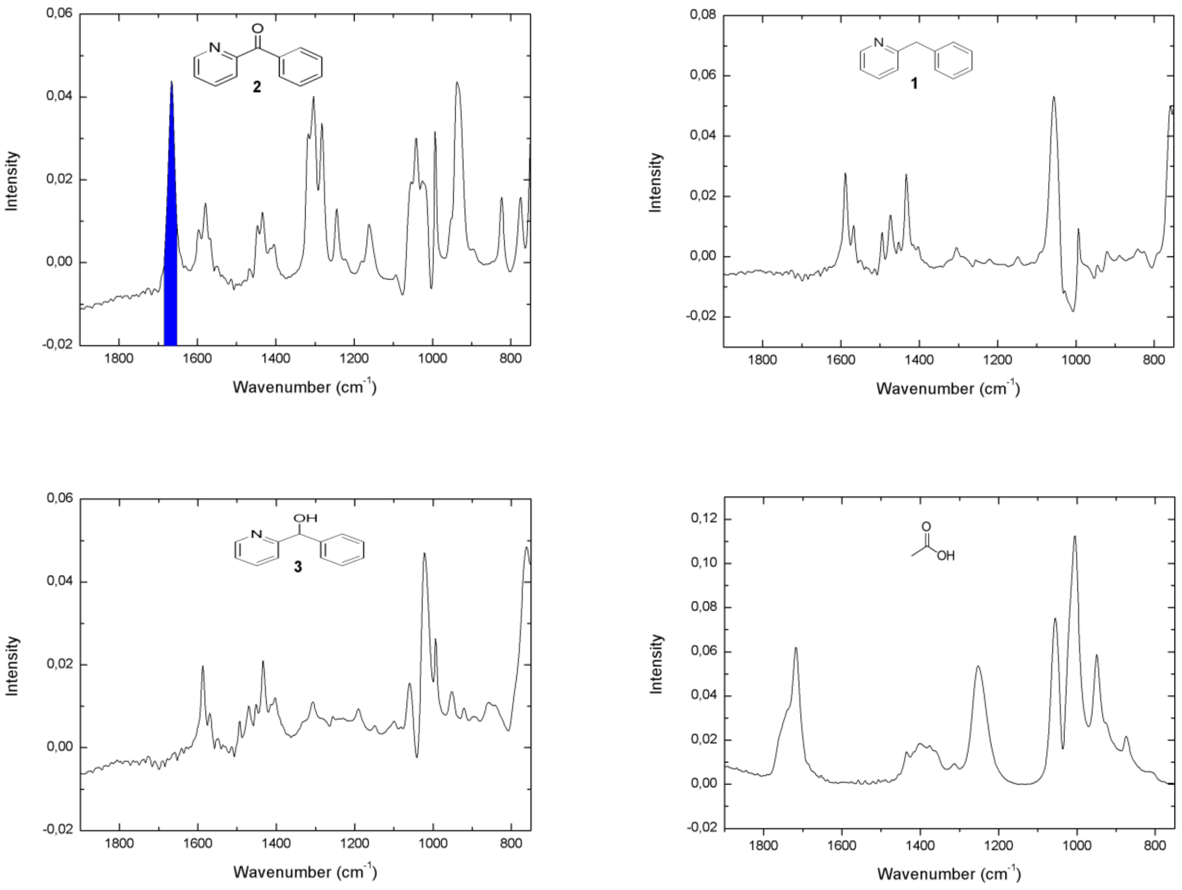

Figure S15. *IR spectra of the different reaction components. Top left: 2-benzoylpyridine (**2**), top right: 2-benzylpyridine (**1**), bottom left: phenyl(pyridin-2-yl)methanol (**3**), bottom right acetic acid.*

c) General synthetic method for in-situ IR measurements

A reaction tube (Ø = 2.5 cm, l = 16.5 cm) was charged with CuI (0.095 g, 0.5 mmol, 10 mol%), 2-benzylpyridine (5 mmol), DMSO (10 mL) and acetic acid (0.300 g, 5 mmol). The flask was flushed with O_2 for 1 min, closed with a septum through which the probe was brought as well as a balloon filled with O_2 (the latter pierced through the septum via a needle). The mixture was placed in a preheated oil bath and stirred at 100 °C. At this point the reaction starts and the monitoring software is started.

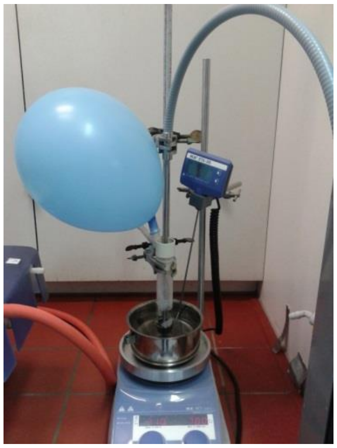

* Different O_2/N_2 mixtures were made to determine the reaction order of O_2. These mixtures were made using a pressure manifold by pressurizing an evacuated steel gas cylinder with pure O_2 to a pressure of X bars followed by adding N_2 to a total pressure of 12 bar.[S6] The ratio O_2/N_2 then equals X/(12-X).
* For the experiments with addition of TEMPO, 1 equivalent (5 mmol) of TEMPO was added to the reaction mixture.
* To measure the influence of the stirring rate a PTFE coated octagonal magnetic stirring bar (Ø = 5 mm, l = 20 mm) with a pivot ring tube (Ø = 6 mm) was used in combination with an RCT basic stirring plate.

d) Calibration curve for 2-benzoylpyridine

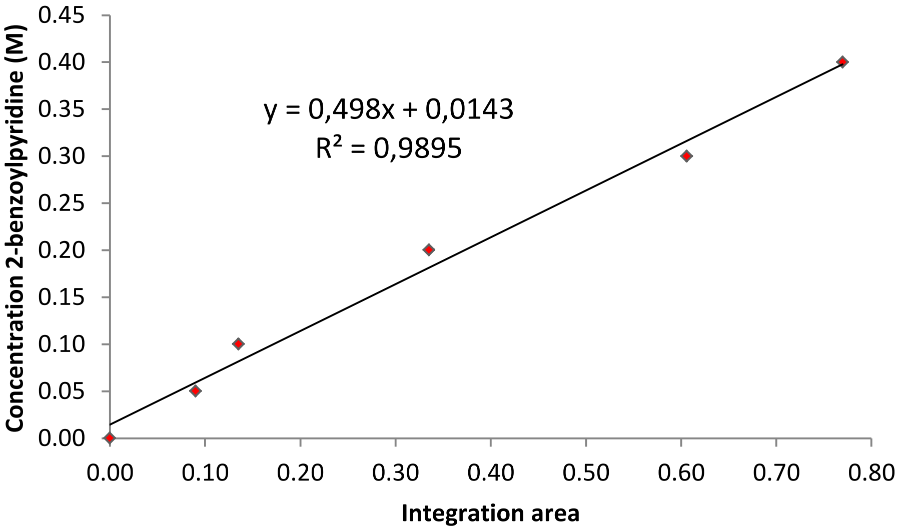

e) Preequilibrium versus mass transfer limitation of O_2

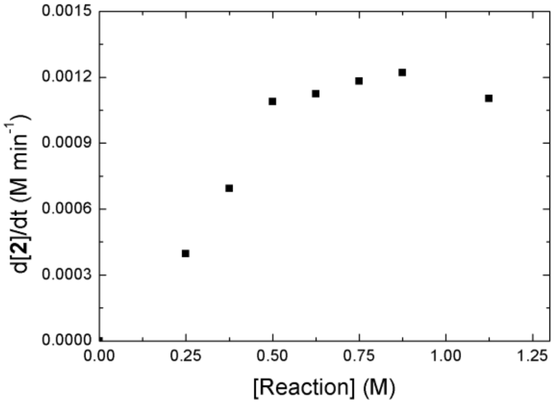

Figure S16. *Dependence of the initial rate of the copper-catalyzed oxidation of 2-benzylpyridine (**1**) on the reaction concentration. The concentration of the x-axis represents the [**1**], the ratios [**1**]/[Cu] and [**1**]/[AcOH] are kept constant. Reaction conditions: **1** (0.25 – 1.125 mmol), CuI (10 mol %), AcOH (1 eq.), O_2 (balloon), DMSO (10 mL), 100 °C.*

f) Determination of Kinetic Isotope Effect (KIE)

To determine the kinetic isotope effect double deuterated substrate (**1**-D_2) was synthesized by dissolving **1** in 25 mL of D_2O and adding 0.5 mL of DCl and heating the mixture to 180 °C for 3 hours under microwave irradiation. After the reaction is complete saturated aq. $NaHCO_3$ (20 mL) is added and the mixture is extracted with diethylether (3 x 20 mL). The organic fractions are collected and dried over $MgSO_4$. **1**-D_2 was isolated in 94 % yield (± 95 % benzylic deuteration).

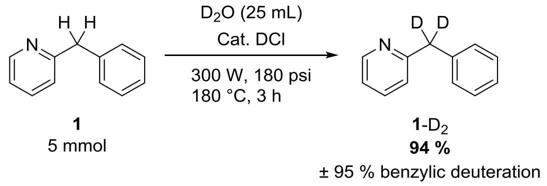

Figure S17. *Synthesis of double benzylic deuterated 2-benzylpyridine (**1**-D_2).*

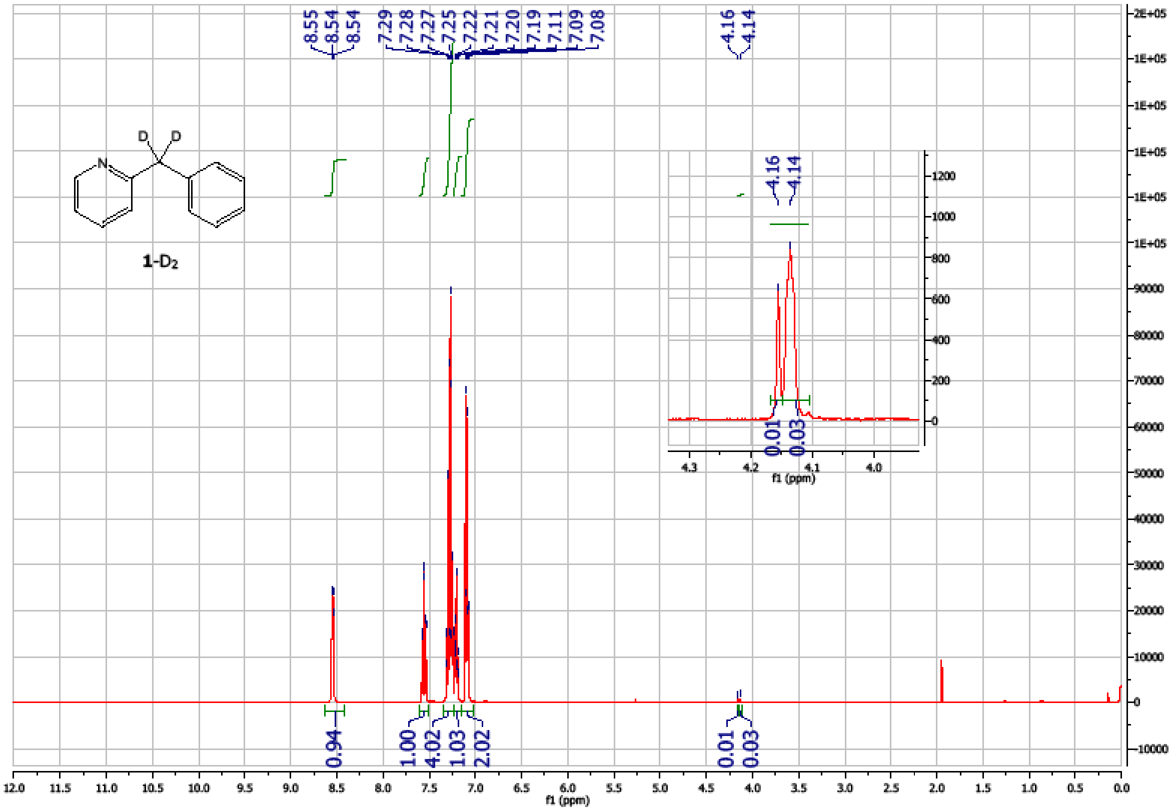

Figure S18. *^{1}H-NMR of double benzylic deuterated 2-benzylpyridine (**1**-D_2).* **^{1}H-NMR ($CDCl_3$, 400 MHz)** δ_H: 8.54 (d, 1H, J = 4.2 Hz), 7.55 (dt, 1H, J = 7.7, 1.8 Hz), 7.33-7.24 (m, 4H), 7.23-7.17 (m, 1H), 7.12-7.07 (m, 2H). The singlet of the benzylic protons at 4.16 ppm has almost completely dissapeared and a very small broad triplet, due to mono-deuteration, has appeared at 4.14 ppm.

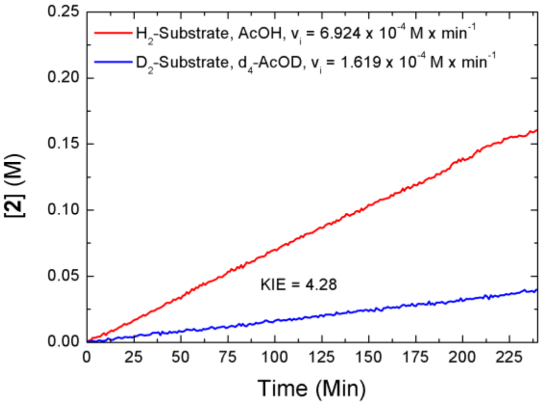

Figure S19. *Determination of kinetic isotope effect using in-situ IR spectroscopy. Reaction conditions:* ***1*** *(0.1 mmol) or* ***1****-D_2 (0.1 mmol), CuI (10 mol %), AcOH (1 eq.) or d_4-AcOD (1 eq.), O_2 (balloon), DMSO (2 mL), 100 °C in a 10 mL microwave vial with a stirring rate of 1000 rpm.*

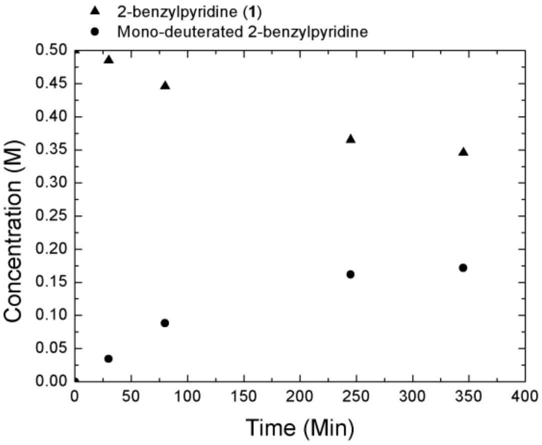

Figure S20. *Conversion versus time of the deuteration of 2-benzylpyridine (**1**) with d_4-AcOD. Reaction conditions:* ***1*** *(5 mmol), d_4-AcOD (1 eq.), Ar (balloon), d_6-DMSO (10 mL), 100 °C. Samples were taken at the time indicated and ^{1}H-NMR was measured immediately. For the concentration of 2-benzylpyridine (**1**) the dissapearance of the singlet at 4.16 ppm was integrated. For the concentration of mono-deuterated 2-benzylpyridine the triplet at 4.16 was integrated. Double benzylic deuterated 2-benzylpyridine (**1**-D_2) can not directly be detected by ^{1}H-NMR.*

g) Catalyst decomposition?

The possibility of catalyst decomposition was examined by adding another equivalent of substrate after the first reaction was completed (18 hours). The reaction starts up again without any significant loss in reaction rate and evolves to completion. After 42 hours the reaction mixture was worked-up and 92 % (9.2 mmol) of 2-benzoylpyridine (**2**) was isolated. This indicates that no detectable catalyst degradation is taking place.

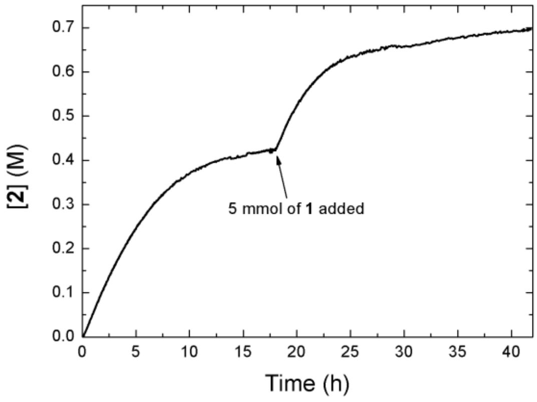

Figure S21. *The effect of adding another batch (5 mmol) of substrate (**1**) after the first reaction is complete. Reaction conditions: **1** (5 mmol), CuI (10 mol %), AcOH (1 eq.), O_2 (balloon), DMSO (10 mL), 100 °C.*

To determine whether formation of side product **3** has any influence on the catalytical species present in the reaction, the reaction was doped with 20 mol% of alcohol **3** and its effect on the initial rate was examined. Identical initial reaction rates were measured for both reactions. From this can be concluded that alcohol **3** has no influence on the catalytical species present.

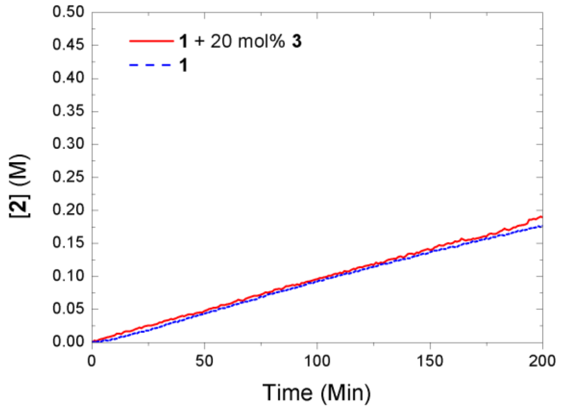

Figure S22. *The effect of adding 20 mol% of alcohol **3** at the start of the reaction. Reaction conditions: **1** (5 mmol), **3** (20 mol%), CuI (10 mol %), AcOH (1 eq.), O_2 (balloon), DMSO (10 mL), 100 °C.*

h) Further proof for existence dimeric catalytic species

In addition to the EPR-analysis, *in-situ* IR was used to support the existence of dimeric catalytical species. The carbonyl stretch vibration of acetic acid located at 1718 cm^{-1} (Figure S23) decreases over time. This decrease is caused by the formation of dinuclear acetate bridged Cu^{II} species. This is supported by the

fact that the decrease at 1718 cm^{-1} is accompagnied by an increase of a vibration at 1623 cm^{-1}. This vibration corresponds to the carbonyl stretch vibration of acetate ions in dinuclear Cu^{II} complexes like $Cu(OAc)_2 \cdot 2H_2O$ in DMSO (Figure S24). Integration of the area of the peak at 1718 cm^{-1} therefore allows us to follow the concentration of free AcOH over time, additionally integration of the area at 1623 cm^{-1} allows us to monitor the concentration of AcOH caught up in a dinuclear complex. When the initial concentration of CuI was increased more AcOH was used and subsequently more dinuclear complex formed (Figure S25 and S26).

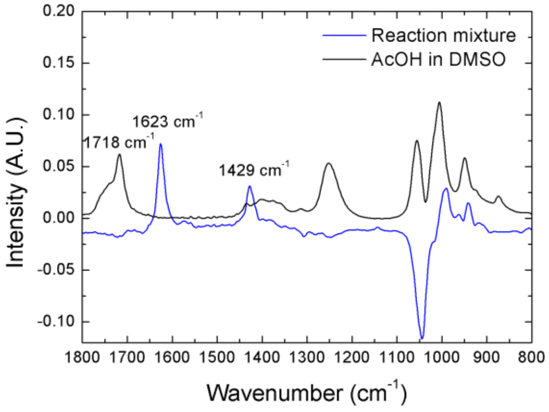

Figure S23. *Deconvoluted infrared spectrum of the reaction mixture (using 0.35 M of CuI) showing the occurrence of new peaks (blue) and the infrared spectrum of acetic acid in DMSO (Black) as a reference.*

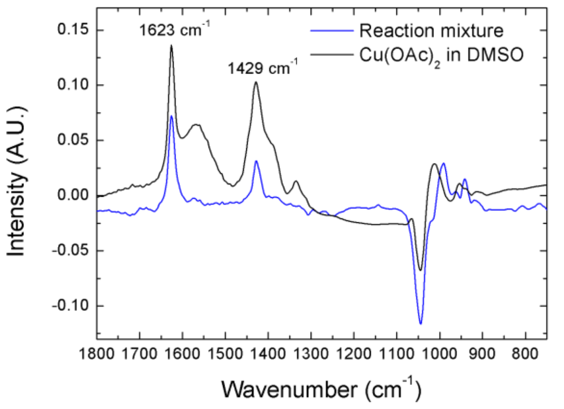

Figure S24. *Deconvoluted infrared spectrum of the reaction mixture (using 0.35 M of CuI) (blue) and the infrared spectrum of $Cu(OAc)_2 \cdot 2H_2O$ (0.35 M) in DMSO (black) as a reference. $Cu(OAc)_2$ is known to exist as a dinuclear species.*

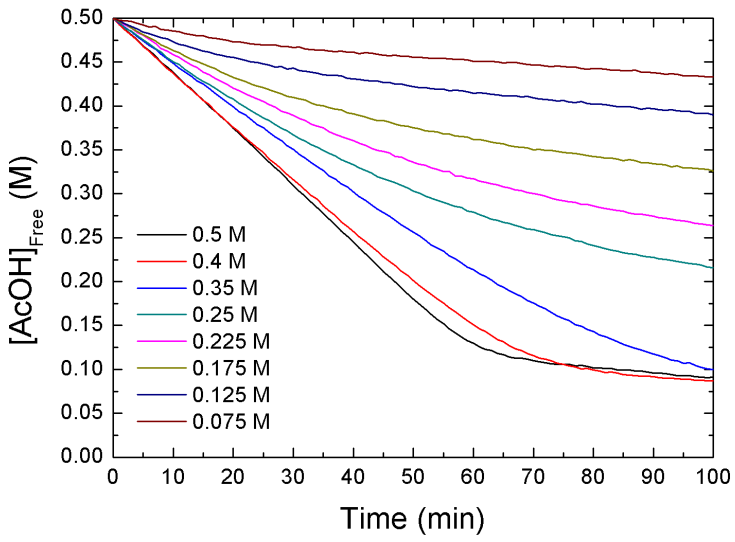

Figure S25. *The concentration of free acetic acid over time in the reaction mixture at different initial CuI concentrations. The curves are the result from calibrated integration of the area under the peak from 1790 cm^{-1} – 1680 cm^{-1}corresponding to the carbonyl stretch vibration of free AcOH.*

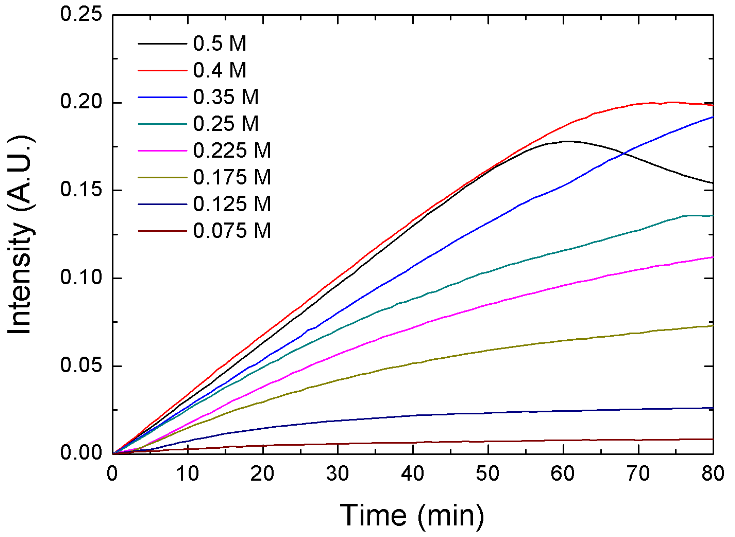

Figure S26. *Change in intensity (~concentration) over time in the reaction mixture at different initial CuI concentrations. The curves are the result from integration of the area under the peak from 1640 cm^{-1} – 1600 cm-^{1} corresponding to the carbonyl stretch vibration of acetic acid incorporated in a dinuclear Cu-complex.*

IV. Involvement of radical intermediates?

To determine whether or not radical intermediates are formed during the catalytic cycle several control experiments were performed. First the effect of addition of a few known radical inhibitors on the rate of the reaction was examined. TEMPO and 1,1-diphenylethylene were selected as radical inhibitors and their addition did not slow down the reaction neither could any adducts be detected. In addition to these two liquid radical inhibitors activated carbon was added which is known to act as a radical scavenger. The addition of activated carbon did not influence the conversion of the reaction after 4 hours (Figure S27). This reaction could not be monitored with *in-situ* IR spectroscopy due to the tendency of activated carbon to stick to the probe. In addition to these kinetic experiments no organic radical could be detected via EPR-spectroscopy. Any sufficiently stable organic radical (such as a benzylic radical), even in very low quantities, should be detectable by EPR. Finally a radical clock type molecule (2-{[2-(prop-2-en-1-yl)phenyl]methyl}pyridine (**26**)) was synthesized and oxidized under our standard conditions. If a benzylic radical would be formed it immediately cyclizes in a 5-exo-trig fashion. This reaction is expected to be very fast and outcompetes the intermolecular trapping by O_2. This ring-closed product could however not be detected and the expected ketone [2-(prop-2-en-1-yl)phenyl](pyridin-2-yl)methanone (**27**) was isolated in 54% yield. Based on the combination of these experiments we can exclude involvement of radical intermediates.

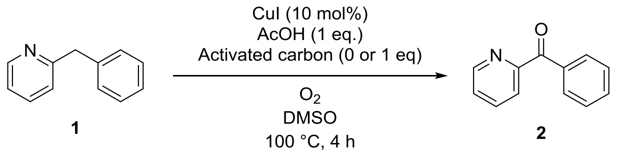

No activated Carbon: 47 % (47 % of **1** recovered)
With activated Carbon: 47 % (48 % of **1** recovered)

Figure S27. *The effect of adding 1 eq. of activated carbon at the start of the reaction on the isolated yield after 4 hours. Reaction conditions:* ***1*** *(0.5 mmol), Activated Carbon (1 eq.), CuI (10 mol %), AcOH (1 eq.),* O_2 *(balloon), DMSO (1 mL), 100 °C.*

V. Gas-Uptake measurements

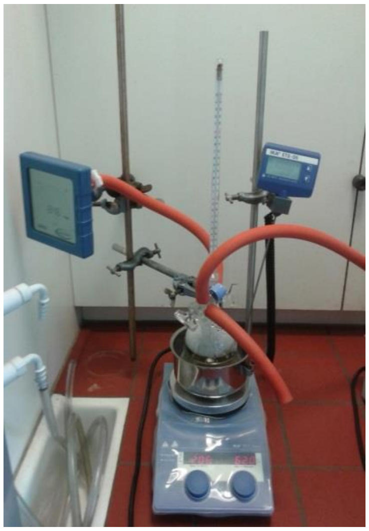

A home-made three-necked round bottomed flask (total volume = 366 mL) with one neck equipped with a valve was attached to a manometer to monitor the pressure. The second neck was fitted with a thermometer to monitor the temperature of the headspace. The remaining one was connected to a three-way valve connected to a vacuum pump and an oxygen bottle. The flask was charged with 2-benzylpyridine (0.846 g, 5.00 mmol), CuI (0.095 g, 0.50 mmol) and DMSO (10 mL). The reaction mixture was brought in an oil bath set at 100°C under stirring, until the temperature of the headspace remained constant. Next the headspace was flushed for 1 min with O_2 and acetic acid (0.286 mL, 5.00 mmol) was added via a syringe. The valve was closed and the initial pressure was measured (P_i). After 4 hours of reaction the final pressure was noted (P_e) and the flask was removed from the oil bath and opened. The reaction mixture was allowed to reach room temperature and the general work-up was performed. An ^{1}H-NMR yield of the reaction product using 1,3,5-trimethoxybenzene as the internal standard was determined giving the number of moles of reaction product formed (n_{end}). The average of three experiments was taken to determine the stoichiometric ratio.

$$\frac{\Delta n_{O_2}}{n_{end}} = \frac{\frac{\Delta P.V}{R.T}}{n_{end}} = \frac{\frac{(P_i - P_e).V}{R.T}}{n_{end}}$$

VI. Derivation of the rate laws

- Kinetic model for saturation behavior in function of substrate (**1**) concentration

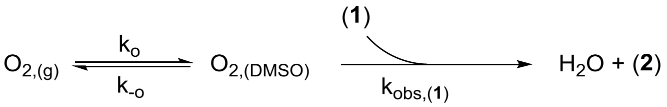

We look at the dependence of [(**1**)] on the initial rate. The [AcOH] is kept constant and we define $k_{obs,(\mathbf{1})} = k_{RDS}$[AcOH], with k_{RDS} being the rate determining step of the overall catalytic cycle.
A steady-state approximation can be made for the change in dissolved [O_2] over time:

$$\frac{d[O_2]_{DMSO}}{dt} = 0$$

$$\frac{d[O_2]_{DMSO}}{dt} = k_o[O_2]_g - k_{-o}[O_2]_{DMSO} - k_{obs,(\mathbf{1})}[(\mathbf{1})][O_2]_{DMSO} = 0$$

$$[O_2]_{DMSO} = \frac{k_o[O_2]_g}{k_{-o} + k_{obs,(\mathbf{1})}[(\mathbf{1})]}$$

The rate of formation of **2** equals:

$$\frac{d[\mathbf{2}]}{dt} = k_{obs,(\mathbf{1})}[O_2]_{DMSO}[(\mathbf{1})] = \frac{k_{obs,(\mathbf{1})}k_o[O_2]_g[(\mathbf{1})]}{k_{-o} + k_{obs,(\mathbf{1})}[(\mathbf{1})]}$$

It is clear that at low concentrations of **1**, the equation can be simplified to (with $K_o = k_o/k_{-o}$):

$$\frac{d[\mathbf{2}]}{dt} = \frac{k_{obs,(\mathbf{1})}k_o[O_2]_g[(\mathbf{1})]}{k_{-o}} = k_{obs,(\mathbf{1})}K_o[O_2]_g[(\mathbf{1})]$$

Which is a first order relation for [(**1**)]

At high concentrations of **1**, the equation can be simplified to:

$$\frac{d[\mathbf{2}]}{dt} = k_o[O_2]_g$$

Which is zeroth order in [(**1**)] and $[O_2]_g$ is constant.

- Kinetic model for saturation behavior in function of AcOH concentration

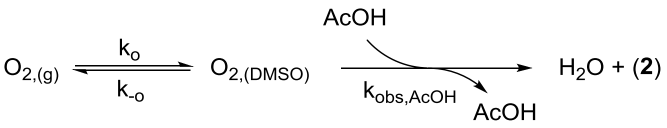

We look at the dependence of [AcOH] on the initial rate. The [(**1**)] is kept constant and we define $k_{obs,AcOH} = k_{RDS}[(\mathbf{1})]$, with k_{RDS} being the rate determining step of the overall catalytic cycle.
A steady-state approximation can be made for the change of dissolved $[O_2]$ over time:

$$\frac{d[O_2]_{DMSO}}{dt} = 0$$

$$\frac{d[O_2]_{DMSO}}{dt} = k_o[O_2]_g - k_{-o}[O_2]_{DMSO} - k_{obs,AcOH}[AcOH][O_2]_{DMSO} = 0$$

$$[O_2]_{DMSO} = \frac{k_o[O_2]_g}{k_{-o} + k_{obs,AcOH}[AcOH]}$$

The rate of formation of **2** equals:

$$\frac{d[\mathbf{2}]}{dt} = k_{obs,AcOH}[O_2]_{DMSO}[AcOH] = \frac{k_{obs,AcOH}k_o[O_2]_g[AcOH]}{k_{-o} + k_{obs,AcOH}[AcOH]}$$

It is clear that at low concentrations of AcOH, the equation can be simplified to (with $K_o = k_o/k_{-o}$):

$$\frac{d[\mathbf{2}]}{dt} = \frac{k_{obs,AcOH}k_o[O_2]_g[AcOH]}{k_{-o}} = k_{obs,AcOH}K_o[O_2]_g[AcOH]$$

Which is first order in [AcOH].

At high concentrations of AcOH, the equation can be simplified to:

$$\frac{d[\mathbf{2}]}{dt} = k_o[O_2]_g$$

Which is zeroth order in [AcOH] and $[O_2]_g$ is constant.

- Kinetic model for inhibition in function of catalyst concentration:

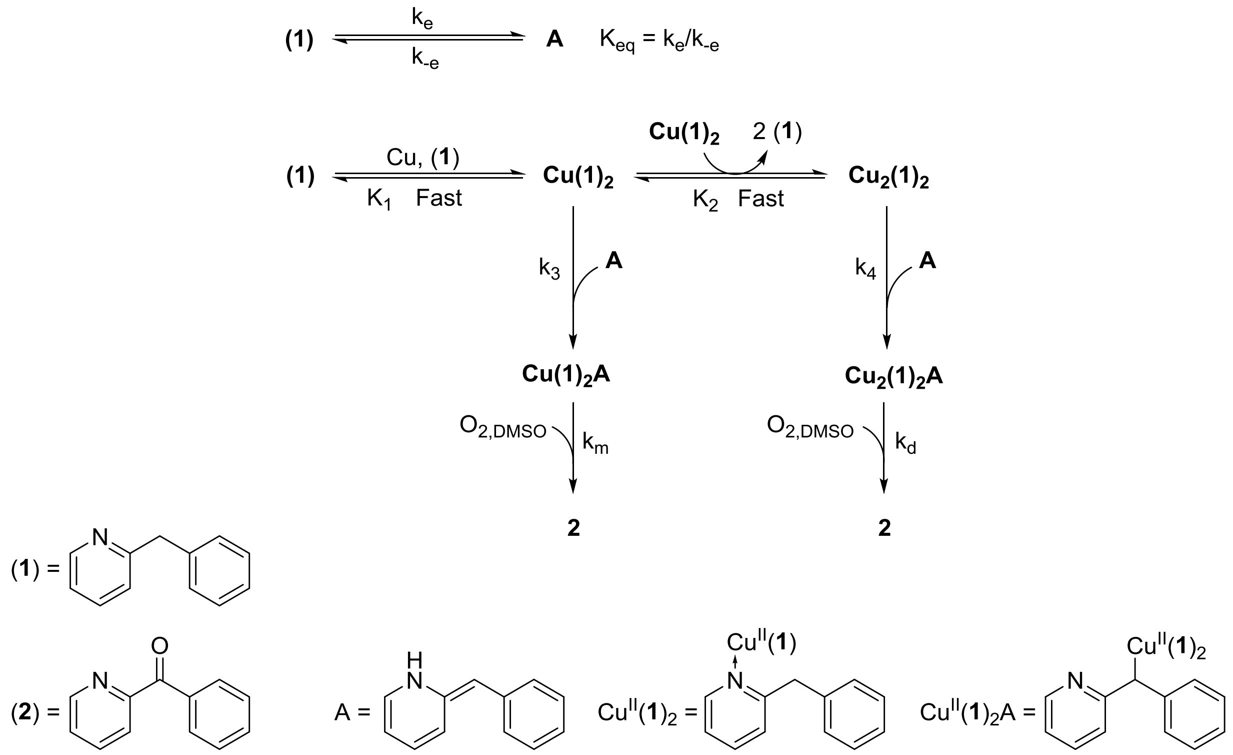

Note: If the substrate is N-ligated it acts as a ligand (**1**), in C-ligation it acts as an anion (**A**). Other anions (AcO^-, I^-) and ligands (DMSO, (**2**)) are not directly involved in the mechanism and are left out of this model for simplicity. (**1**), Cu^{II}(**1**)$_2$ and Cu^{II}_2(**1**)$_2$ are considered to be in fast equilibrium.

$$K_1, K_2, K_{eq}\ are\ equilibrium\ constants$$

$$k_e, k_{-e}, k_3, k_4, k_m, k_d\ are\ rate\ constants$$

$$[Cu(\mathbf{1})_2] = K_1[(\mathbf{1})]^2[Cu]$$

$$[Cu_2(\mathbf{1})_2] = \frac{K_2[Cu(\mathbf{1})_2]^2}{[(\mathbf{1})]^2} = \frac{K_2K_1^2[(\mathbf{1})]^4[Cu]^2}{[(\mathbf{1})]^2} = K_2K_1^2[(\mathbf{1})]^2[Cu]^2$$

The steady-state approximation can be made for the change of [**A**] over time

$$\frac{d[\boldsymbol{A}]}{dt} = k_e[(\mathbf{1})] - k_{-e}[\boldsymbol{A}] - k_3[\boldsymbol{A}][Cu(\mathbf{1})_2] - k_4[\boldsymbol{A}][Cu_2(\mathbf{1})_2] = 0$$

$$[\boldsymbol{A}] = \frac{k_e[(\mathbf{1})]}{k_{-e} + k_3[Cu(\mathbf{1})_2] + k_4[Cu_2(\mathbf{1})_2]}$$

$$[\boldsymbol{A}] = \frac{k_e[(\mathbf{1})]}{k_{-e} + k_3K_1[(\mathbf{1})]^2[Cu] + k_4K_2K_1^2[(\mathbf{1})]^2[Cu]^2}$$

The change over time of [Cu(**1**)$_2$**A**]:

$$\frac{d[Cu(\mathbf{1})_2\boldsymbol{A}]}{dt} = k_3[\boldsymbol{A}][Cu(\mathbf{1})_2] - k_m[Cu(\mathbf{1})_2\boldsymbol{A}][O_2]_{DMSO}$$

To solve this differential equation we set [Cu(**1**)$_2$**A**]=x , k_3[**A**][Cu(**1**)$_2$]=a , $k_m[O_2]_{DMSO}$=b

$$\frac{dx}{dt} = a - bx$$

$$\frac{dx}{a - bx} = dt$$

$$a - bx = u \Leftrightarrow x = -\frac{u}{b} + \frac{a}{b}$$

$$dx = -\frac{du}{b}$$

$$-\int \frac{du}{bu} = \int dt$$

$$-\frac{1}{b} ln|u| = t + c \quad with\ c = -\frac{ln|u_{t=0}|}{b}$$

$$ln|u| = -bt - bc$$

$$u = e^{-bt-bc}$$

$$a - bx = e^{-bt-bc} = e^{-bc}e^{-bt} = K_c e^{-bt} \;\; With\, K_c = e^{-bc}$$

$$k_3[\boldsymbol{A}][Cu(\mathbf{1})_2] - k_m[O_2]_{DMSO}[Cu(\mathbf{1})_2\boldsymbol{A}] = K_c e^{-k_m[O_2]_{DMSO}t}$$

At t=0 : [Cu(**1**)$_2$**A**]=0, the equilibrium between **1** and **A** has already been set at t=0.

$$k_3[\boldsymbol{A}][Cu(\mathbf{1})_2] = K_c$$

$$k_3[\boldsymbol{A}][Cu(\mathbf{1})_2] - k_m[O_2]_{DMSO}[Cu(\mathbf{1})_2\boldsymbol{A}] = k_3[\boldsymbol{A}][Cu(\mathbf{1})_2]e^{-k_m[O_2]_{DMSO}t}$$

$$[Cu(\mathbf{1})_2\boldsymbol{A}] = \frac{k_3[\boldsymbol{A}][Cu(\mathbf{1})_2] - k_3[\boldsymbol{A}][Cu(\mathbf{1})_2]e^{-k_m[O_2]_{DMSO}t}}{k_m[O_2]_{DMSO}}$$

$$[Cu(\mathbf{1})_2\boldsymbol{A}] = \frac{k_3[\boldsymbol{A}][Cu(\mathbf{1})_2]}{k_m[O_2]_{DMSO}}\left(1 - e^{-k_m[O_2]_{DMSO}t}\right)$$

When t is small (in the beginning of the reaction) we can approximate by using a Maclaurin expansion:

$$e^{-k_m[O_2]_{DMSO}t} \approx 1 - k_m[O_2]_{DMSO}$$

$$[Cu(\mathbf{1})_2\boldsymbol{A}] \approx \frac{k_3[\boldsymbol{A}][Cu(\mathbf{1})_2]}{k_m[O_2]_{DMSO}}\left(1 - (1 - k_m[O_2]_{DMSO})\right)$$

$$[Cu(\mathbf{1})_2\boldsymbol{A}] \approx k_3[\boldsymbol{A}][Cu(\mathbf{1})_2]$$

The change over time of Cu$_2$(**1**)$_2$**A** is equal to:

$$\frac{d[Cu_2(\mathbf{1})_2\boldsymbol{A}]}{dt} = k_4[\boldsymbol{A}][Cu_2(\mathbf{1})_2] - k_d[Cu_2(\mathbf{1})_2\boldsymbol{A}][O_2]_{DMSO}$$

In the same manner we can derive for Cu$_2$(**1**)$_2$**A**:

$$[Cu_2(\mathbf{1})_2\boldsymbol{A}] \approx k_4[\boldsymbol{A}][Cu_2(\mathbf{1})_2]$$

The rate of formation of **2** equals:

$$\frac{d[\mathbf{2}]}{dt} = k_m[Cu(\mathbf{1})_2\boldsymbol{A}][O_2]_{DMSO} + k_d[Cu_2(\mathbf{1})_2\boldsymbol{A}][O_2]_{DMSO}$$

$$\frac{d[\mathbf{2}]}{dt} \approx k_m k_3[\boldsymbol{A}][Cu(\mathbf{1})_2][O_2]_{DMSO} + k_d k_4[\boldsymbol{A}][Cu_2(\mathbf{1})_2][O_2]_{DMSO}$$

$$\frac{d[\mathbf{2}]}{dt} \approx \frac{k_e k_m k_3 K_1[(\mathbf{1})]^3[Cu][O_2]_{DMSO} + k_e k_d k_4 K_2 K_1^2[(\mathbf{1})]^3[Cu]^2[O_2]_{DMSO}}{k_{-e} + k_3 K_1[(\mathbf{1})]^2[Cu] + k_4 K_2 K_1^2[(\mathbf{1})]^2[Cu]^2}$$

Since [(**1**)] and [O$_2$]$_{\text{DMSO}}$ can be considered constant at the start of the reaction (O$_2$ mass transfer limitation is only occurring at the maximum of the curve) The following constants can then be defined:

$$c_1 = k_e k_3 K_1 [(\mathbf{1})]^3 [O_2]_{DMSO}$$

$$c_2 = k_e k_4 K_2 K_1^2 [(\mathbf{1})]^3 [O_2]_{DMSO}$$

$$c_3 = k_{-e}$$

$$c_4 = k_3 K_1 [(\mathbf{1})]^2$$

$$c_5 = k_4 K_2 K_1^2 [(\mathbf{1})]^2$$

$$\frac{d[\mathbf{2}]}{dt} \approx \frac{k_m c_1 [Cu] + k_d c_2 [Cu]^2}{c_3 + c_4 [Cu] + c_5 [Cu]^2}$$

From this equation one can see that at high [Cu] the initial rate approaches a constant value ($k_d c_2/c_5$). Alternatively when the dimer would be inactive (k_d=0) high [Cu] loadings would result in complete inhibition of the reaction which is not experimentally observed.

VII. Synthetic experimental procedures and NMR data

The following section contains the compounds never before synthesized by this oxidation protocol. Characterization data of the remaining compounds (**2**,**7**) can be found in our communication.[S7]

1. General cross-coupling procedure:

A flame dried 100 mL flask under argon containing tetrakis(triphenylphosphine)palladium(0) (0.289 g, 0.250 mmol), substrate (5 mmol) and dry THF (30 ml) was stirred until a clear solution was seen. Next benzylzinc bromide (13 mL, 0.5 M in THF, 6.50 mmol) was added via a syringe. The mixture was stirred at 70 °C for 18 hours. After completion of the reaction the mixture was quenched by the addition of 10 % aqueous ammonium chloride (75 mL) and allowed to stir for 30 minutes. The mixture was extracted with ethyl acetate (150 mL), washed with brine (3 x 75 mL) and dried over $MgSO_4$. After filtration the solvent was removed under reduced pressure to yield a liquid which was purified by flash chromatography with an automated chromatography system using a Silica Flash Cartridge applying a heptane-ethyl acetate gradient (from 100 % heptane to 100 % ethyl acetate in 25 minutes, 25 mL/min).

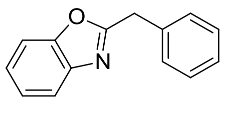

2-Benzylbenzoxazole (**22**): The general cross-coupling procedure was followed using 2-chlorobenzoxazole (1.23 g, 8 mmol). 2-Benzylbenzoxazole (**22**) was isolated as a yellow oil in 89 % yield (1.50 g).

^{1}H-NMR ($CDCl_3$, 400 MHz) δ_H: 7.69 (m, 1H), 7.45 (m, 1H), 7.36 (m, 4H), 7.28 (m, 3H), 4.27 (s, 2H).

^{13}C-NMR ($CDCl_3$, 100 MHz) δ_C: 165.2, 151.1, 141.4, 134.8, 129.0, 128.8, 127.3, 124.7, 124.2, 119.9, 110.5, 35.3.
HRMS (ESI) for $C_{14}H_{12}NO[M+H]^+$ calcd 210.0913, found 210.0911.[S8]

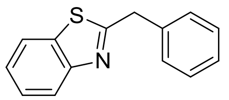

2-Benzylbenzothiazole (**24**): The general cross-coupling procedure was followed using 2-chlorobenzothiazole (1.70 g, 10 mmol). 2-Benzylbenzothiazole (**24**) was isolated as a yellow oil in 85 % yield (1.92 g).

^{1}H-NMR ($CDCl_3$, 400 MHz) δ_H: 8.00 (d, 1H, *J* = 8.1 Hz), 7.78 (d, 1H, *J* = 8.1 Hz), 7.44 (dt, 1H, *J* = 7.3, 1.2 Hz), 7.39-7.24 (m, 6H), 4.44 (s, 2H).
^{13}C-NMR ($CDCl_3$, 100 MHz) δ_C: 171.1, 153.3, 137.2, 135.7, 129.2, 128.9, 127.3, 126.0, 124.8, 122.8, 121.5, 40.7.
HRMS (ESI) for $C_{14}H_{12}NS[M+H]^+$ calcd 226.0685, found 226.0690.[S8]

2. Synthesis of 2-benzyl-1-methyl-1*H*-imidazole (18):

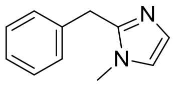

2-Benzyl-1-methyl-1*H*-imidazole (**18**): To a mixture of 2-benzyl-1*H*-imidazole (0.60 g, 3.79 mmol)) and Cs_2CO_3 (2.47 g, 7.59 mmol) in 20 mL of acetone was added methyl iodide (0.31 ml, 4.93 mmol). The reaction mixture was stirred at room temperature for 4 h, filtered through Celite® and concentrated in vacuo. Purification was achieved by using flash chromatography with an automated chromatography system using a Silica Flash Cartridge applying a heptane-ethyl acetate gradient (from 100 % heptane to 100 % ethyl acetate in 25 minutes, 25 mL/min). 2-Benzyl-1-methyl-1*H*-imidazole (**18**) was isolated as a yellow oil in 58 % yield (0.38 g).

^{1}H-NMR ($CDCl_3$, 400 MHz) δ_H: 7.32-7.27 (m, 2H), 7.25-7.15 (m, 3H), 6.97 (d, 1H, *J* = 1.2 Hz), 6.80 (d, 1H, *J* = 1.2 Hz), 4.11 (s, 2H), 3.44 (s, 3H).
^{13}C-NMR ($CDCl_3$, 100 MHz) δ_C: 146.8, 137.3, 128.8, 128.4, 127.4, 126.7, 121.1, 33.7, 33.0.
HRMS (ESI) for $C_{11}H_{12}N_2[M+H]^+$ calcd 173.1073, found 173.1075.

3. Synthesis of 2-{[2-(prop-2-en-1-yl)phenyl]methyl}pyridine (26):

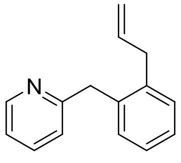

2-{[2-(prop-2-en-1-yl)phenyl]methyl}pyridine **(26)**: A flame dried roundbottom flask was charged with Tricyclohexylphosphine (0,168 g, 0,600 mmol), $Pd_2(dba)_3$ (0,275 g, 0,300 mmol), Cesium fluoride (2,005 g, 13,20 mmol) and 6 mL of Dioxane. Next Allyltributyltin (2,046 ml, 6,60 mmol) and 2-(2-chlorobenzyl)pyridine (1,222 g, 6 mmol) were added via syringe. The reaction was stirred under Argon atmosphere for 24 hours at 100 °C. After the reaction was complete the mixture was diluted with saturated aqueous NH_4Cl (20 mL) and extracted with diethylether (3 x 20 mL). The organic fractions were combined and dried on $MgSO_4$. After evaporation the product is purified further by column chromatography. 2-{[2-(prop-2-en-1-yl)phenyl]methyl}pyridine **(26)** was isolated as a yellow oil in 56 % yield. The product also contained (E)- and (Z)-2-(2-(prop-1-en-1-

yl)benzyl)pyridine which were inseparable from the desired 2-{[2-(prop-2-en-1-yl)phenyl]methyl}pyridine **(26)**.

^{1}H-NMR ($CDCl_3$, 400 MHz) δ_H: 8.55 (d, 1H, *J* = 4.8 Hz), 7.52 (dt, 1H, *J* = 7.7, 1.7 Hz), 7.24-7.14 (m, 3H), 7.12-7.04 (m, 2H), 6.95 (d, 1H, *J* = 7.9 Hz), 5.93-5.81 (m, 1H), 5.01 (dd, 1H, *J* = 10.1, 1.4 Hz), 4.95 (dd, 1H, *J* = 17.0, 1.6 Hz), 4.20 (s, 2H), 2.37 (d, 2H, *J* = 6.3 Hz).
HRMS (ESI) for $C_{11}H_{12}N_2[M+H]^+$ calcd 210.1277, found 210.1283.

4. General oxidation procedure:

Two 10 mL microwave vials were subsequently charged with CuI (0.0095 g, 0.05 mmol, 10 mol%), substrate (0.5 mmol), solvent (1 mL) and acetic acid (0.030 g, 0.5 mmol). The vials were flushed with O_2 for 1 min, capped with an aluminum crimp cap/septum and finally stirred at 100 °C for 24 h in an oil bath with a balloon filled with O_2 through the septum. After cooling down to room temperature, the content of the vials was transferred into a separation funnel and the vials were rinsed with dichloromethane (20 mL). Sodium bicarbonate solution (10 mL, sat.) was added and the organic phase was separated. The water phase was extracted twice with dichloromethane (10 mL). The combined organic fractions were washed with brine (20 mL), dried over $MgSO_4$ and filtered over a pad of Celite®. The solvent was removed under reduced pressure and the resulting residue was purified via column chromatography with an automated chromatography system using a Silica Flash Cartridge applying a heptane-ethyl acetate gradient (from 100 % heptane to 100 % ethyl acetate in 25 minutes, 25 mL/min).
For ^{1}H-NMR screening of the reaction mixture with an internal standard, a known quantity of 1,3,5-trimethoxybenzene was added to the mixture obtained after work-up but before chromatography and everything was carefully dissolved in $CDCl_3$. ^{1}H-NMR is then used to calculate the yield and conversion of the reaction.
Alternative work-up procedure: After the reaction is completed and the vials have cooled to room temperature they can be rinsed with dichloromethane (20 mL) and filtered over Celite®. The solvent can then be removed under reduced pressure and the residue can be further purified by column chromatograpy. This alternative work-up was used in the purification of compound **2** explaining the discrepancy between the earlier reported yield.[S7]

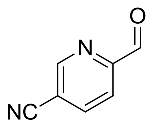

6-Formylnicotinonitrile (**13**): The general oxidation procedure was followed using 5-cyano-2-methylpyridine (**12**) (0.059 g, 0.5 mmol). 6-Formylnicotinonitrile (**13**) was isolated as a yellow solid in 27 % yield (17 mg) (60 % (38 mg) at 120 °C reaction temperature).

^{1}H-NMR ($CDCl_3$, 400 MHz) δ_H: 10.12 (d, 1H, *J* = 0.7 Hz), 9.06 (dd, 1H, *J* = 1.7, 0.7 Hz), 8.18 (ddd, 1H, *J* = 8.1, 1.9, 0.7), 8.07 (dd, 1H, *J* = 8.0, 0.8).
^{13}C-NMR ($CDCl_3$, 100 MHz) δ_C: 191.8, 154.2, 152.8, 140.8, 121.1, 115.8, 113.7.
HRMS (ESI) for $C_7H_5N_2O[M+H]^+$ calcd 133.0396, found 133.0402. Melting point: 98 °C.

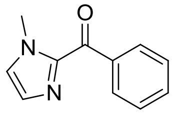

(1-Methyl-1*H*-imidazol-2-yl)(phenyl)methanone (**19**): The general oxidation procedure was followed using 2-benzyl-1-methyl-1*H*-imidazole (**18**) (0.086 g, 0.5 mmol). (1-Methyl-1*H*-imidazol-2-yl)(phenyl)methanone (**19**) was isolated as a yellow liquid in 34 % yield (32 mg) (85 % (80 mg) at 120 °C reaction temperature).

^{1}H-NMR ($CDCl_3$, 400 MHz) δ_H: 8.28 (d, 2H, *J* = 7.7 Hz), 7.56 (t, 1H, *J* = 7.4 Hz), 7.48 (t, 2H, *J* = 7.4 Hz), 7.23 (bs, 1H), 7.10 (bs, 1H), 4.07 (s, 3H).
^{13}C-NMR ($CDCl_3$, 100 MHz) δ_C: 184.2, 143.2 (very small), 137.4, 132.7, 130.8, 129.3, 128.1, 126.8, 36.4.
HRMS (ESI) for $C_{11}H_{11}N_2O[M+H]^+$ calcd 187.0866, found 187.0866.
The small broad peak in the ^{13}C-NMR spectrum at 143.2 ppm is the C(2) quaternary carbon of the imidazole moiety and is known to have a very long relaxation time.[S9]

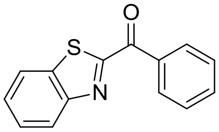

Benzothiazol-2-yl(phenyl)methanone (**25**): The general oxidation procedure was followed using 2-benzylbenzothiazole (**24**) (0.113 g, 0.5 mmol). Benzothiazol-2-yl(phenyl)methanone (**25**) was isolated as a white solid in 99 % yield (119 mg).

^{1}H-NMR ($CDCl_3$, 400 MHz) δ_H: 8.56 (m, 2H), 8.23 (m, 1H), 8.00 (m, 1H), 7.66 (m, 1H), 7.60-7.48 (m, 4H).
^{13}C-NMR ($CDCl_3$, 100 MHz) δ_C: 185.3, 167.2, 153.9, 137.1, 135.0, 133.9, 131.3, 128.5, 127.6, 126.9, 125.8, 122.2
HRMS (ESI) for $C_{14}H_{10}NOS[M+H]^+$ calcd 240.0478, found 240.0492. Melting point: 102 °C.[S10]

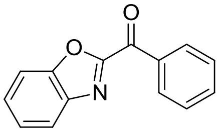

Benzoxazol-2-yl(phenyl)methanone (**23**): The general oxidation procedure was followed using 2-benzylbenzoxazole (**22**) (0.105 g, 0.5 mmol). Benzoxazol-2-yl(phenyl)methanone (**23**) was isolated as a white solid in 99 % yield (110 mg).

^{1}H-NMR ($CDCl_3$, 400 MHz) δ_H: 8.55 (m, 2H), 7.94 (d, 1H, *J* = 7.9 Hz), 7.72-7.64 (m, 2H), 7.59-7.51 (m, 3H), 7.46 (m, 1H).
^{13}C-NMR ($CDCl_3$, 100 MHz) δ_C: 180.5, 157.2, 150.4, 140.8, 135.1, 134.3, 131.0, 128.6, 128.4, 125.7, 122.4, 111.9.
HRMS (ESI) for $C_{14}H_{10}NO_2[M+H]^+$ calcd 224.0706, found 224.0713. Melting point: 74 °C.[S10]

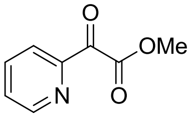

Methyl oxo(pyridin-2-yl)acetate (**15**): The general oxidation procedure was followed using methyl 2-(pyridin-2-yl)acetate (**14**) (0.076 g, 0.5 mmol). Methyl oxo(pyridin-2-yl)acetate (**15**) was isolated as a yellow oil in 23 % yield (19 mg) (83 % (69 mg) using *n*-BuOAc as the solvent and with the addition of molecular sieves).

^{1}H-NMR ($CDCl_3$, 400 MHz) δ_H: 8.75 (ddd, 1H, *J* = 4.8, 1.5, 0.9 Hz), 8.11 (dt, 1H, *J* = 7.8, 1.0 Hz), 7.91 (td, 1H, *J* = 7.7, 1.6 Hz), 7.55 (ddd, 1H, *J* = 7.6, 4.8, 1.2 Hz), 4.01 (s, 3H).
^{13}C-NMR ($CDCl_3$, 100 MHz) δ_C: 187.5, 165.6, 150.3, 149.8, 137.2, 128.3, 123.4, 52.6.
HRMS (ESI) for $C_8H_8NO_3$ $[M+H]^+$, calcd 166.0499, found 166.0503.

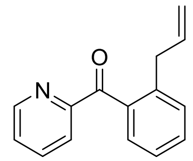

[2-(prop-2-en-1-yl)phenyl](pyridin-2-yl)methanone (**27**): The general oxidation procedure was followed using 2-{[2-(prop-2-en-1-yl)phenyl]methyl}pyridine (**26**) (0.105 g, 0.5 mmol) and a reaction time of 48 hours. [2-(prop-2-en-1-yl)phenyl](pyridin-2-yl)methanone (**27**) was isolated as a brown oil in 54 % yield (0.035 mg). Note: The starting material also contained (E)- and (Z)-2-[2-(prop-1-en-1-yl)benzyl]pyridine which were inseparable from 2-{[2-(prop-2-en-1-yl)phenyl]methyl}pyridine (**26**). However this does not influence the experiment as 5-endo-trig radical cyclization on (E)- or (Z)-2-(2-(prop-1-en-1-yl)benzyl)pyridine also leads to the same cyclic skeleton (2-(2-methyl-2,3-dihydro-1*H*-inden-1-yl)pyridine) and 4-exo-trig radical cyclization is disfavored and reversible.[S13] During purification of **27** a fraction containing a mixture of (E)- and (Z)-[2-(2-(prop-1-en-1-yl)phenyl](pyridin-2-yl)methanone was also isolated.

^{1}H-NMR ($CDCl_3$, 400 MHz) δ_H: 8.69 (ddd, 1H, *J* = 0.8, 1.5, 4.7 Hz), 8.09 (d, 1H, *J* = 7.8 Hz), 7.88 (dt, 1H, *J* = 1.7, 7.6 Hz), 7.50-7.40 (m, 3H), 7.36-7.24 (m, 2H), 5.99-5.86 (m, 1H), 4.99 (m, 1H), 4.97-4.93 (m, 1H), 3.48 (d, 2H, *J* = 6.6 Hz).
^{13}C-NMR ($CDCl_3$, 100 MHz) δ_C: 197.3, 154.9, 149.2, 139.7, 137.6, 137.1, 136.9, 131.0, 130.4, 130.0, 126.5, 125.6, 124.2, 116.1, 37.9.
^{13}C-APT-NMR ($CDCl_3$, 100 MHz) δ_C: 197.3 (↓), 154.9 (↓), 149.2 (↑), 139.7 (↓), 137.6 (↓), 137.1 (↑), 136.9 (↑), 131.0 (↑), 130.4 (↑), 130.0 (↑), 126.5 (↑), 125.6 (↑), 124.2 (↑), 116.1 (↓), 37.9 (↓).
HRMS (ESI) for $C_{15}H_{14}NO[M+H]^+$ calcd 224.1070, found 224.1075.

VIII. DFT calculation of equilibrium constants and bond dissociation energies

a) Equilibrium constants

To calculate the desired equilibrium constants, the geometries of the different conformations of both the imine- and the enamine-form were optimized at the B3PW91/aug-cc-pVDZ level of theory. Due to the rather large dipole moment of the molecules and the difference in dipole moment of the imine and the respective enamine tautomer (table S5, the self-consistent reaction field (scrf) model was used to account for solvent-solute interactions with DMSO. The Gibbs free energies of all conformers were calculated and the most stable imine and respective enamine conformer were used to calculate the equilibrium constant K_{eq} and pK_{eq} as follows:

$$\Delta G° = G°_{enamine} - G°_{imine}$$

$$K_{eq} = \exp\left(-\frac{\Delta G°}{RT}\right)$$

$$pK_{eq} = -\log K_{eq}$$

with the temperature T=298.15 K and ideal gas constant R= 8.31 $Jmol^{-1}K^{-1}$. For all calculations the Gaussian09 program was used.[S11]

Compounds **28**-**35** are not included in the manuscript text since they have been previously published.[S7] Interesting to note however is the case of compound **34** which has two benzylic positions viable for oxidation. Tautomerization from the double benzylic position provides a pK_{eq} value of 11.7 similar to 2-benzylpyridine (**1**). Tautomerization from the 6-methyl in **34** provides a pK_{eq} of 15.6 which is similar to 2-methylpyridine (**10**). However when the double benzylic position of **34** is oxidized this creates an electron withdrawing effect causing the pK_{eq} value of the 6-methyl to decrease to 14.7 (see **35**) making it viable for oxidation as exemplified in the original communication.

Table S4 Calculated K_{eq} and pK_{eq}

Compound	Substrate	K_{eq}	pK_{eq}
**1**[a]	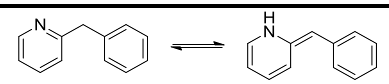	3.4E-12	11.5
**4**[a]	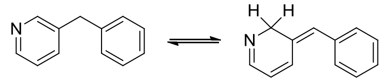	4.0E-21	20.4
**6**[a]	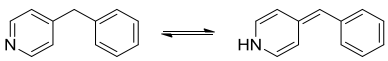	9.6E-12	11.0
**8**[a]	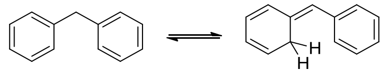	2.2E-22	21.7
**10**[a]	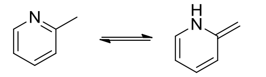	1.4E-16	15.9
**12**	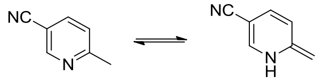	1.13E-13	12.95
**14**	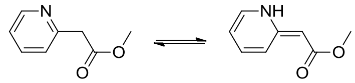	6.99E-07	6.2
**16**	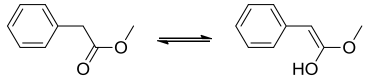	1.53E-19	18.8
**18**	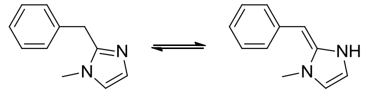	1.25E-14	13.90
**20**	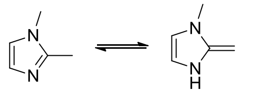	1.11E-19	19.0
**22**	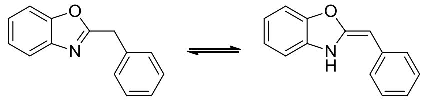	8.02E-10	9.1
**24**	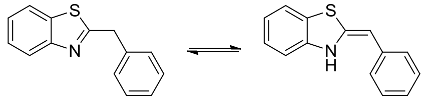	7.90E-09	8.1
**28**[a]	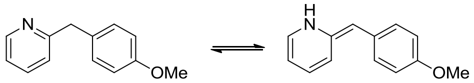	4.0E-13	12.4
**29**[a]	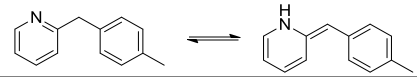	9.8E-13	12.0

**30**[a]	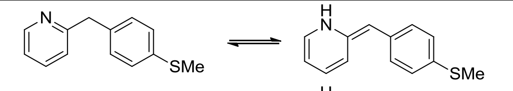	2.5E-12	11.7
**31**[a]	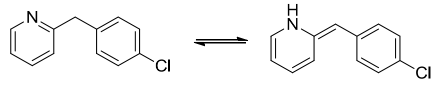	1.0E-11	11.0
**32**[a]	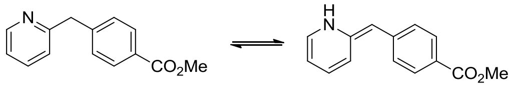	2.3E-10	9.6
**33**[a]	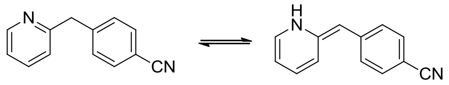	3.6E-10	9.4
**34a**[a]	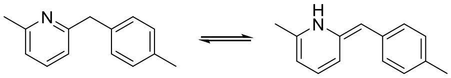	1.96E-12	11.7
**34b**[a]	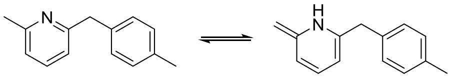	2.44E-16	15.6
**35**[a]	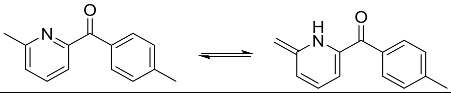	2.13E-15	14.7

a) The oxidation reaction of these compounds was previously reported.[S7]

Table S5 Calculated thermodynamical values and dipole moments

Compound	ΔH (kJ mol^{-1})	ΔS (kJ mol^{-1})	ΔG (kJ mol^{-1})	μ_{imine} (D)	$\mu_{enamine}$ (D)
**1**[a]	64.6	-0.014	68.7	1.9	2.2
**4**[a]	109.9	-0.022	116.4	3.3	4.6
**6**[a]	66.8	-0.019	72.5	2.7	4.8
**8**[a]	114.6	-0.030	123.6	0.4	0.8
**10**[a]	92.8	-0.008	95.3	1.9	1.9
**12**	70.3	-0.012	73.9	6.0	6.4
**14**	56.1	-0.014	60.4	2.4	3.0
**16**	103.4	-0.013	107.3	2.5	3.6
**18**	72.8	-0.022	79.3	5.3	6.4
**20**	107.7	-0.001	108.1	5.5	3.0
**22**	46.5	-0.018	51.9	1.8	5.0
**24**	40.9	-0.018	46.2	1.6	4.2

**28**[a]	67.4	-0.013	71.4	2.6	1.0
**29**[a]	65.3	-0.021	71.5	1.8	1.8
**30**[a]	66.4	-0.008	68.8	1.7	3.2
**31**[a]	66.2	-0.014	70.3	3.0	3.2
**32**[a]	57.9	-0.016	62.8	2.7	3.8
**33**[a]	57.3	-0.017	62.4	6.4	8.1
**34a**[a]	60.3	-0.022	66.8	2.3	3.8
**34b**[a]	85.4	-0.012	89.1	2.3	3.6
**35**[a]	78.9	-0.016	83.7	4.6	5.7

a) The oxidation reaction of these compounds was previously reported.[S7]

b) Bond dissociation energies

Bond dissociation enthalpies (BDE) for the different substrates were calculated for the homolytic cleavage of a methylene hydrogen.

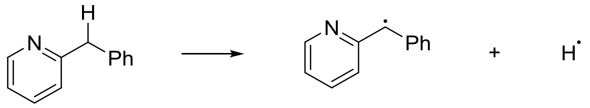

In analogy to the BDEs calculated by Van Speybroeck et al. for polyaromatic hydrocarbons, the BDE at 298K for each substrate was calculated as the difference of the enthalpies of formation $\Delta_f H_{298}$ of the products and the reactant, using equation (1)

$$BDE_{298}(R-H) = [\Delta_f H_{298}(R^\cdot) + \Delta_f H_{298}(H^\cdot)] - \Delta_f H_{298}(R-H) \qquad (1)$$

The enthalpy of formation of the lowest energy conformers were used.[S12] All calculations are performed using the Gaussian G09 rev D.01 program, using the B3PW91/aug-cc-pVDZ level of theory with the scrf model to take solvent-solute interactions with DMSO into account.[S11]

Table S6 Calculated Bond Dissociation Energies (BDE)

Entry	Substrate	BDE (kJ/mol)
1	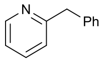 **1**	320
2	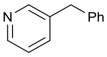 **4**	325
3	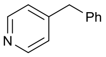 **6**	328
4	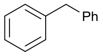 **8**	326
5	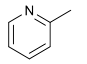 **10**	363
6	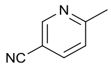 **12**	361
7	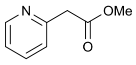 **14**	342
8	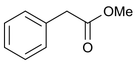 **16**	334
9	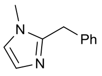 **18**	311
10	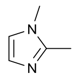 **20**	354
11	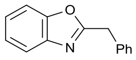 **22**	314
12	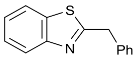 **24**	316

No correlation between the homolytical BDE of the substrates in Table S6 and their reactivity could be found. For instance, while 2- and 4-benzylpyridine (**1** and **6**) are reactive, diphenylmethane (**8**) and 3-

benzylpyridine (**4**) are not. Their bond dissociation energies are however within a margin of error similar (see Table S6 entries 1-4). Furthermore when we tried to oxidize diphenylmethane (**8**) under our standard conditions in the presence of a pyridine resembling 2-benzylpyridine, which can act as a ligand, no oxidation product was seen and the starting material was recovered in 87 % (Figure S28).

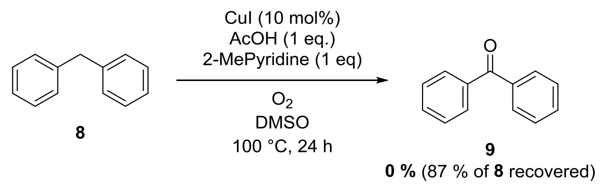

Figure S28: *Oxidation of diphenylmethane (8) under our standard conditions with the addition of 2-methylpyridine to act as an N-coordinating ligand. Reaction conditions:* ***8*** *(0.5 mmol), CuI (10 mol %), AcOH (1 eq.), 2-methylpyridine (1 eq.),* O_2 *(balloon), DMSO (10 mL), 100 °C.*

Compound 1 imine

Electronic energy: -518.519559761 Hartree

C	-1.28017	-0.13212	0.64690
C	-1.81179	-1.25275	-0.00563
C	-2.94861	-1.10449	-0.79548
C	-3.52329	0.16143	-0.91264
C	-2.92778	1.21717	-0.22467
H	-1.33766	-2.22772	0.10908
H	-3.38164	-1.96361	-1.30972
H	-4.41423	0.32941	-1.51729
H	-3.35272	2.22227	-0.28714
N	-1.83543	1.08529	0.53980
C	-0.02734	-0.24499	1.49010
H	-0.01337	-1.22176	1.99124
H	-0.06624	0.53360	2.26293
C	1.23575	-0.08656	0.66573
C	1.99467	-1.20262	0.29042
C	1.66039	1.18313	0.24838
C	3.14698	-1.05731	-0.48695
H	1.68450	-2.19843	0.61281
C	2.81104	1.33242	-0.52748
H	1.08296	2.06331	0.53521
C	3.55881	0.21141	-0.89960
H	3.72643	-1.93868	-0.76651
H	3.12833	2.32869	-0.83986
H	4.45962	0.32761	-1.50385

Compound 1 enamine

Electronic energy: -518.496512529 Hartree

C	1.27306	-0.50020	0.07645
C	2.74056	1.41714	-0.29114
C	3.87280	0.67476	-0.14799
C	3.71983	-0.72941	0.10068

H	0.70340	1.43829	-0.36598
H	2.74657	2.48585	-0.49697
H	4.85129	1.14190	-0.22687
H	4.60543	-1.35724	0.20804
C	0.01763	-1.06455	0.16937
H	0.02251	-2.15216	0.25231
C	-1.29578	-0.45147	0.09615
C	-1.58236	0.90928	0.36902
C	-2.40434	-1.27342	-0.23189
C	-2.88093	1.41417	0.28033
H	-0.80475	1.58486	0.72645
C	-3.69719	-0.76823	-0.31636
H	-2.22969	-2.33310	-0.42927
C	-3.94973	0.58755	-0.07148
H	-3.05812	2.46740	0.50566
H	-4.51903	-1.43757	-0.57805
H	-4.96211	0.98679	-0.14156
C	2.47958	-1.28669	0.20163
H	2.36232	-2.35406	0.38635
N	1.50335	0.85080	-0.17838

Compound 1 radical

Electronic energy: -517.883590590 Hartree

C	1.32582	-0.42379	0.00001
C	2.45538	-1.28496	-0.00000
C	3.73345	-0.74753	0.00001
C	3.87757	0.64312	0.00003
C	2.71514	1.42241	0.00004
H	2.30757	-2.36503	-0.00002
H	4.60782	-1.39947	0.00001
H	4.85867	1.11689	0.00004
H	2.79541	2.51323	0.00005
N	1.47927	0.92896	0.00003
C	0.02065	-1.01736	-0.00001
H	0.02669	-2.10929	-0.00002
C	-1.28210	-0.42237	-0.00001
C	-2.40531	-1.29541	-0.00002
C	-1.53960	0.97555	-0.00001
C	-3.70455	-0.80559	-0.00002
H	-2.23500	-2.37354	-0.00001
C	-2.84474	1.45571	-0.00002
H	-0.69592	1.66093	-0.00001
C	-3.93515	0.57641	-0.00002
H	-4.54577	-1.50024	-0.00002
H	-3.01893	2.53297	-0.00002
H	-4.95475	0.96373	-0.00003

Compound 4 imine

Electronic energy: -518.517486087 Hartree

C	1.63711	-1.01934	-0.61211

C	1.27906	-0.34101	0.56194
C	2.15208	0.65316	1.00959
C	3.31036	0.92802	0.28214
C	3.56157	0.19184	-0.87388
H	0.98809	-1.81122	-0.99693
H	1.92996	1.20834	1.92274
H	4.01005	1.69831	0.60678
H	4.46002	0.38085	-1.46623
N	2.74366	-0.77243	-1.32091
C	0.00161	-0.68481	1.29317
H	-0.04505	-1.77068	1.45494
H	0.02789	-0.21971	2.28970
C	-1.25766	-0.24583	0.56891
C	-2.29045	-1.15684	0.31666
C	-1.42562	1.08512	0.15933
C	-3.46375	-0.75250	-0.32704
H	-2.17684	-2.19731	0.62721
C	-2.59530	1.49351	-0.48254
H	-0.63221	1.81200	0.34206
C	-3.62050	0.57503	-0.72853
H	-4.25612	-1.47871	-0.51513
H	-2.70820	2.53364	-0.79251
H	-4.53431	0.89351	-1.23205

Compound 4 enamine

Electronic energy: -518.475033248 Hartree

C	-2.39381	-1.29759	-0.00000
C	-1.25962	-0.38265	-0.00000
C	-3.03954	1.37935	0.00000
C	-3.95648	0.39847	0.00000
H	-2.15887	-2.36881	-0.00001
H	-3.35683	2.42306	0.00000
H	-5.02375	0.62540	0.00000
C	-0.02295	-0.95949	-0.00000
C	-1.56883	1.09488	0.00000
H	-1.10485	1.58441	-0.87441
H	-1.10485	1.58441	0.87441
H	-0.02493	-2.05366	-0.00000
C	1.31710	-0.38768	0.00000
C	2.40223	-1.29411	0.00000
C	1.61952	0.99225	0.00000
C	3.72094	-0.85208	0.00000
H	2.19461	-2.36548	0.00000
C	2.94269	1.43271	0.00000
H	0.82973	1.73654	0.00000
C	3.99864	0.51855	0.00000
H	4.53603	-1.57688	0.00000
H	3.15027	2.50339	0.00000
H	5.03076	0.87100	0.00000
N	-3.64667	-0.97415	-0.00000

Compound 4 radical

Electronic energy: -517.879268697 Hartree

C	2.43490	-1.22116	-0.26768
C	1.30793	-0.39988	0.01581
C	2.91713	1.38014	0.34935
C	3.93435	0.48074	0.01990
H	2.26807	-2.27598	-0.50546
H	3.16371	2.40873	0.61311
H	4.97797	0.80186	0.00610
C	0.01301	-1.01048	-0.01224
H	0.02483	-2.10335	-0.02546
C	-1.29498	-0.42099	-0.02202
C	-2.40688	-1.26200	0.25478
C	-1.57033	0.93537	-0.33778
C	-3.70555	-0.76994	0.26038
H	-2.22714	-2.31505	0.47799
C	-2.87438	1.41944	-0.33872
H	-0.76377	1.60427	-0.63043
C	-3.94993	0.57851	-0.02978
H	-4.53652	-1.43908	0.48810
H	-3.05785	2.46386	-0.59498
H	-4.96909	0.96641	-0.02940
N	3.69821	-0.80954	-0.27612
C	1.59869	0.94471	0.35405
H	0.80852	1.62840	0.65704

Compound 6 imine

Electronic energy: -518.518788729 Hartree

C	-1.34408	0.94716	-0.26375
C	-1.28857	-0.44781	-0.35848
C	-2.45577	-1.14920	-0.03756
C	-3.59630	-0.44561	0.34780
C	-2.53326	1.55920	0.12930
H	-0.47082	1.55931	-0.48922
H	-2.48158	-2.23881	-0.08318
H	-4.51043	-0.98813	0.60220
H	-2.58504	2.64852	0.20316
N	-3.65392	0.89014	0.43494
C	-0.04579	-1.17785	-0.82446
H	-0.05565	-1.19824	-1.92492
H	-0.11411	-2.22398	-0.49544
C	1.25652	-0.57595	-0.34866
C	2.08517	0.13931	-1.22162
C	1.65792	-0.72104	0.98761
C	3.28501	0.70001	-0.77362
H	1.79121	0.25781	-2.26631
C	2.85500	-0.16424	1.43903
H	1.02730	-1.27967	1.68200
C	3.67331	0.55034	0.55866

H	3.91813	1.25255	-1.46955
H	3.15322	-0.29102	2.48093
H	4.61020	0.98470	0.91023

Compound 6 enamine

Electronic energy: -518.497438236 Hartree

C	-2.41995	-1.27679	0.21207
C	-1.23444	-0.44678	0.05391
C	-1.54078	0.94496	-0.24126
C	-2.82242	1.39563	-0.31130
C	-3.67702	-0.76994	0.14408
H	-2.30206	-2.34317	0.40385
H	-0.75049	1.65841	-0.45505
H	-3.06527	2.43040	-0.54485
H	-4.82855	0.92763	-0.14719
H	-4.56865	-1.37880	0.27743
N	-3.88840	0.56473	-0.10054
C	0.01824	-1.01882	0.14410
C	1.34424	-0.42293	0.07809
C	2.43968	-1.26191	-0.24575
C	1.64978	0.92883	0.36705
C	3.74377	-0.77984	-0.31317
H	2.24851	-2.31690	-0.45382
C	2.95742	1.41030	0.29875
H	0.86596	1.60932	0.69441
C	4.01644	0.56740	-0.04973
H	4.55642	-1.46092	-0.57346
H	3.15211	2.45828	0.53507
H	5.03672	0.94930	-0.10263
H	0.02426	-2.10746	0.24904

Compound 6 radical

Electronic energy: -517.879686203 Hartree

C	-2.43539	-1.23073	0.29003
C	-1.30493	-0.43089	-0.01373
C	-1.58585	0.91071	-0.37052
C	-2.90104	1.36086	-0.37004
C	-3.70746	-0.67677	0.26690
H	-2.30779	-2.28114	0.55411
H	-0.79907	1.59005	-0.69006
H	-3.11413	2.39522	-0.65144
H	-4.57336	-1.29562	0.51521
N	-3.96369	0.60433	-0.05059
C	-0.00443	-1.03652	0.00768
C	1.29729	-0.43519	0.02206
C	2.41530	-1.26129	-0.27374
C	1.55885	0.91780	0.36302
C	3.70866	-0.75586	-0.27633
H	2.24430	-2.31170	-0.51502
C	2.85750	1.41485	0.36686

H	0.74497	1.57058	0.67137
C	3.93943	0.58962	0.03741
H	4.54577	-1.41153	-0.51963
H	3.03260	2.45576	0.64205
H	4.95465	0.98773	0.04000
H	-0.00978	-2.12921	0.00974

Compound 8 imine

Electronic energy: -502.482127873 Hartree

C	-2.60920	1.44846	-0.61489
C	-1.43983	1.09858	0.06092
C	-1.27305	-0.18932	0.59165
C	-2.30814	-1.11728	0.42481
C	-3.48162	-0.77207	-0.25277
C	-3.63664	0.51311	-0.77450
H	-2.72114	2.45614	-1.01860
H	-0.64342	1.83614	0.17573
H	-2.19594	-2.12499	0.82991
H	-4.27556	-1.51105	-0.37247
H	-4.55059	0.78582	-1.30405
C	-0.01265	-0.55854	1.35025
H	0.01851	0.00835	2.29270
H	-0.06717	-1.62143	1.62580
C	1.26720	-0.30237	0.58278
C	2.17980	0.67182	1.00486
C	1.56656	-1.04126	-0.57211
C	3.36022	0.90848	0.29339
H	1.96616	1.25421	1.90321
C	2.74367	-0.81057	-1.28436
H	0.86908	-1.80684	-0.91785
C	3.64615	0.16772	-0.85419
H	4.05789	1.67274	0.63962
H	2.96056	-1.39798	-2.17811
H	4.56678	0.34830	-1.41090

Compound 8 enamine

Electronic energy: -502.437909212 Hartree

C	3.67855	-0.90322	0.00000
C	2.39357	-1.33950	0.00000
C	1.25681	-0.43542	0.00000
C	2.99966	1.43017	0.00000
C	3.98889	0.51792	0.00000
H	4.49812	-1.62336	0.00000
H	2.18379	-2.41106	0.00000
H	3.22773	2.49771	0.00000
H	5.03371	0.83237	0.00000
C	0.00550	-0.98775	0.00000
C	1.54815	1.05205	0.00000
H	1.06402	1.52791	0.87141
H	1.06402	1.52791	-0.87141

C	-1.32717	-0.39680	0.00000
C	-2.42445	-1.29153	0.00000
C	-1.62675	0.98516	0.00000
C	-3.74024	-0.84125	0.00000
H	-2.22645	-2.36500	0.00000
C	-2.94819	1.43445	0.00000
H	-0.83584	1.72802	0.00000
C	-4.01259	0.53102	0.00000
H	-4.55886	-1.56260	0.00000
H	-3.14608	2.50734	0.00000
H	-5.04234	0.89039	0.00000
H	-0.01230	-2.08150	0.00000

Compound 8 radical
Electronic energy: -501.843876555 Hartree

C	3.71790	0.75504	-0.27829
C	2.42251	1.25607	-0.28137
C	1.30407	0.43101	0.01799
C	2.87330	-1.41247	0.37566
C	3.95455	-0.58781	0.04332
H	4.55272	1.41311	-0.52415
H	2.25010	2.30491	-0.52957
H	3.04986	-2.45167	0.65736
H	4.97101	-0.98285	0.04941
C	0.00000	1.03044	0.00000
C	1.57254	-0.91903	0.36717
H	0.76080	-1.57348	0.67806
C	-1.30407	0.43101	-0.01799
C	-2.42251	1.25607	0.28137
C	-1.57254	-0.91903	-0.36717
C	-3.71790	0.75504	0.27829
H	-2.25010	2.30491	0.52957
C	-2.87330	-1.41247	-0.37566
H	-0.76080	-1.57349	-0.67805
C	-3.95455	-0.58781	-0.04332
H	-4.55272	1.41311	0.52415
H	-3.04986	-2.45167	-0.65736
H	-4.97101	-0.98285	-0.04941
H	0.00000	2.12360	0.00000

Compound 10 imine
Electronic energy: -287.532681973 Hartree

C	0.88024	0.00260	0.00000
C	0.16267	1.20610	0.00000
C	-1.23064	1.17256	0.00000
C	-1.87161	-0.06516	0.00000
C	-1.07733	-1.21209	0.00000
H	0.69496	2.15764	0.00000
H	-1.80625	2.09939	0.00000
H	-2.95841	-0.14583	0.00000

H	-1.54357	-2.20103	0.00000
N	0.26047	-1.19134	0.00000
C	2.38266	-0.01596	0.00000
H	2.75869	-0.54896	-0.88464
H	2.79661	0.99880	-0.00001
H	2.75869	-0.54895	0.88465

Compound 10 enamine

Electronic energy: -287.499193984 Hartree

C	0.00000	1.01733	0.00000
C	1.24311	0.26538	0.00000
C	1.26065	-1.09588	0.00000
C	0.03803	-1.85148	0.00000
C	-1.13221	-1.15460	0.00000
H	-2.04292	0.67341	0.00000
H	2.17144	0.83640	0.00000
H	2.21635	-1.62295	0.00000
H	0.03315	-2.93854	0.00000
H	-2.11117	-1.63094	0.00000
N	-1.14504	0.20885	0.00000
C	-0.08248	2.37790	0.00000
H	0.82731	2.97359	0.00000
H	-1.04152	2.89518	0.00000

Compound 10 radical

Electronic energy: -286.879745381 Hartree

C	0.00000	0.95454	0.00000
C	1.21673	0.21540	0.00000
C	1.17558	-1.16998	0.00000
C	-0.06983	-1.80688	0.00000
C	-1.22345	-1.00733	0.00000
H	2.16789	0.74836	0.00000
H	2.09835	-1.75162	0.00000
H	-0.15510	-2.89307	0.00000
H	-2.20855	-1.48286	0.00000
N	-1.21464	0.32032	0.00000
C	0.00148	2.36489	0.00000
H	0.93683	2.92177	0.00000
H	-0.94003	2.91128	0.00000

Compound 12 imine

Electronic energy: -379.749285774 Hartree

C	0.34528	-1.16589	0.00000
C	1.09262	0.02382	0.00000
C	0.40235	1.24409	0.00000
C	-0.98492	1.21380	0.00000
C	-1.65276	-0.01974	0.00000
N	-0.98377	-1.18872	0.00000
H	0.94436	2.18888	0.00000
H	0.85728	-2.13036	0.00000

H	-1.55307	2.14308	0.00000
C	-3.14873	-0.10198	0.00000
H	-3.49570	-0.65470	0.88418
H	-3.60765	0.89237	-0.00001
H	-3.49570	-0.65472	-0.88416
C	2.52089	-0.02351	0.00000
N	3.68408	-0.06958	0.00000

Compound 12 enamine
Electronic energy: -379.723015065 Hartree

C	0.35094	-1.15670	0.00021
C	1.07803	0.01235	0.00023
C	0.34413	1.26332	0.00023
C	-1.01019	1.26081	0.00003
C	-1.78343	0.02892	-0.00006
H	0.89589	2.20258	0.00039
H	0.81784	-2.13940	0.00022
H	-1.56492	2.19812	-0.00013
C	2.49411	-0.02415	-0.00009
N	3.66220	-0.03004	-0.00033
N	-0.99225	-1.13804	0.00007
H	-1.47430	-2.02821	0.00005
C	-3.13649	-0.04068	-0.00024
H	-3.66315	-0.99398	-0.00019
H	-3.72362	0.87416	-0.00038

Compound 12 radical
Electronic energy: -379.097263443 Hartree

C	-0.28366	-1.17630	-0.00001
C	-1.03013	0.02748	-0.00004
C	-0.33316	1.25131	-0.00002
C	1.04588	1.21844	0.00003
C	1.72590	-0.03407	0.00007
H	-0.87411	2.19649	-0.00005
H	-0.81153	-2.13287	-0.00002
H	1.62137	2.14342	0.00005
C	-2.45332	-0.01581	-0.00010
N	-3.61807	-0.05898	-0.00014
N	1.03316	-1.21932	0.00005
C	3.12866	-0.10293	0.00013
H	3.62628	-1.07079	0.00015
H	3.73136	0.80313	0.00015

Compound 14 imine
Electronic energy: -515.352848885 Hartree

C	-1.66944	0.05460	0.03215
C	-0.61476	0.18226	-1.04757
C	0.79203	0.08479	-0.52197
N	1.29373	-1.15251	-0.38595
C	2.53838	-1.27783	0.08973

C	3.34114	-0.19522	0.44929
C	2.81776	1.08843	0.30310
C	1.52247	1.23070	-0.19059
O	-2.89295	-0.02085	-0.51148
O	-1.46348	0.03492	1.22853
C	-3.99060	-0.11634	0.41342
H	-0.79551	-0.60466	-1.79120
H	-0.78221	1.14434	-1.55178
H	2.91696	-2.29846	0.18621
H	4.34943	-0.35929	0.82872
H	3.40886	1.96611	0.56750
H	1.08076	2.21889	-0.32015
H	-4.88976	-0.16351	-0.20597
H	-3.89663	-1.02256	1.02302
H	-4.01847	0.76583	1.06361

Compound 14 enamine
Electronic energy: -515.342147571 Hartree

C	-1.75255	0.04684	-0.00002
C	-0.59271	-0.78931	-0.00001
C	0.71915	-0.32487	-0.00001
N	1.74696	-1.25063	0.00000
C	3.07054	-0.94532	0.00001
C	3.48447	0.35688	0.00001
C	2.48202	1.36585	0.00000
C	1.14949	1.04423	-0.00001
O	-2.89883	-0.70913	-0.00002
O	-1.81326	1.28020	-0.00000
C	-4.12380	0.02453	0.00003
H	-0.76284	-1.86526	-0.00001
H	1.49134	-2.23102	0.00000
H	3.75006	-1.79457	0.00001
H	4.54478	0.59631	0.00002
H	2.77846	2.41581	0.00000
H	0.37605	1.80659	-0.00001
H	-4.91862	-0.72762	0.00020
H	-4.20536	0.65647	0.89402
H	-4.20555	0.65625	-0.89411

Compound 14 radical
Electronic energy: -514.708535208 Hartree

C	1.74631	0.05957	0.00061
C	0.55710	-0.78285	0.00036
C	-0.79657	-0.34321	0.00012
N	-1.71998	-1.35248	-0.00006
C	-3.00382	-1.02336	-0.00027
C	-3.47562	0.30034	-0.00033
C	-2.54035	1.33507	-0.00014
C	-1.18746	1.01963	0.00009
O	2.85984	-0.70783	0.00009

O	1.78747	1.28322	0.00018
C	4.10549	0.00343	-0.00039
H	0.72050	-1.85933	0.00034
H	-3.71597	-1.85263	-0.00042
H	-4.54688	0.49932	-0.00051
H	-2.86226	2.37689	-0.00017
H	-0.42244	1.79182	0.00025
H	4.88151	-0.76611	-0.00080
H	4.18785	0.63135	-0.89590
H	4.18862	0.63116	0.89519

Compound 16 imine

Electronic energy: -499.315743829 Hartree

C	-1.69763	-0.00010	0.03539
C	-0.64202	0.00377	-1.05245
C	0.77233	0.00184	-0.53694
C	1.44056	-1.20398	-0.29003
C	2.74661	-1.20817	0.20403
C	3.40428	-0.00176	0.45647
C	2.74791	1.20645	0.20936
C	1.44183	1.20583	-0.28469
O	-2.92653	0.00211	-0.50502
O	-1.49360	-0.00455	1.23215
C	-4.02531	-0.00118	0.42254
H	-0.83295	-0.87214	-1.68864
H	-0.83282	0.88428	-1.68229
H	0.93521	-2.15121	-0.48796
H	3.25331	-2.15658	0.38891
H	4.42575	-0.00315	0.83939
H	3.25563	2.15349	0.39841
H	0.93748	2.15446	-0.47839
H	-4.92752	0.00025	-0.19440
H	-3.99183	-0.89934	1.05031
H	-3.99264	0.89330	1.05560

Compound 16 enamine

Electronic energy: -499.276191343 Hartree

C	-1.74260	0.10551	0.25834
C	-0.65033	-0.68345	0.19248
C	0.75845	-0.31250	0.07166
C	1.71483	-1.35015	0.05836
C	3.07829	-1.08336	-0.04875
C	3.53185	0.23505	-0.14630
C	2.59930	1.27599	-0.13660
C	1.23327	1.01245	-0.03224
O	-2.97489	-0.42211	0.48660
O	-1.71194	1.45598	0.14186
C	-3.86222	-0.42256	-0.64886
H	-0.86752	-1.74951	0.25080
H	1.37504	-2.38487	0.13552

H	3.79089	-1.90989	-0.05542
H	4.59821	0.44852	-0.23044
H	2.93891	2.31043	-0.21401
H	0.52936	1.84127	-0.03021
H	-2.54817	1.81155	0.47424
H	-4.81311	-0.82683	-0.28956
H	-4.02004	0.59271	-1.03707
H	-3.45388	-1.06627	-1.44007

Compound 16 radical
Electronic energy: -498.674655028 Hartree

C	1.76735	0.05902	-0.00019
C	0.58101	-0.78287	-0.00023
C	-0.77140	-0.34881	-0.00011
C	-1.79377	-1.34112	-0.00015
C	-3.13485	-0.98879	-0.00001
C	-3.50633	0.36317	0.00018
C	-2.51714	1.35692	0.00025
C	-1.17121	1.01767	0.00012
O	2.88621	-0.70743	0.00040
O	1.81247	1.28360	-0.00064
C	4.12844	0.00723	0.00029
H	0.76934	-1.85636	-0.00030
H	-1.50682	-2.39373	-0.00031
H	-3.90055	-1.76534	-0.00006
H	-4.56099	0.64070	0.00030
H	-2.80698	2.40851	0.00041
H	-0.40570	1.78966	0.00017
H	4.90787	-0.75915	-0.00030
H	4.21062	0.63634	-0.89463
H	4.21120	0.63546	0.89580

Compound 18 imine
Electronic energy: -535.769558070 Hartree

C	-2.46316	1.31225	0.88891
C	-1.25554	1.09681	0.22198
C	-1.09044	-0.01118	-0.61992
C	-2.16132	-0.90043	-0.77541
C	-3.37151	-0.68759	-0.11066
C	-3.52693	0.42101	0.72430
H	-2.57404	2.18048	1.54046
H	-0.43056	1.79796	0.35783
H	-2.04758	-1.77198	-1.42308
H	-4.19369	-1.39254	-0.24340
H	-4.46981	0.58806	1.24682
C	0.20370	-0.22855	-1.38678
H	0.24034	-1.27004	-1.73732
H	0.20606	0.40311	-2.28588
C	1.44869	0.10192	-0.62874
N	1.93473	-0.66348	0.39226

C	3.23502	1.07431	0.08231
C	3.07688	-0.05158	0.85390
H	4.02337	1.81906	0.14157
H	3.65928	-0.46780	1.66808
N	2.21755	1.16363	-0.83905
C	1.37363	-1.90963	0.88518
H	0.34133	-1.75890	1.22178
H	1.38917	-2.67669	0.10088
H	1.97608	-2.25592	1.72925

Compound 18 enamine

Electronic energy: -535.741142546 Hartree

C	-3.02487	-1.31152	0.24338
C	-1.66948	-0.97858	0.26425
C	-1.22327	0.34890	0.02687
C	-2.24343	1.31365	-0.20169
C	-3.59150	0.97690	-0.21854
C	-4.00366	-0.34626	-0.00499
H	-3.31798	-2.34567	0.43661
H	-0.96405	-1.76578	0.52834
H	-1.95048	2.35180	-0.37408
H	-4.33439	1.75586	-0.40357
H	-5.06085	-0.61306	-0.02278
C	0.15116	0.78461	0.02926
H	0.31470	1.86081	0.01383
C	1.30231	0.01291	-0.00747
N	2.59065	0.50448	0.06346
C	2.80649	-1.68429	-0.21188
C	3.50488	-0.53377	-0.07403
H	3.14127	-2.70681	-0.32713
H	4.57274	-0.35850	-0.04246
N	1.45769	-1.34954	-0.14228
C	2.91479	1.90260	0.21508
H	2.57505	2.48298	-0.65510
H	2.44073	2.31063	1.11865
H	3.99978	2.00400	0.30609
H	0.70458	-1.97168	-0.38809

Compound 18 radical

Electronic energy: -535.137180294 Hartree

C	-2.99439	-1.34132	-0.00003
C	-1.63943	-1.02610	-0.00003
C	-1.21073	0.32755	0.00004
C	-2.21394	1.33342	0.00003
C	-3.56468	1.00817	-0.00000
C	-3.96741	-0.33373	-0.00003
H	-3.30053	-2.38894	-0.00006
H	-0.88863	-1.81348	-0.00005
H	-1.91064	2.38212	0.00006
H	-4.31231	1.80291	0.00000

H	-5.02740	-0.59067	-0.00004
C	0.16276	0.73844	0.00007
H	0.33302	1.81487	0.00012
C	1.32345	-0.06896	0.00003
N	2.59644	0.48377	0.00004
C	2.73495	-1.70802	0.00000
C	3.48941	-0.54605	0.00000
H	3.10219	-2.73056	0.00000
H	4.56052	-0.37915	-0.00003
N	1.41000	-1.41860	0.00002
C	2.93239	1.89588	-0.00008
H	2.53158	2.38787	-0.89492
H	2.53186	2.38798	0.89482
H	4.02113	1.99507	-0.00026

Compound 20 imine

Electronic energy: -304.782976532 Hartree

C	-1.20679	0.97212	-0.00000
C	-1.85596	-0.23817	0.00000
N	-0.94169	-1.26754	0.00000
C	0.25236	-0.68748	-0.00000
N	0.13724	0.67289	-0.00001
C	1.56319	-1.39357	0.00000
C	1.22961	1.62942	0.00001
H	-1.56552	1.99530	-0.00001
H	-2.92666	-0.42134	0.00001
H	2.16239	-1.13976	-0.88679
H	2.16233	-1.13985	0.88685
H	1.38469	-2.47336	-0.00006
H	0.81256	2.64018	-0.00015
H	1.85334	1.50386	0.89374
H	1.85353	1.50366	-0.89357

Compound 20 enamine

Electronic energy: -304.740653221 Hartree

C	-0.81855	-1.27731	-0.00013
C	-1.81478	-0.36219	0.00015
N	-1.21377	0.89360	0.00018
C	0.16526	0.77900	-0.00012
N	0.39374	-0.58664	-0.00047
C	1.10118	1.78064	-0.00011
C	1.71137	-1.16632	0.00029
H	-0.85772	-2.35950	-0.00034
H	-2.88974	-0.48888	0.00031
H	-1.70098	1.77476	-0.00007
H	0.78678	2.82114	0.00045
H	2.16189	1.54873	-0.00017
H	1.62193	-2.25665	-0.00033
H	2.27628	-0.85527	-0.89169
H	2.27496	-0.85608	0.89342

Compound 20 radical

Electronic energy: -304.133444867 Hartree

C	-0.80558	-1.24844	-0.00009
C	-1.80672	-0.28466	-0.00002
N	-1.27747	0.95956	-0.00002
C	0.07396	0.78856	-0.00005
N	0.37484	-0.56661	-0.00010
C	1.02338	1.80657	0.00000
C	1.70410	-1.14504	0.00019
H	-0.84698	-2.33210	-0.00016
H	-2.87998	-0.45642	-0.00001
H	0.68923	2.84052	0.00004
H	2.08964	1.59698	0.00002
H	1.61446	-2.23472	-0.00050
H	2.25896	-0.83289	-0.89363
H	2.25818	-0.83392	0.89487

Compound 22 imine

Electronic energy: -669.927113826 Hartree

C	4.01563	-1.35954	-0.37709
C	4.36210	-0.05435	-0.77347
C	3.48044	1.01687	-0.62011
C	2.23308	0.74043	-0.05436
C	1.91429	-0.56734	0.33066
C	2.77079	-1.64891	0.18998
N	1.14358	1.56478	0.23432
C	0.26053	0.76857	0.75382
O	0.64616	-0.54366	0.85001
C	-1.10276	1.10783	1.24806
C	-2.20958	0.45279	0.44167
C	-3.07566	-0.47205	1.03434
C	-4.10068	-1.06529	0.29201
C	-4.26915	-0.73911	-1.05465
C	-3.40767	0.18430	-1.65447
C	-2.38605	0.77585	-0.91082
H	4.73543	-2.16687	-0.51506
H	5.34539	0.12136	-1.21106
H	3.75167	2.02651	-0.92734
H	2.49562	-2.65584	0.49972
H	-1.18092	0.80099	2.30012
H	-1.19125	2.20049	1.21117
H	-2.95060	-0.73166	2.08704
H	-4.76873	-1.78362	0.76939
H	-5.06864	-1.20076	-1.63562
H	-3.53428	0.44700	-2.70574
H	-1.71985	1.49800	-1.38638

Compound 22 enamine

Electronic energy: -669.909229578 Hartree

C	-1.55359	4.14727	0.00000
C	-2.88423	3.71834	0.00000
C	-3.22184	2.35557	0.00000
C	-2.16879	1.44762	0.00000
C	-0.84325	1.89114	0.00000
C	-0.49183	3.22485	0.00000
N	-2.09884	0.06727	0.00000
C	-0.77738	-0.33751	0.00000
O	0.00000	0.79700	0.00000
C	-0.30051	-1.60930	0.00000
C	1.07672	-2.07391	0.00000
C	1.30885	-3.46941	0.00000
C	2.59747	-3.99706	0.00000
C	3.71022	-3.14963	0.00000
C	3.50258	-1.76720	0.00000
C	2.21411	-1.23285	0.00000
H	-1.33010	5.21403	0.00000
H	-3.68429	4.45921	0.00000
H	-4.26001	2.02728	0.00000
H	0.54920	3.54358	0.00000
H	-2.87948	-0.57117	0.00000
H	-1.07296	-2.37852	0.00000
H	0.45282	-4.14722	0.00000
H	2.73446	-5.08004	0.00000
H	4.72088	-3.55960	0.00000
H	4.35863	-1.08967	0.00000
H	2.09142	-0.15236	0.00000

Compound 22 radical
Electronic energy: -669.293439398 Hartree

C	-1.63943	4.10305	0.00000
C	-2.96605	3.62617	0.00000
C	-3.25437	2.26362	0.00000
C	-2.16686	1.37669	0.00000
C	-0.85385	1.88289	0.00000
C	-0.54382	3.23329	0.00000
N	-2.11700	0.00192	0.00000
C	-0.82162	-0.29357	0.00000
O	0.00000	0.81846	0.00000
C	-0.29382	-1.59689	0.00000
C	1.06587	-2.04557	0.00000
C	1.29458	-3.44769	0.00000
C	2.58138	-3.96898	0.00000
C	3.68751	-3.11011	0.00000
C	3.48522	-1.72427	0.00000
C	2.20096	-1.19235	0.00000
H	-1.46134	5.17869	0.00000
H	-3.78462	4.34653	0.00000
H	-4.28020	1.89661	0.00000
H	0.48263	3.59614	0.00000

H	-1.06849	-2.36316	0.00000
H	0.43679	-4.12184	0.00000
H	2.72795	-5.04974	0.00000
H	4.69914	-3.51719	0.00000
H	4.34358	-1.05122	0.00000
H	2.06931	-0.11368	0.00000

Compound 24 imine

Electronic energy: -992.891293722 Hartree

C	-4.36704	-1.09455	0.44243
C	-4.59669	0.28922	0.54730
C	-3.57272	1.20372	0.32426
C	-2.29734	0.72528	-0.01127
C	-2.08135	-0.66900	-0.11389
C	-3.10780	-1.59059	0.11220
N	-1.18524	1.51472	-0.25837
C	-0.14657	0.79428	-0.54601
S	-0.40958	-0.95813	-0.53308
C	1.19464	1.35980	-0.91798
C	2.36932	0.62731	-0.30725
C	3.17582	-0.21134	-1.08537
C	4.25391	-0.89449	-0.51516
C	4.53888	-0.74323	0.84270
C	3.74092	0.09524	1.62729
C	2.66455	0.77398	1.05585
H	-5.18580	-1.79209	0.62206
H	-5.59281	0.64884	0.80728
H	-3.74432	2.27722	0.40389
H	-2.93247	-2.66288	0.03228
H	1.28039	1.34340	-2.01483
H	1.18745	2.41367	-0.61039
H	2.96234	-0.33016	-2.14919
H	4.87388	-1.54285	-1.13598
H	5.38157	-1.27292	1.28904
H	3.96074	0.22293	2.68823
H	2.04788	1.42820	1.67492

Compound 24 enamine

Electronic energy: -992.876017420 Hartree

C	4.67211	-0.90078	-0.00013
C	4.82091	0.49061	-0.00009
C	3.71079	1.33950	-0.00001
C	2.43660	0.76561	0.00003
C	2.29161	-0.63168	0.00001
C	3.39569	-1.47622	-0.00006
N	1.22337	1.41876	0.00016
C	0.08137	0.63494	0.00006
S	0.57843	-1.07434	0.00015
C	-1.18253	1.14477	0.00000
C	-2.47173	0.47610	-0.00007

C	-2.66831	-0.92363	-0.00005
C	-3.95075	-1.47375	-0.00004
C	-5.08294	-0.65572	-0.00004
C	-4.91108	0.73300	-0.00003
C	-3.63379	1.28471	-0.00004
H	5.55194	-1.54410	-0.00020
H	5.82064	0.92613	-0.00013
H	3.83329	2.42240	0.00006
H	3.27131	-2.55876	-0.00007
H	1.15212	2.42711	-0.00025
H	-1.22750	2.23666	-0.00006
H	-1.82106	-1.60725	0.00000
H	-4.06353	-2.55922	-0.00002
H	-6.08243	-1.09194	-0.00003
H	-5.78120	1.39191	-0.00001
H	-3.51971	2.37051	-0.00004

Compound 24 radical
Electronic energy: -992.256930407 Hartree

C	-4.67092	-0.82576	0.00004
C	-4.78375	0.57883	0.00001
C	-3.65599	1.38734	-0.00001
C	-2.38155	0.78402	-0.00000
C	-2.28887	-0.63387	0.00003
C	-3.42323	-1.44740	0.00005
N	-1.19407	1.45781	-0.00002
C	-0.15245	0.62927	-0.00001
S	-0.60758	-1.09791	0.00003
C	1.16694	1.13105	-0.00003
C	2.44256	0.47825	-0.00003
C	2.64939	-0.92513	-0.00001
C	3.93410	-1.45656	-0.00000
C	5.05483	-0.61762	-0.00002
C	4.87585	0.77111	-0.00004
C	3.59576	1.30847	-0.00004
H	-5.57298	-1.43830	0.00006
H	-5.77415	1.03502	0.00001
H	-3.73610	2.47431	-0.00003
H	-3.34025	-2.53366	0.00007
H	1.19051	2.22244	-0.00005
H	1.80685	-1.61342	0.00001
H	4.06587	-2.53906	0.00001
H	6.05872	-1.04326	-0.00002
H	5.74158	1.43437	-0.00005
H	3.46381	2.39145	-0.00006

Compound 28 imine
Electronic energy: -633.015763971 Hartree

C	-2.18581	-0.10854	0.60431
C	-2.48257	-1.27331	-0.11798

C	-3.49928	-1.24527	-1.06750
C	-4.19166	-0.05083	-1.27370
C	-3.82910	1.05612	-0.50963
H	-1.91992	-2.18859	0.06823
H	-3.75034	-2.14103	-1.63732
H	-4.99632	0.02267	-2.00497
H	-4.35102	2.00769	-0.63945
N	-2.85445	1.03910	0.41086
C	-1.07072	-0.09462	1.62794
H	-1.10382	-1.02354	2.21313
H	-1.25458	0.74225	2.31477
C	0.29961	0.04396	0.99467
C	1.19867	-1.02353	0.96383
C	0.70254	1.25523	0.40752
C	2.46143	-0.90993	0.36904
H	0.91900	-1.97650	1.41703
C	1.95130	1.38937	-0.18708
H	0.02484	2.11036	0.41680
C	2.84130	0.30391	-0.21224
H	3.12950	-1.76868	0.37180
H	2.25784	2.33410	-0.63722
O	4.04201	0.53031	-0.81627
C	4.97293	-0.54449	-0.86192
H	4.56627	-1.39979	-1.42188
H	5.85417	-0.15594	-1.38080
H	5.25763	-0.86771	0.15044

Compound 28 enamine

Electronic energy: -632.991213389 Hartree

C	2.18992	0.50012	-0.07879
C	3.53997	-1.49432	0.34253
C	4.71470	-0.81603	0.23466
C	4.64693	0.59552	-0.02387
H	3.39117	2.28961	-0.36023
H	1.50232	-1.40591	0.35298
H	3.48209	-2.56094	0.55175
H	5.66307	-1.33552	0.34724
H	5.56870	1.17365	-0.10304
C	0.97337	1.12918	-0.21545
H	1.03770	2.21401	-0.31043
C	-0.37559	0.58300	-0.17106
C	-0.73039	-0.75225	-0.48446
C	-1.44652	1.44061	0.16296
C	-2.04744	-1.19756	-0.42802
H	0.01684	-1.45921	-0.84625
C	-2.77171	1.01017	0.21843
H	-1.23045	2.48591	0.39238
C	-3.08209	-0.32602	-0.06636
H	-2.29049	-2.22983	-0.68243
H	-3.54821	1.72418	0.48669

O	-4.34000	-0.85849	-0.03184
C	-5.41103	0.00597	0.32326
H	-6.31558	-0.60906	0.29676
H	-5.27607	0.41325	1.33666
H	-5.51159	0.83363	-0.39513
C	3.44379	1.21824	-0.16804
N	2.33815	-0.86365	0.18757

Compound 28 radical
Electronic energy: -632.991213389 Hartree

C	2.18992	0.50012	-0.07879
C	3.53997	-1.49432	0.34253
C	4.71470	-0.81603	0.23466
C	4.64693	0.59552	-0.02387
H	3.39117	2.28961	-0.36023
H	1.50232	-1.40591	0.35298
H	3.48209	-2.56094	0.55175
H	5.66307	-1.33552	0.34724
H	5.56870	1.17365	-0.10304
C	0.97337	1.12918	-0.21545
H	1.03770	2.21401	-0.31043
C	-0.37559	0.58300	-0.17106
C	-0.73039	-0.75225	-0.48446
C	-1.44652	1.44061	0.16296
C	-2.04744	-1.19756	-0.42802
H	0.01684	-1.45921	-0.84625
C	-2.77171	1.01017	0.21843
H	-1.23045	2.48591	0.39238
C	-3.08209	-0.32602	-0.06636
H	-2.29049	-2.22983	-0.68243
H	-3.54821	1.72418	0.48669
O	-4.34000	-0.85849	-0.03184
C	-5.41103	0.00597	0.32326
H	-6.31558	-0.60906	0.29676
H	-5.27607	0.41325	1.33666
H	-5.51159	0.83363	-0.39513
C	3.44379	1.21824	-0.16804
N	2.33815	-0.86365	0.18757

Compound 29 imine
Electronic energy: -557.826847988 Hartree

C	1.76721	0.24438	0.58510
C	2.18557	1.17635	-0.37522
C	3.24121	0.85346	-1.22270
C	3.85028	-0.39522	-1.08962
C	3.36942	-1.25649	-0.10564
H	1.68692	2.14300	-0.45120
H	3.58602	1.56510	-1.97420
H	4.68108	-0.69518	-1.72791
H	3.82429	-2.24073	0.03253

N	2.35600	-0.95451	0.71802
C	0.60579	0.54905	1.50713
H	0.65823	1.59975	1.82238
H	0.71134	-0.07830	2.40200
C	-0.73761	0.29256	0.85214
C	-1.56173	1.34779	0.44955
C	-1.18656	-1.01697	0.62259
C	-2.79367	1.10512	-0.16676
H	-1.24297	2.37780	0.62130
C	-2.41445	-1.25667	0.00905
H	-0.56561	-1.86019	0.92973
C	-3.24301	-0.19902	-0.39964
H	-3.41630	1.95013	-0.46758
H	-2.73911	-2.28685	-0.15313
C	-4.56921	-0.46960	-1.05947
H	-5.23017	-1.04942	-0.39868
H	-5.08352	0.46385	-1.31924
H	-4.44151	-1.05578	-1.98121

Compound 29 enamine

Electronic energy: -557.803350146 Hartree

C	1.76458	0.49068	-0.07857
C	3.10701	-1.51802	0.28436
C	4.28439	-0.84435	0.17194
C	4.22159	0.57064	-0.06020
H	2.97064	2.27828	-0.34568
H	1.07020	-1.41681	0.32314
H	3.04517	-2.58787	0.47463
H	5.23109	-1.37129	0.26136
H	5.14554	1.14462	-0.14343
C	0.54871	1.13171	-0.18051
H	0.62171	2.21766	-0.25319
C	-0.80217	0.60010	-0.12195
C	-1.17936	-0.73129	-0.41270
C	-1.86018	1.48253	0.21392
C	-2.51039	-1.15006	-0.33287
H	-0.44971	-1.45477	-0.77800
C	-3.18094	1.05905	0.28736
H	-1.62369	2.52735	0.42602
C	-3.54039	-0.27519	0.02685
H	-2.74935	-2.18824	-0.57401
H	-3.95568	1.78125	0.55591
C	-4.97243	-0.72863	0.12447
H	-5.35353	-0.63172	1.15247
H	-5.62930	-0.12429	-0.51858
H	-5.07894	-1.77898	-0.17470
N	1.90785	-0.87690	0.15815
C	3.01974	1.20334	-0.17427

Compound 29 radical

Electronic energy: -557.803350146 Hartree

C	1.76458	0.49068	-0.07857
C	3.10701	-1.51802	0.28436
C	4.28439	-0.84435	0.17194
C	4.22159	0.57064	-0.06020
H	2.97064	2.27828	-0.34568
H	1.07020	-1.41681	0.32314
H	3.04517	-2.58787	0.47463
H	5.23109	-1.37129	0.26136
H	5.14554	1.14462	-0.14343
C	0.54871	1.13171	-0.18051
H	0.62171	2.21766	-0.25319
C	-0.80217	0.60010	-0.12195
C	-1.17936	-0.73129	-0.41270
C	-1.86018	1.48253	0.21392
C	-2.51039	-1.15006	-0.33287
H	-0.44971	-1.45477	-0.77800
C	-3.18094	1.05905	0.28736
H	-1.62369	2.52735	0.42602
C	-3.54039	-0.27519	0.02685
H	-2.74935	-2.18824	-0.57401
H	-3.95568	1.78125	0.55591
C	-4.97243	-0.72863	0.12447
H	-5.35353	-0.63172	1.15247
H	-5.62930	-0.12429	-0.51858
H	-5.07894	-1.77898	-0.17470
N	1.90785	-0.87690	0.15815
C	3.01974	1.20334	-0.17427

Compound 30 imine

Electronic energy: -955.984395777 Hartree

C	-1.52120	1.14817	-1.19245
H	-1.65314	0.89820	-2.25324
H	-1.70387	2.22578	-1.07834
C	-0.10379	0.83720	-0.75757
C	0.67831	1.78614	-0.08594
C	0.46627	-0.41604	-1.01203
C	1.97988	1.49667	0.31946
H	0.26692	2.77520	0.12372
C	1.76893	-0.72134	-0.61359
H	-0.11468	-1.17675	-1.53641
C	2.53911	0.23640	0.05954
H	2.56544	2.25717	0.83859
H	2.17141	-1.70755	-0.83679
C	-2.56283	0.38016	-0.40596
C	-2.81341	0.68486	0.93994
N	-3.21948	-0.59723	-1.04935
C	-3.77216	-0.04426	1.63564
H	-2.26043	1.48689	1.42979
C	-4.13926	-1.29628	-0.36800

C	-4.45418	-1.06370	0.96846
H	-3.98679	0.17870	2.68171
H	-4.65311	-2.08416	-0.92458
H	-5.21407	-1.66336	1.46905
S	4.20781	-0.03322	0.61445
C	4.56593	-1.73701	0.10467
H	4.51756	-1.84595	-0.98538
H	5.59300	-1.92763	0.43685
H	3.89353	-2.45023	0.59625

Compound 30 enamine

Electronic energy: -955.960430926 Hartree

C	-2.58001	0.47575	0.07908
C	-3.88642	-1.56870	-0.18220
C	-5.07247	-0.92330	-0.00661
C	-5.03315	0.49336	0.20408
H	-1.86142	-1.41551	-0.34555
H	-3.80680	-2.63864	-0.36397
H	-6.00860	-1.47534	-0.03312
H	-5.96496	1.04487	0.33592
C	-1.37590	1.15371	0.10567
H	-1.47971	2.23777	0.16345
C	-0.01791	0.66806	-0.00688
C	0.41127	-0.65923	0.24585
C	0.99760	1.59438	-0.36453
C	1.74830	-1.03446	0.11724
H	-0.27958	-1.41402	0.62129
C	2.32703	1.22039	-0.50009
H	0.71713	2.63229	-0.55364
C	2.72369	-0.10662	-0.26419
H	2.03916	-2.06352	0.33177
H	3.06981	1.96256	-0.79641
C	-3.84253	1.15831	0.23937
H	-3.81123	2.23596	0.39585
N	-2.70063	-0.89385	-0.13627
S	4.42703	-0.61051	-0.47139
C	5.17332	-0.02599	1.09271
H	6.23726	-0.29025	1.04718
H	5.07444	1.06170	1.18582
H	4.70944	-0.52671	1.95048

Compound 30 radical

Electronic energy: -955.960430926 Hartree

C	-2.58001	0.47575	0.07908
C	-3.88642	-1.56870	-0.18220
C	-5.07247	-0.92330	-0.00661
C	-5.03315	0.49336	0.20408
H	-1.86142	-1.41551	-0.34555
H	-3.80680	-2.63864	-0.36397
H	-6.00860	-1.47534	-0.03312

H	-5.96496	1.04487	0.33592
C	-1.37590	1.15371	0.10567
H	-1.47971	2.23777	0.16345
C	-0.01791	0.66806	-0.00688
C	0.41127	-0.65923	0.24585
C	0.99760	1.59438	-0.36453
C	1.74830	-1.03446	0.11724
H	-0.27958	-1.41402	0.62129
C	2.32703	1.22039	-0.50009
H	0.71713	2.63229	-0.55364
C	2.72369	-0.10662	-0.26419
H	2.03916	-2.06352	0.33177
H	3.06981	1.96256	-0.79641
C	-3.84253	1.15831	0.23937
H	-3.81123	2.23596	0.39585
N	-2.70063	-0.89385	-0.13627
S	4.42703	-0.61051	-0.47139
C	5.17332	-0.02599	1.09271
H	6.23726	-0.29025	1.04718
H	5.07444	1.06170	1.18582
H	4.70944	-0.52671	1.95048

Compound 31 imine
Electronic energy: -978.088381203 Hartree

C	2.16549	0.26734	-0.53430
C	2.99563	-0.83095	-0.78658
C	3.99406	-1.15454	0.13125
C	4.13406	-0.36846	1.27317
C	3.26350	0.70996	1.43777
H	2.86063	-1.42167	-1.69277
H	4.65261	-2.00614	-0.04500
H	4.89918	-0.57832	2.02020
H	3.34684	1.35236	2.31826
N	2.30180	1.02786	0.56450
C	1.05928	0.65409	-1.49369
H	1.18728	0.09749	-2.43174
H	1.15754	1.72295	-1.72589
C	-0.32122	0.38985	-0.93023
C	-0.79477	-0.92254	-0.79351
C	-1.15274	1.44054	-0.52733
C	-2.05932	-1.18662	-0.26858
H	-0.16889	-1.76097	-1.10309
C	-2.42303	1.20026	0.00219
H	-0.80958	2.47106	-0.62750
C	-2.85993	-0.11547	0.12569
H	-2.41911	-2.20985	-0.16973
H	-3.06220	2.02614	0.31135
Cl	-4.45664	-0.43464	0.78701

Compound 31 enamine

Electronic energy: -978.065786390 Hartree

C	-2.17425	0.47910	0.06718
C	-3.45728	-1.56895	-0.27669
C	-4.65343	-0.92881	-0.16347
C	-4.63099	0.48737	0.05662
H	-3.42864	2.23314	0.32489
H	-1.42721	-1.40832	-0.33016
H	-3.36387	-2.63755	-0.45985
H	-5.58463	-1.48386	-0.24454
H	-5.57076	1.03492	0.13955
C	-0.97678	1.16027	0.16348
H	-1.08509	2.24379	0.22138
C	0.38733	0.67505	0.12020
C	0.80176	-0.64839	0.41122
C	1.41845	1.59636	-0.19717
C	2.14069	-1.03496	0.34746
H	0.09273	-1.39629	0.76528
C	2.75617	1.22708	-0.26101
H	1.15190	2.63362	-0.40658
C	3.10979	-0.09815	0.00119
H	2.42549	-2.05959	0.58402
H	3.51998	1.96125	-0.51573
Cl	4.79815	-0.58236	-0.08998
C	-3.44704	1.15623	0.16119
N	-2.27784	-0.89060	-0.16140

Compound 31 radical

Electronic energy: -978.065786390 Hartree

C	-2.17425	0.47910	0.06718
C	-3.45728	-1.56895	-0.27669
C	-4.65343	-0.92881	-0.16347
C	-4.63099	0.48737	0.05662
H	-3.42864	2.23314	0.32489
H	-1.42721	-1.40832	-0.33016
H	-3.36387	-2.63755	-0.45985
H	-5.58463	-1.48386	-0.24454
H	-5.57076	1.03492	0.13955
C	-0.97678	1.16027	0.16348
H	-1.08509	2.24379	0.22138
C	0.38733	0.67505	0.12020
C	0.80176	-0.64839	0.41122
C	1.41845	1.59636	-0.19717
C	2.14069	-1.03496	0.34746
H	0.09273	-1.39629	0.76528
C	2.75617	1.22708	-0.26101
H	1.15190	2.63362	-0.40658
C	3.10979	-0.09815	0.00119
H	2.42549	-2.05959	0.58402
H	3.51998	1.96125	-0.51573
Cl	4.79815	-0.58236	-0.08998

C	-3.44704	1.15623	0.16119
N	-2.27784	-0.89060	-0.16140

Compound 32 imine

Electronic energy: -746.346318235 Hartree

C	2.90334	-0.34035	-0.44200
C	3.23447	-1.10781	0.68268
C	4.16634	-0.61383	1.59126
C	4.74048	0.63481	1.35126
C	4.35204	1.32560	0.20487
H	2.76773	-2.08078	0.83826
H	4.44257	-1.19503	2.47206
H	5.47577	1.06393	2.03152
H	4.78383	2.30410	-0.01996
N	3.45795	0.85881	-0.67739
C	1.87610	-0.83137	-1.44056
H	1.97395	-1.91755	-1.56307
H	2.09030	-0.35921	-2.40846
C	0.45932	-0.50200	-1.01749
C	-0.43061	-1.51274	-0.63200
C	0.01212	0.82859	-0.99765
C	-1.73169	-1.21040	-0.23326
H	-0.10331	-2.55330	-0.64611
C	-1.28421	1.13748	-0.60125
H	0.69041	1.62841	-1.29599
C	-2.16906	0.12038	-0.21379
H	-2.40896	-2.01017	0.06084
H	-1.62718	2.17139	-0.59010
C	-3.54478	0.49861	0.20153
O	-3.96191	1.64408	0.23387
O	-4.29429	-0.56165	0.54321
C	-5.63879	-0.27245	0.95710
H	-6.08500	-1.24122	1.19539
H	-5.63494	0.37608	1.84112
H	-6.19162	0.21502	0.14533

Compound 32 enamine

Electronic energy: -746.327343444 Hartree

C	2.93180	0.40978	-0.05778
C	4.05073	-1.72728	0.28328
C	5.29260	-1.18237	0.14894
C	5.37826	0.22613	-0.07934
H	4.31043	2.05965	-0.34483
H	2.04266	-1.40588	0.35928
H	3.87565	-2.78377	0.47513
H	6.17837	-1.80829	0.22161
H	6.35644	0.69797	-0.17942
C	1.78636	1.18830	-0.14656
H	1.98896	2.25798	-0.20737
C	0.39486	0.83056	-0.10965

C	-0.14118	-0.47075	-0.31620
C	-0.55649	1.86742	0.11258
C	-1.50659	-0.71721	-0.26054
H	0.49833	-1.30649	-0.59708
C	-1.91495	1.62074	0.16954
H	-0.19310	2.88686	0.25233
C	-2.42110	0.31747	-0.00407
H	-1.87128	-1.72835	-0.43495
H	-2.61222	2.43782	0.35396
C	-3.87488	0.09822	0.07089
O	-4.70342	0.97758	0.26458
O	-4.22230	-1.19517	-0.09724
C	-5.62689	-1.47727	-0.04138
H	-6.16019	-0.93735	-0.83327
H	-5.71478	-2.55656	-0.19202
H	-6.03843	-1.19376	0.93491
C	4.24697	0.98604	-0.17266
N	2.92972	-0.95684	0.18153

Compound 32 radical

Electronic energy: -746.327343444 Hartree

C	2.93180	0.40978	-0.05778
C	4.05073	-1.72728	0.28328
C	5.29260	-1.18237	0.14894
C	5.37826	0.22613	-0.07934
H	4.31043	2.05965	-0.34483
H	2.04266	-1.40588	0.35928
H	3.87565	-2.78377	0.47513
H	6.17837	-1.80829	0.22161
H	6.35644	0.69797	-0.17942
C	1.78636	1.18830	-0.14656
H	1.98896	2.25798	-0.20737
C	0.39486	0.83056	-0.10965
C	-0.14118	-0.47075	-0.31620
C	-0.55649	1.86742	0.11258
C	-1.50659	-0.71721	-0.26054
H	0.49833	-1.30649	-0.59708
C	-1.91495	1.62074	0.16954
H	-0.19310	2.88686	0.25233
C	-2.42110	0.31747	-0.00407
H	-1.87128	-1.72835	-0.43495
H	-2.61222	2.43782	0.35396
C	-3.87488	0.09822	0.07089
O	-4.70342	0.97758	0.26458
O	-4.22230	-1.19517	-0.09724
C	-5.62689	-1.47727	-0.04138
H	-6.16019	-0.93735	-0.83327
H	-5.71478	-2.55656	-0.19202
H	-6.03843	-1.19376	0.93491
C	4.24697	0.98604	-0.17266

N	2.92972	-0.95684	0.18153

Compound 33 imine

Electronic energy: -610.738747358 Hartree

C	-2.02322	0.41880	-0.43102
C	-2.49523	0.97842	0.76322
C	-3.51649	0.33444	1.45680
C	-4.03410	-0.85282	0.93880
C	-3.50142	-1.33478	-0.25571
H	-2.06789	1.90867	1.13831
H	-3.90430	0.75381	2.38607
H	-4.83491	-1.39296	1.44323
H	-3.88393	-2.25972	-0.69451
N	-2.52194	-0.72145	-0.93343
C	-0.90208	1.07752	-1.20676
H	-0.98422	2.16792	-1.11805
H	-1.02039	0.81382	-2.26635
C	0.46856	0.64153	-0.73278
C	1.35503	1.56121	-0.15767
C	0.87629	-0.69503	-0.86693
C	2.61858	1.16803	0.27537
H	1.05626	2.60420	-0.04856
C	2.13328	-1.10627	-0.44063
H	0.19685	-1.42230	-1.31113
C	3.01187	-0.17147	0.13425
H	3.29991	1.89286	0.71908
H	2.44129	-2.14522	-0.55176
C	4.30935	-0.58635	0.57450
N	5.36462	-0.92434	0.93273

Compound 33 enamine

Electronic energy: -610.720788673 Hartree

C	-2.02706	0.45885	0.04143
C	-3.34702	-1.56852	-0.23264
C	-4.53006	-0.90231	-0.11344
C	-4.47886	0.51310	0.07179
H	-3.23729	2.24219	0.27956
H	-1.31895	-1.44526	-0.32261
H	-3.27479	-2.64213	-0.39291
H	-5.47217	-1.44201	-0.16622
H	-5.40640	1.08004	0.15886
C	-0.81142	1.12911	0.10503
H	-0.91368	2.21426	0.12914
C	0.53855	0.64449	0.08463
C	0.95275	-0.69631	0.32471
C	1.58126	1.58633	-0.15681
C	2.28704	-1.07014	0.28014
H	0.24091	-1.46457	0.62192
C	2.91245	1.22343	-0.20495
H	1.31319	2.63098	-0.32031

C	3.28874	-0.12219	0.00136
H	2.56339	-2.10549	0.47829
H	3.67618	1.97478	-0.40501
C	4.65706	-0.51003	-0.05379
N	5.77986	-0.82678	-0.09973
N	-2.15690	-0.90677	-0.15556
C	-3.27862	1.16287	0.14027

Compound 33 radical

Electronic energy: -610.720788673 Hartree

C	-2.02706	0.45885	0.04143
C	-3.34702	-1.56852	-0.23264
C	-4.53006	-0.90231	-0.11344
C	-4.47886	0.51310	0.07179
H	-3.23729	2.24219	0.27956
H	-1.31895	-1.44526	-0.32261
H	-3.27479	-2.64213	-0.39291
H	-5.47217	-1.44201	-0.16622
H	-5.40640	1.08004	0.15886
C	-0.81142	1.12911	0.10503
H	-0.91368	2.21426	0.12914
C	0.53855	0.64449	0.08463
C	0.95275	-0.69631	0.32471
C	1.58126	1.58633	-0.15681
C	2.28704	-1.07014	0.28014
H	0.24091	-1.46457	0.62192
C	2.91245	1.22343	-0.20495
H	1.31319	2.63098	-0.32031
C	3.28874	-0.12219	0.00136
H	2.56339	-2.10549	0.47829
H	3.67618	1.97478	-0.40501
C	4.65706	-0.51003	-0.05379
N	5.77986	-0.82678	-0.09973
N	-2.15690	-0.90677	-0.15556
C	-3.27862	1.16287	0.14027

Compound 34a imine

Electronic energy: -597.137314400 Hartree

C	1.46157	-0.65058	-0.61972
C	2.16816	-1.66904	0.02972
C	3.26823	-1.32586	0.81285
C	3.62757	0.01539	0.92017
C	2.87349	0.97564	0.23345
H	1.86115	-2.70887	-0.08045
H	3.84051	-2.09589	1.33248
H	4.48331	0.31829	1.52338
N	1.81356	0.63943	-0.51866
C	0.24646	-0.95674	-1.47060
H	0.21913	-2.03523	-1.67834
H	0.35562	-0.43598	-2.43146

C	-1.05183	-0.53015	-0.81601
C	-1.55662	-1.22460	0.29388
C	-1.78146	0.56365	-1.29003
C	-2.74599	-0.83414	0.90587
H	-1.01212	-2.08628	0.68535
C	-2.97493	0.95538	-0.67442
H	-1.41652	1.12061	-2.15496
C	-3.47935	0.26644	0.43363
H	-3.11524	-1.39662	1.76623
H	-3.52308	1.81338	-1.06893
C	3.21531	2.43772	0.30062
H	2.36540	3.01135	0.69616
H	4.08759	2.61770	0.93915
H	3.42999	2.82686	-0.70465
C	-4.76426	0.68020	1.10104
H	-5.50368	-0.13369	1.07797
H	-4.59843	0.93405	2.15826
H	-5.20664	1.55444	0.60769

Compound 34a enamine

Electronic energy: -597.114587984 Hartree

C	1.46765	0.88203	-0.07957
C	2.64927	1.71015	-0.13909
C	3.90015	1.17159	-0.08334
C	4.08732	-0.24295	0.04668
C	2.98357	-1.04424	0.12821
H	0.94431	-1.09353	0.20281
H	2.50572	2.78581	-0.23408
H	4.77316	1.82390	-0.13651
H	5.08158	-0.68140	0.08409
N	1.73494	-0.47950	0.06093
C	0.19572	1.41345	-0.12477
H	0.16753	2.50389	-0.12236
C	-1.09965	0.75731	-0.09410
C	-1.35666	-0.58290	-0.46594
C	-2.23175	1.51612	0.29899
C	-2.64310	-1.12616	-0.40830
H	-0.56763	-1.21280	-0.87782
C	-3.50746	0.96923	0.35024
H	-2.09021	2.56348	0.57400
C	-3.74570	-0.37398	0.00881
H	-2.78812	-2.16508	-0.71283
H	-4.34265	1.59932	0.66560
C	3.02454	-2.53078	0.28320
H	2.50359	-2.84358	1.20032
H	2.52872	-3.02685	-0.56404
H	4.06052	-2.87917	0.33510
C	-5.12869	-0.96349	0.08479
H	-5.84824	-0.37702	-0.50548
H	-5.14294	-1.99444	-0.29069

H	-5.50121	-0.97835	1.12049

Compound 34a radical

Electronic energy: -597.114587984 Hartree

C	1.46765	0.88203	-0.07957
C	2.64927	1.71015	-0.13909
C	3.90015	1.17159	-0.08334
C	4.08732	-0.24295	0.04668
C	2.98357	-1.04424	0.12821
H	0.94431	-1.09353	0.20281
H	2.50572	2.78581	-0.23408
H	4.77316	1.82390	-0.13651
H	5.08158	-0.68140	0.08409
N	1.73494	-0.47950	0.06093
C	0.19572	1.41345	-0.12477
H	0.16753	2.50389	-0.12236
C	-1.09965	0.75731	-0.09410
C	-1.35666	-0.58290	-0.46594
C	-2.23175	1.51612	0.29899
C	-2.64310	-1.12616	-0.40830
H	-0.56763	-1.21280	-0.87782
C	-3.50746	0.96923	0.35024
H	-2.09021	2.56348	0.57400
C	-3.74570	-0.37398	0.00881
H	-2.78812	-2.16508	-0.71283
H	-4.34265	1.59932	0.66560
C	3.02454	-2.53078	0.28320
H	2.50359	-2.84358	1.20032
H	2.52872	-3.02685	-0.56404
H	4.06052	-2.87917	0.33510
C	-5.12869	-0.96349	0.08479
H	-5.84824	-0.37702	-0.50548
H	-5.14294	-1.99444	-0.29069
H	-5.50121	-0.97835	1.12049

Compound 34b imine

Electronic energy: -597.137314400 Hartree

C	1.46157	-0.65058	-0.61972
C	2.16816	-1.66904	0.02972
C	3.26823	-1.32586	0.81285
C	3.62757	0.01539	0.92017
C	2.87349	0.97564	0.23345
H	1.86115	-2.70887	-0.08045
H	3.84051	-2.09589	1.33248
H	4.48331	0.31829	1.52338
N	1.81356	0.63943	-0.51866
C	0.24646	-0.95674	-1.47060
H	0.21913	-2.03523	-1.67834
H	0.35562	-0.43598	-2.43146
C	-1.05183	-0.53015	-0.81601

C	-1.55662	-1.22460	0.29388
C	-1.78146	0.56365	-1.29003
C	-2.74599	-0.83414	0.90587
H	-1.01212	-2.08628	0.68535
C	-2.97493	0.95538	-0.67442
H	-1.41652	1.12061	-2.15496
C	-3.47935	0.26644	0.43363
H	-3.11524	-1.39662	1.76623
H	-3.52308	1.81338	-1.06893
C	3.21531	2.43772	0.30062
H	2.36540	3.01135	0.69616
H	4.08759	2.61770	0.93915
H	3.42999	2.82686	-0.70465
C	-4.76426	0.68020	1.10104
H	-5.50368	-0.13369	1.07797
H	-4.59843	0.93405	2.15826
H	-5.20664	1.55444	0.60769

Compound 34b enamine

Electronic energy: -597.104663308 Hartree

C	1.51502	-0.83922	0.40440
C	2.48065	-1.58076	-0.21508
C	3.61297	-0.89752	-0.77408
C	3.72725	0.45636	-0.69580
C	2.71140	1.26544	-0.04803
H	0.89429	1.03883	0.91248
H	2.38523	-2.66228	-0.27262
H	4.39313	-1.47726	-1.27063
H	4.58573	0.97415	-1.12258
N	1.64606	0.52086	0.47466
C	-1.01015	-0.82477	0.57725
C	-1.46516	-1.06029	-0.72915
C	-1.78835	-0.01525	1.40940
C	-2.65809	-0.50304	-1.18201
H	-0.87740	-1.69057	-1.39944
C	-2.98574	0.54571	0.95139
H	-1.46334	0.17818	2.43378
C	-3.44235	0.31329	-0.34961
H	-2.99059	-0.70652	-2.20212
H	-3.57364	1.17288	1.62427
C	2.75587	2.62376	0.06072
H	3.59769	3.17091	-0.35669
H	1.96670	3.18674	0.55807
C	0.29285	-1.42925	1.06039
H	0.37435	-1.28981	2.14940
H	0.30472	-2.51031	0.87313
C	-4.73102	0.90896	-0.85050
H	-5.43985	0.12330	-1.15023
H	-5.21204	1.52485	-0.08091
H	-4.55819	1.54040	-1.73410

Compound 34b radical

Electronic energy: -597.104663308 Hartree

C	1.51502	-0.83922	0.40440
C	2.48065	-1.58076	-0.21508
C	3.61297	-0.89752	-0.77408
C	3.72725	0.45636	-0.69580
C	2.71140	1.26544	-0.04803
H	0.89429	1.03883	0.91248
H	2.38523	-2.66228	-0.27262
H	4.39313	-1.47726	-1.27063
H	4.58573	0.97415	-1.12258
N	1.64606	0.52086	0.47466
C	-1.01015	-0.82477	0.57725
C	-1.46516	-1.06029	-0.72915
C	-1.78835	-0.01525	1.40940
C	-2.65809	-0.50304	-1.18201
H	-0.87740	-1.69057	-1.39944
C	-2.98574	0.54571	0.95139
H	-1.46334	0.17818	2.43378
C	-3.44235	0.31329	-0.34961
H	-2.99059	-0.70652	-2.20212
H	-3.57364	1.17288	1.62427
C	2.75587	2.62376	0.06072
H	3.59769	3.17091	-0.35669
H	1.96670	3.18674	0.55807
C	0.29285	-1.42925	1.06039
H	0.37435	-1.28981	2.14940
H	0.30472	-2.51031	0.87313
C	-4.73102	0.90896	-0.85050
H	-5.43985	0.12330	-1.15023
H	-5.21204	1.52485	-0.08091
H	-4.55819	1.54040	-1.73410

Compound 35 imine

Electronic energy: -671.134281045 Hartree

C	-2.78014	1.20715	0.31357
C	-1.51529	-0.69180	-0.02941
C	-2.58560	-1.38040	-0.60929
C	-3.79826	-0.71025	-0.74247
C	-3.89895	0.59698	-0.27148
H	-2.45659	-2.40748	-0.94527
H	-4.65655	-1.20072	-1.20296
H	-4.83687	1.14590	-0.35488
C	-2.83882	2.60757	0.85325
H	-2.60432	2.61211	1.92687
H	-2.09247	3.24079	0.35400
H	-3.83016	3.05121	0.70949
C	-0.20762	-1.41483	0.16512
O	-0.23282	-2.62067	0.40088

C	1.08038	-0.68083	0.06931
C	2.21618	-1.23263	0.68766
C	1.22443	0.49962	-0.67390
C	3.45291	-0.61023	0.57944
H	2.11101	-2.15392	1.26011
C	2.47420	1.10575	-0.79748
H	0.36309	0.94047	-1.17172
C	3.60597	0.57056	-0.16884
H	4.32009	-1.04530	1.07956
H	2.57075	2.01523	-1.39256
N	-1.60442	0.56890	0.41544
C	4.95094	1.23143	-0.28438
H	5.32038	1.54052	0.70432
H	5.69436	0.53712	-0.70172
H	4.90884	2.11862	-0.92698

Compound 35 enamine
Electronic energy: -671.104269024 Hartree

C	3.81288	-0.03203	0.14491
C	1.37014	-0.09390	-0.06178
C	1.31002	1.20502	-0.48753
C	2.53913	1.93661	-0.60580
C	3.72730	1.34846	-0.30056
H	0.36128	1.65540	-0.76080
H	2.51425	2.97115	-0.94903
H	4.66188	1.90152	-0.39145
C	0.21106	-1.02292	0.02262
O	0.43701	-2.23429	0.07951
C	-1.17973	-0.50484	0.02602
C	-2.17376	-1.25510	-0.62632
C	-1.55556	0.65259	0.72284
C	-3.49788	-0.83604	-0.61025
H	-1.89116	-2.16383	-1.15753
C	-2.89185	1.05267	0.75265
H	-0.81161	1.22511	1.27542
C	-3.88282	0.32631	0.08116
H	-4.25208	-1.42097	-1.13973
H	-3.16830	1.94572	1.31508
H	2.53585	-1.65514	0.47754
C	4.98264	-0.65647	0.45028
H	5.01064	-1.69472	0.77835
H	5.92040	-0.11282	0.36734
N	2.57421	-0.67155	0.23293
C	-5.32077	0.76410	0.09723
H	-5.67634	0.97441	-0.92192
H	-5.96691	-0.02597	0.50606
H	-5.45834	1.66850	0.70136

Compound 35 radical
Electronic energy: -671.104269024 Hartree

C	3.81288	-0.03203	0.14491
C	1.37014	-0.09390	-0.06178
C	1.31002	1.20502	-0.48753
C	2.53913	1.93661	-0.60580
C	3.72730	1.34846	-0.30056
H	0.36128	1.65540	-0.76080
H	2.51425	2.97115	-0.94903
H	4.66188	1.90152	-0.39145
C	0.21106	-1.02292	0.02262
O	0.43701	-2.23429	0.07951
C	-1.17973	-0.50484	0.02602
C	-2.17376	-1.25510	-0.62632
C	-1.55556	0.65259	0.72284
C	-3.49788	-0.83604	-0.61025
H	-1.89116	-2.16383	-1.15753
C	-2.89185	1.05267	0.75265
H	-0.81161	1.22511	1.27542
C	-3.88282	0.32631	0.08116
H	-4.25208	-1.42097	-1.13973
H	-3.16830	1.94572	1.31508
H	2.53585	-1.65514	0.47754
C	4.98264	-0.65647	0.45028
H	5.01064	-1.69472	0.77835
H	5.92040	-0.11282	0.36734
N	2.57421	-0.67155	0.23293
C	-5.32077	0.76410	0.09723
H	-5.67634	0.97441	-0.92192
H	-5.96691	-0.02597	0.50606
H	-5.45834	1.66850	0.70136

IX. References

(S1) P. Höfer, A. Grupp, H. Nebenführ. M. Mehring, *Chem. Phys. Lett*. 1986, **132**, 279.

(S2) E. R. Davies, New pulse ENDOR technique, *Phys. Lett. A* 1974, **47**, 1-2.

(S3) S. Stoll and A. Schweiger, *J. Magn. Reson.* 2006, **178**, 42.

(S4) A. Pöppl, M. Gutjahr, M. Hartmann, W. Böhlmann, R. Böttcher, *J. Phys. Chem. B* 1998, **102**, 7752-7763.

(S5) H. Thomann, M. Bernardo, *Methods Enzymol.* 1993, **227**, 118.

(S6) P. Metrangolo, G. Resnati, in Halogen Bonding I, Series Vol. 358, Ed. 1, Springer International Publishing Switzerland, 2015.

(S7) J. De Houwer, K. Abbaspour Tehrani, B. U. W. Maes, *Angew. Chem. Int. Ed.* 2012, **51**, 2745-2748.

(S8) S. Rezazadeh, B. Akhlaghinia, N. Razavi, *Aust. J. Chem.* 2015, **68**, 145-155.

(S9) T. Zarganes-Tzitzikas, C. G. Neochoritis, J. Stephanidou-Stephanatou, C. A. Tsoleridis, *J. Org. Chem.* 2011, **76**, 1468-1471.

(S10) P. Lassalas, F. Marsais, C. Hoarau, *Synlett* 2013, **24**, 2233-2240.

(S11) M. J. Frisch, G. W. Trucks, H. B. Schlegel, G. E. Scuseria, M. A. Robb, J. R. Cheeseman, G. Scalmani, V. Barone, B. Mennucci, G. A. Petersson, H. Nakatsuji, M. Caricato, X. Li, H. P. Hratchian, A. F. Izmaylov, J. Bloino, G. Zheng, J. L. Sonnenberg, M. Hada, M. Ehara, K. Toyota, R. Fukuda, J. Hasegawa, M. Ishida, T. Nakajima, Y. Honda, O. Kitao, H. Nakai, T. Vreven, J. A. Montgomery Jr., J. E. Peralta, F. Ogliaro, M. J. Bearpark, J. Heyd, E. N. Brothers, K. N. Kudin, V. N. Staroverov, R. Kobayashi, J. Normand, K. Raghavachari, A. P. Rendell, J. C. Burant, S. S. Iyengar, J. Tomasi, M. Cossi, N. Rega, N. J. Millam, M. Klene, J. E. Knox, J. B. Cross, V. Bakken, C. Adamo, J. Jaramillo, R. Gomperts, R. E. Stratmann, O. Yazyev, A. J. Austin, R. Cammi, C. Pomelli, J. W. Ochterski, R. L. Martin, K. Morokuma, V. G. Zakrzewski, G. A. Voth, P. Salvador, J. J. Dannenberg, S. Dapprich, A. D. Daniels, Ö. Farkas, J. B. Foresman, J. V. Ortiz, J. Cioslowski, D. J. Fox, Gaussian, Inc., Wallingford, CT, USA, **2009**.

(S12) V. Van Speybroeck, G. B. Marin, M. Waroquier, *ChemPhysChem* 2006, **7**, 2205-2214.

(S13) H. Ishibashi, T. Sato, M. Ikeda, *Synthesis* 2002, **6**, 695-713.

X. NMR spectra

2-Benzylbenzoxazole (**22**)

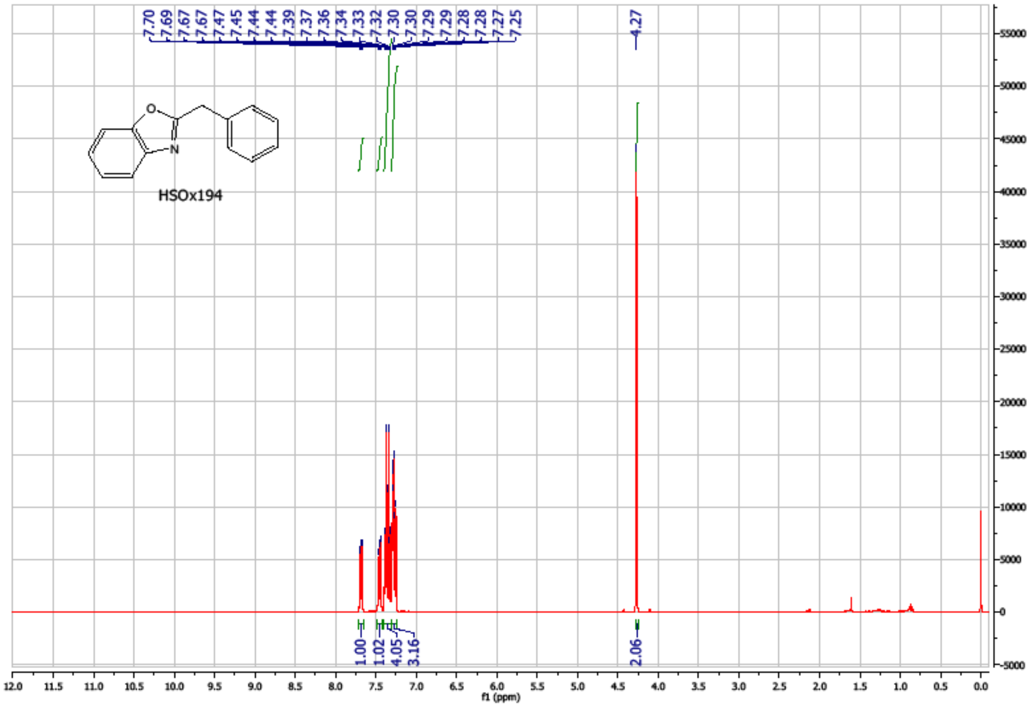

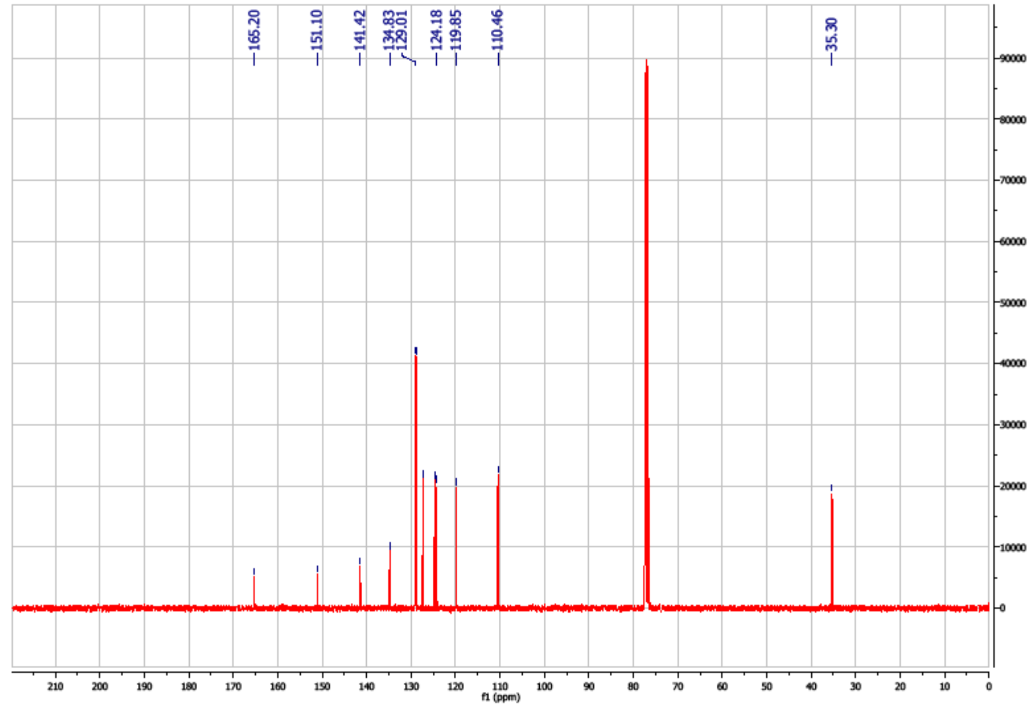

2-Benzylbenzothiazole (**24**)

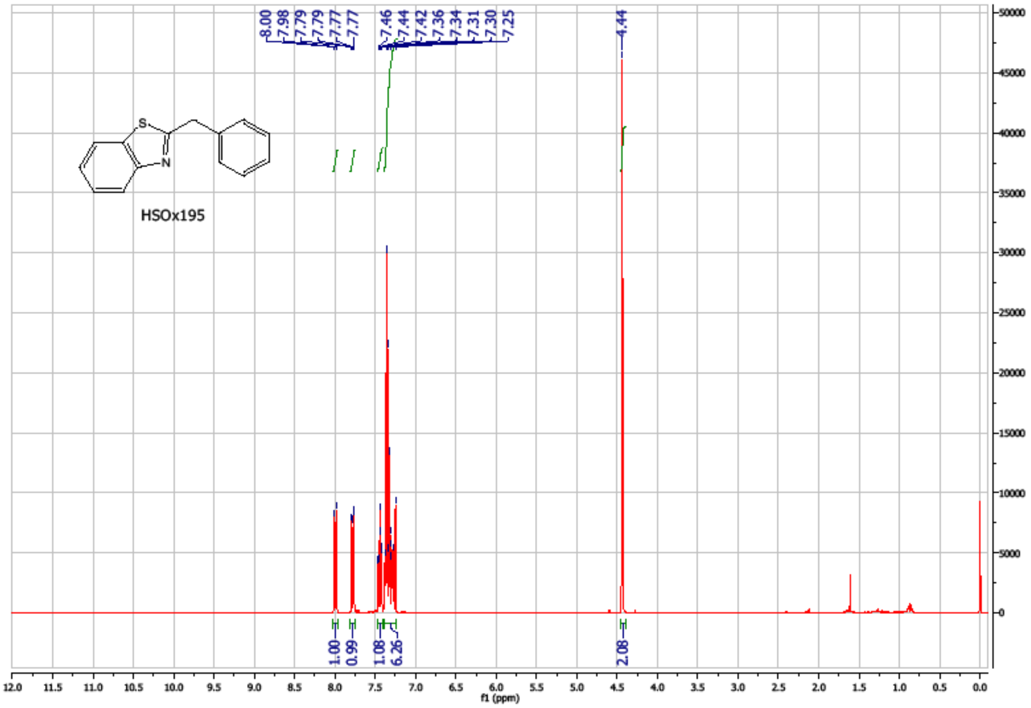

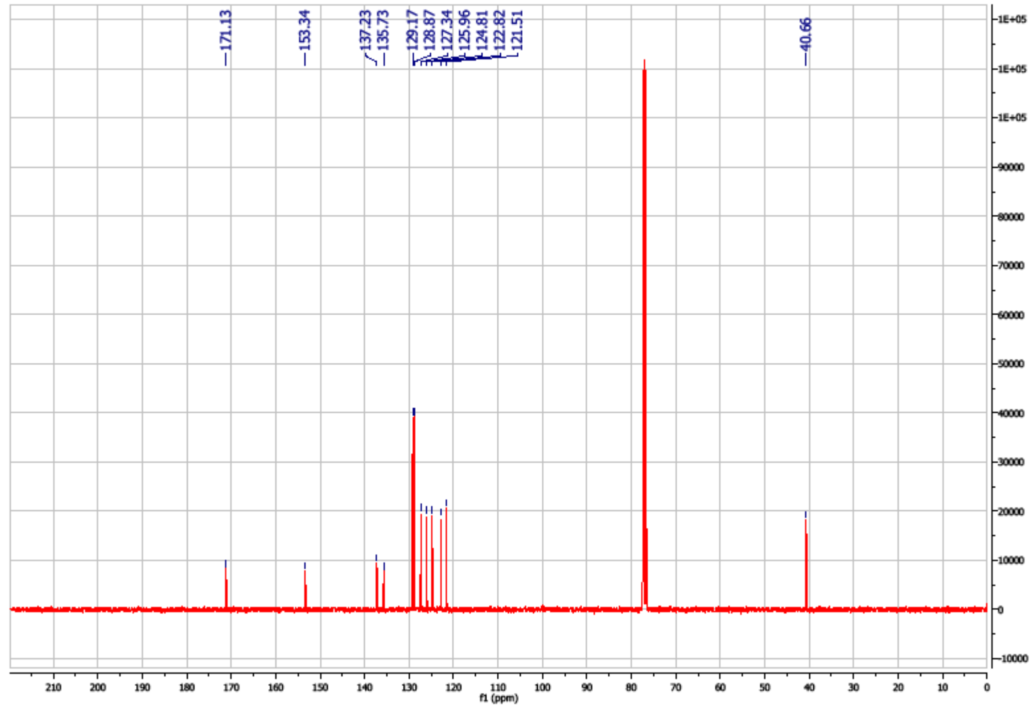

2-benzyl-1-methyl-1*H*-imidazole (**18**)

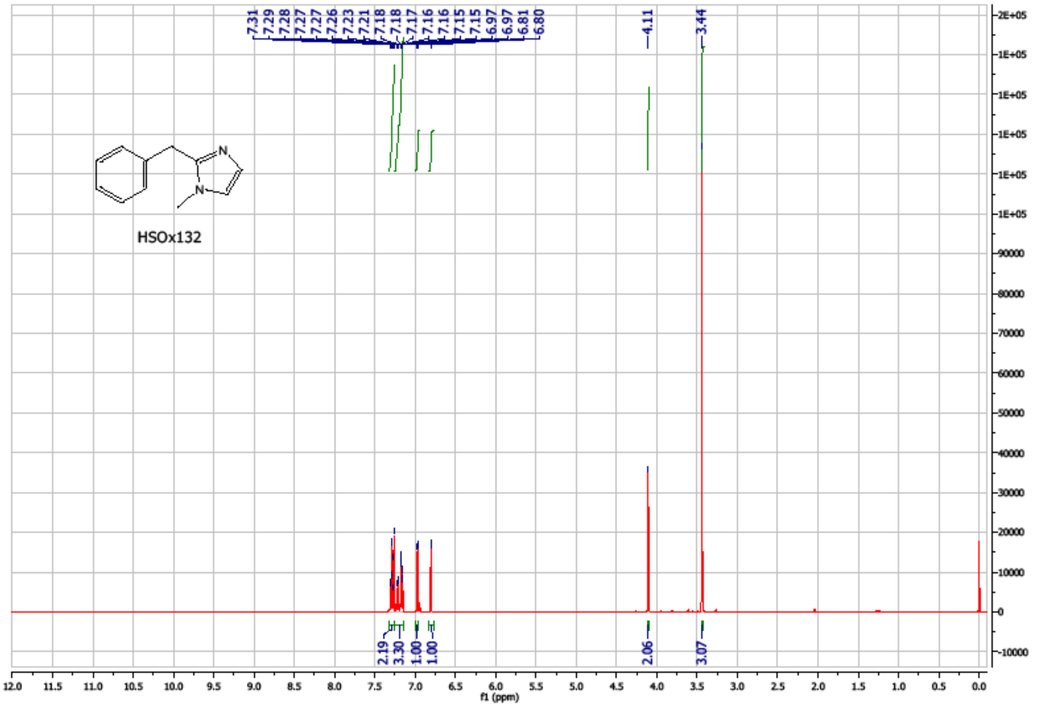

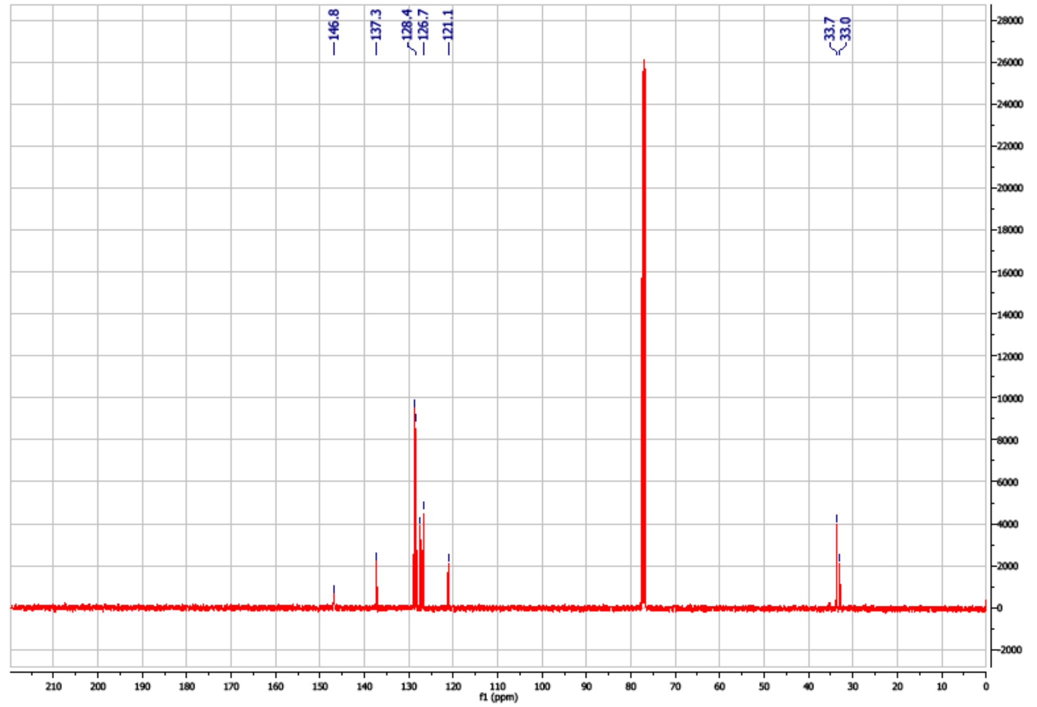

2-{[2-(prop-2-en-1-yl)phenyl]methyl}pyridine **(26)**

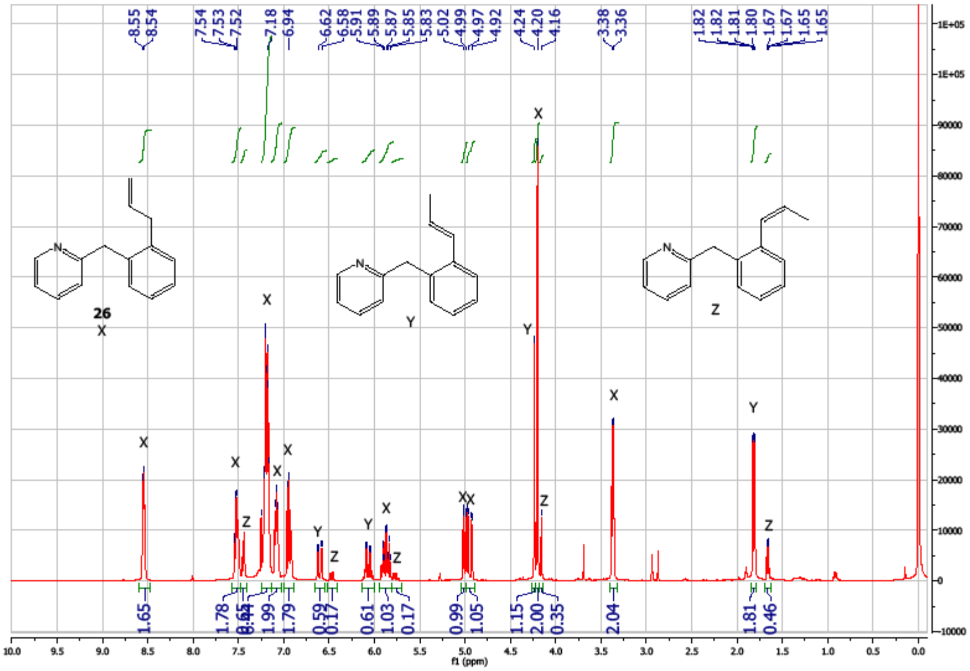

6-Formylnicotinonitrile (**13**)

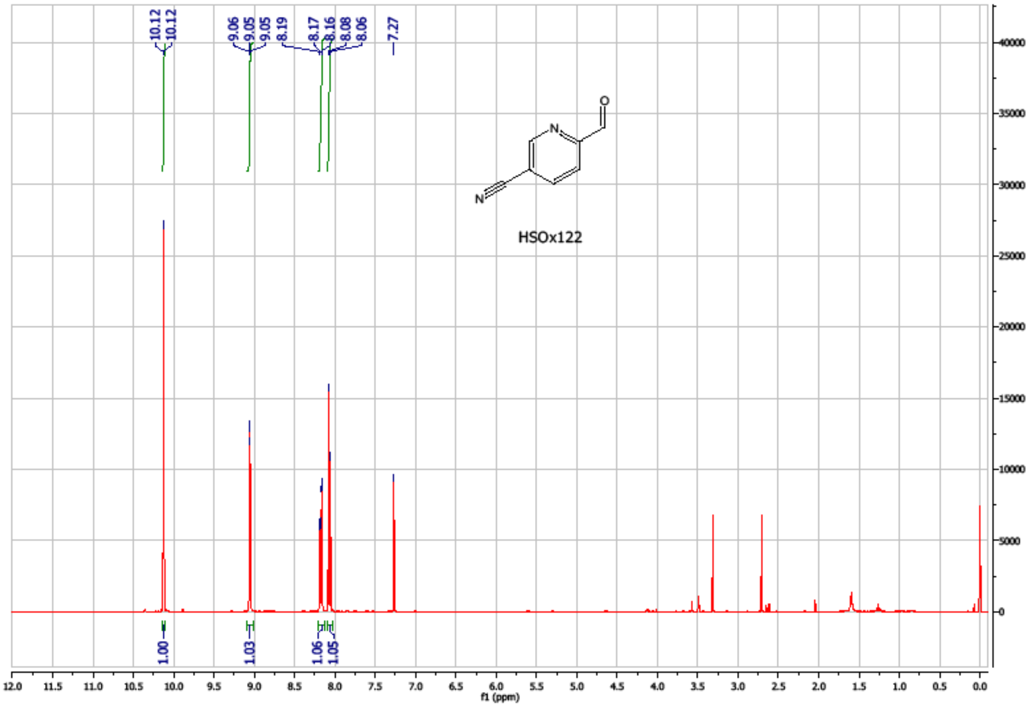

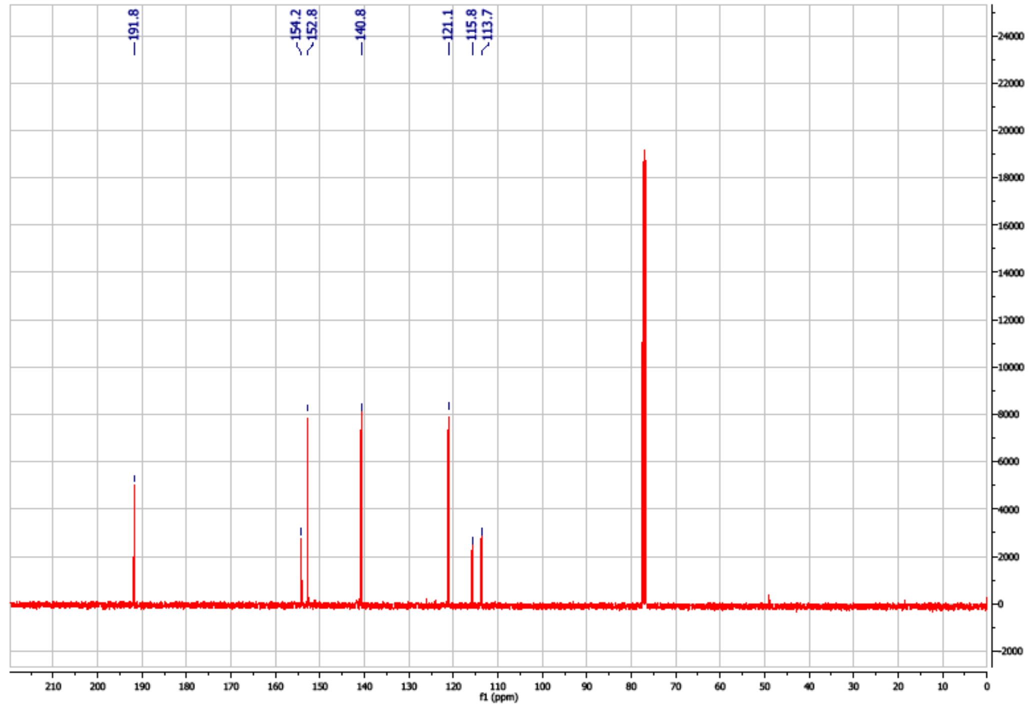

(1-Methyl-1*H*-imidazol-2-yl)(phenyl)methanone (**19**)

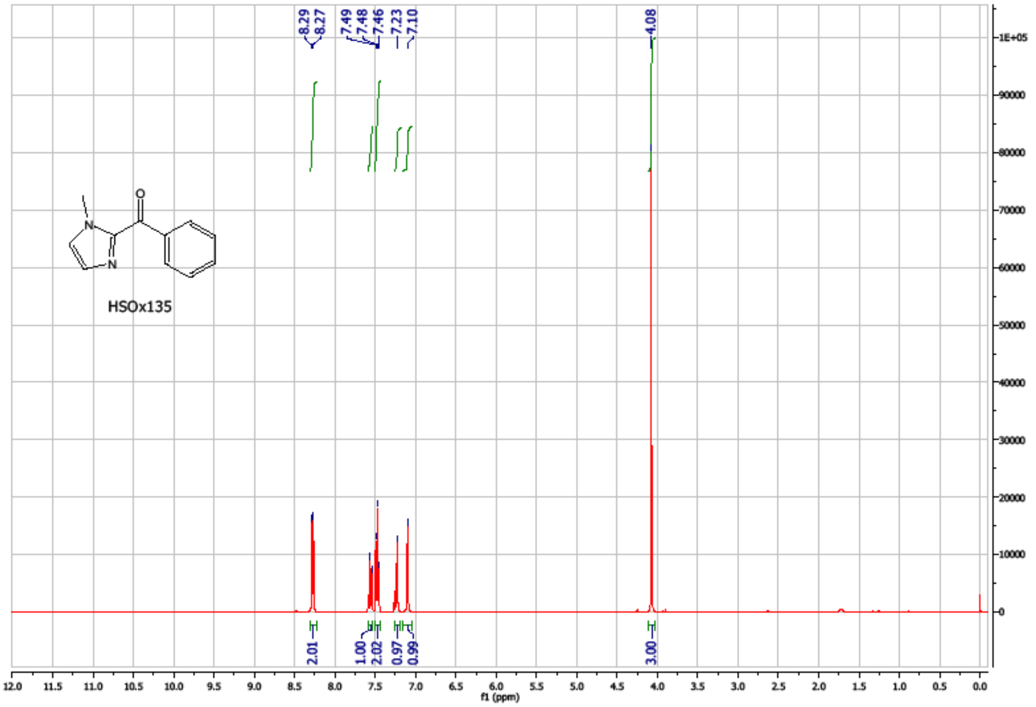

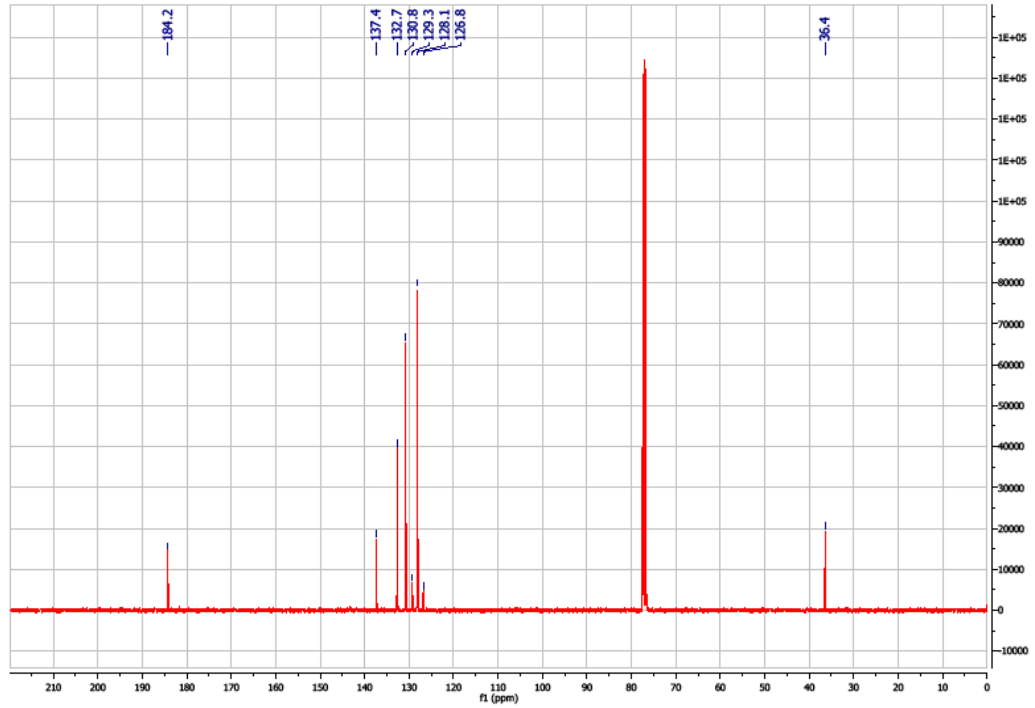

Benzothiazol-2-yl(phenyl)methanone (**25**)

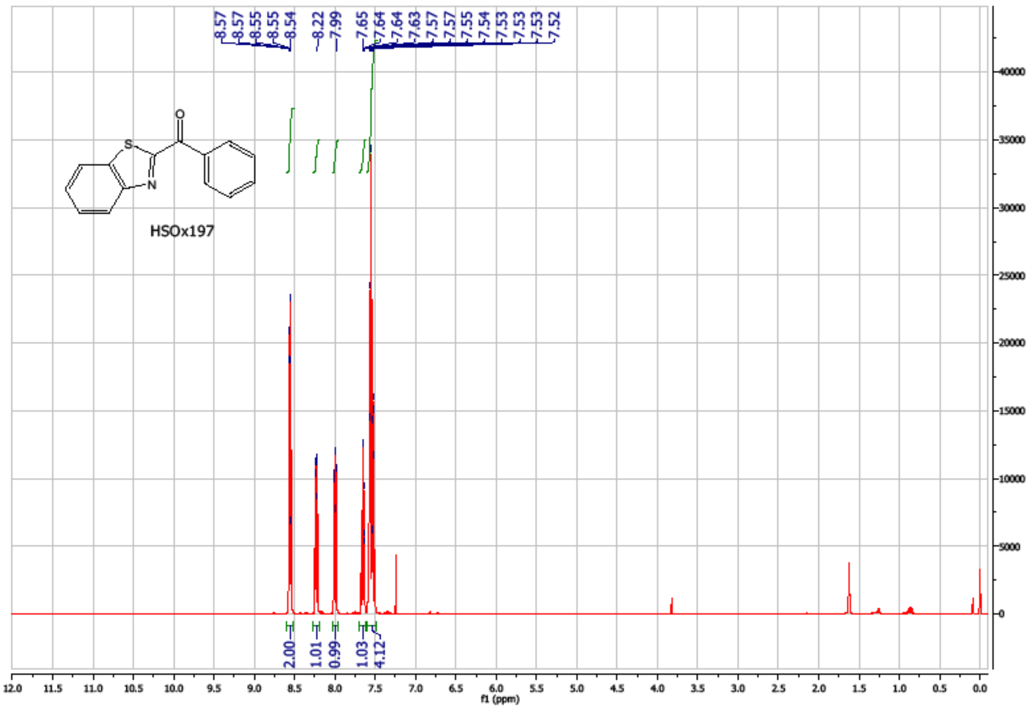

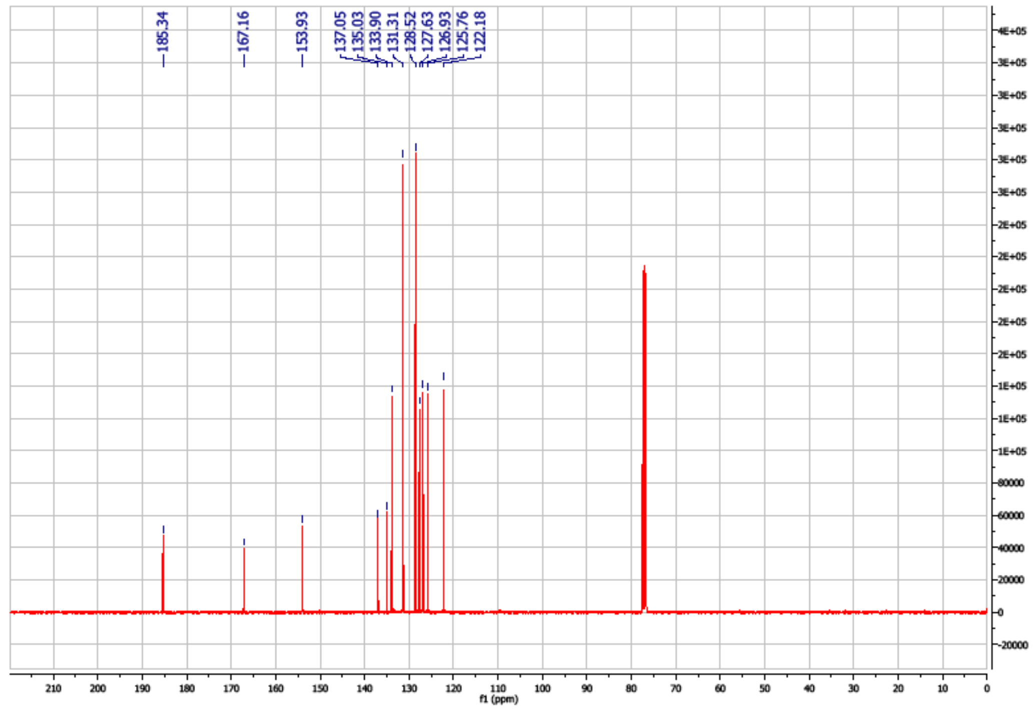

Benzoxazol-2-yl(phenyl)methanone (**23**)

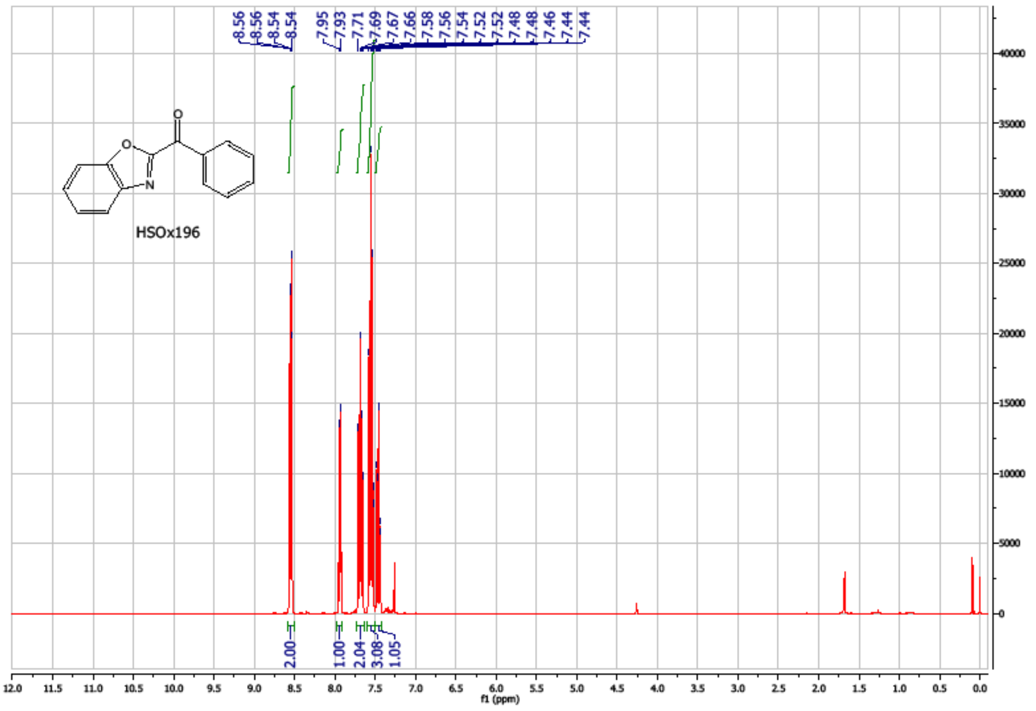

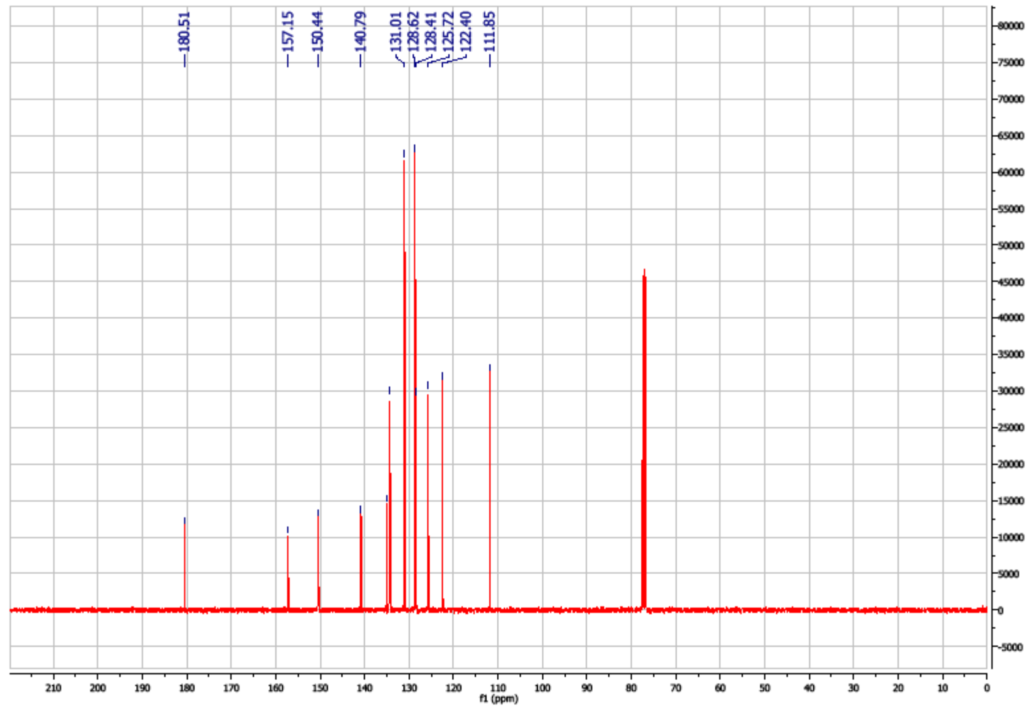

Methyl oxo(pyridin-2-yl)acetate (**15**)

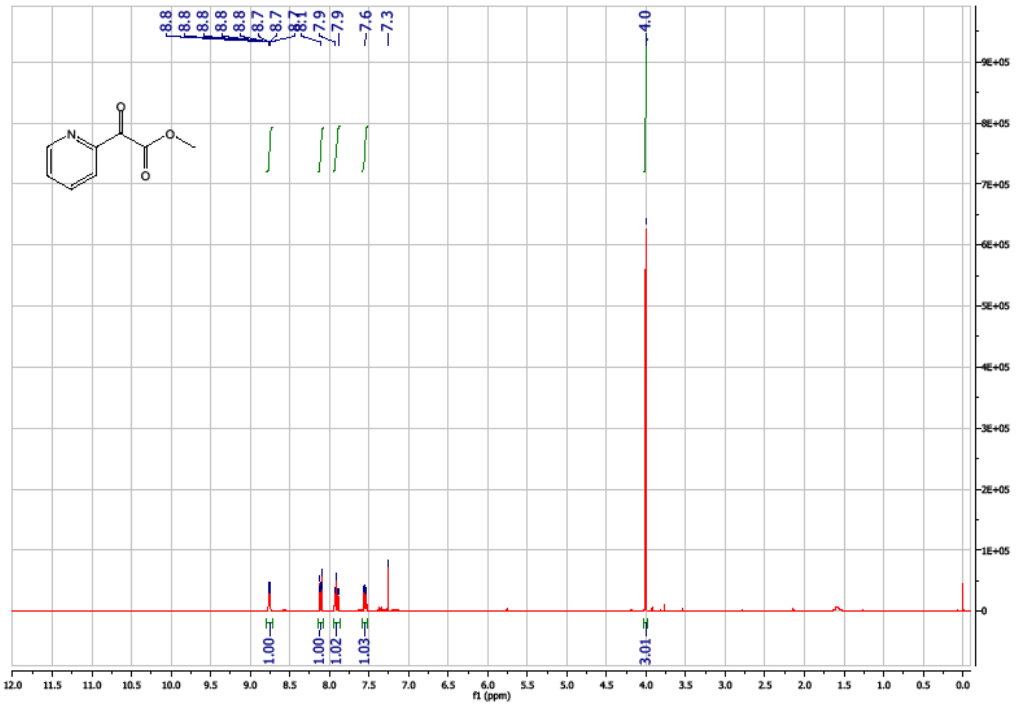

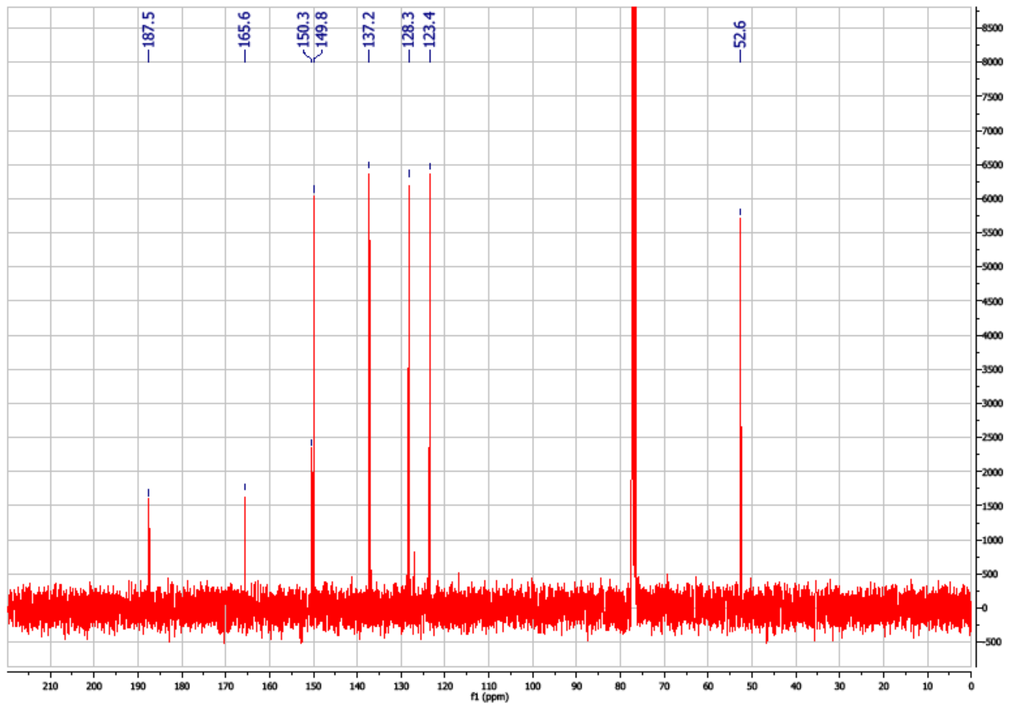

[2-(prop-2-en-1-yl)phenyl](pyridin-2-yl)methanone (**27**)

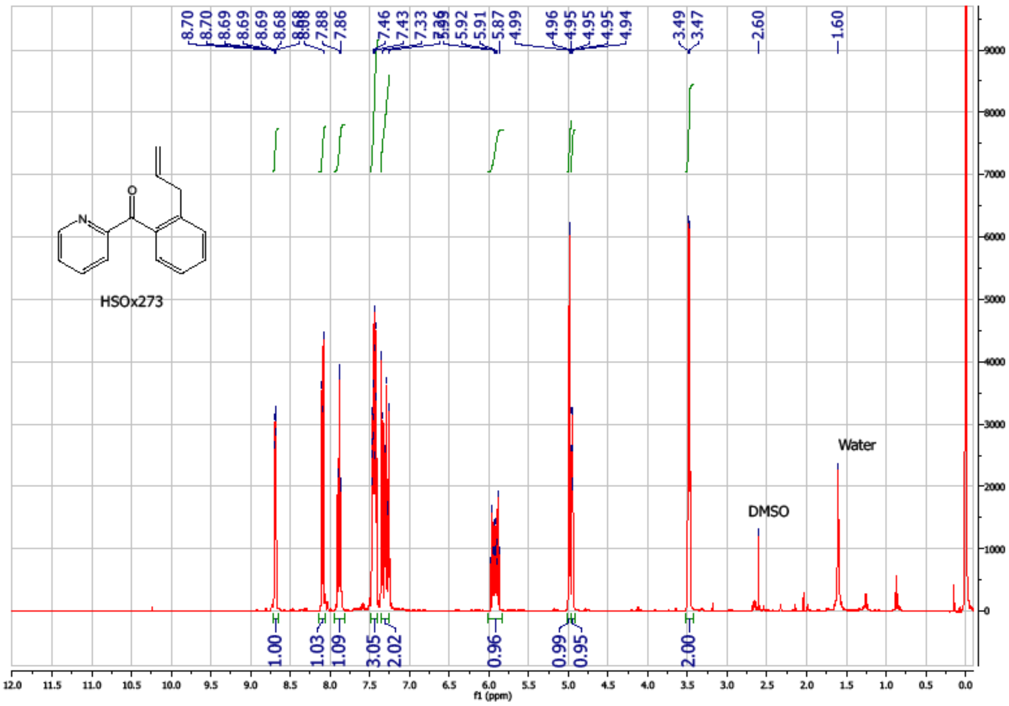

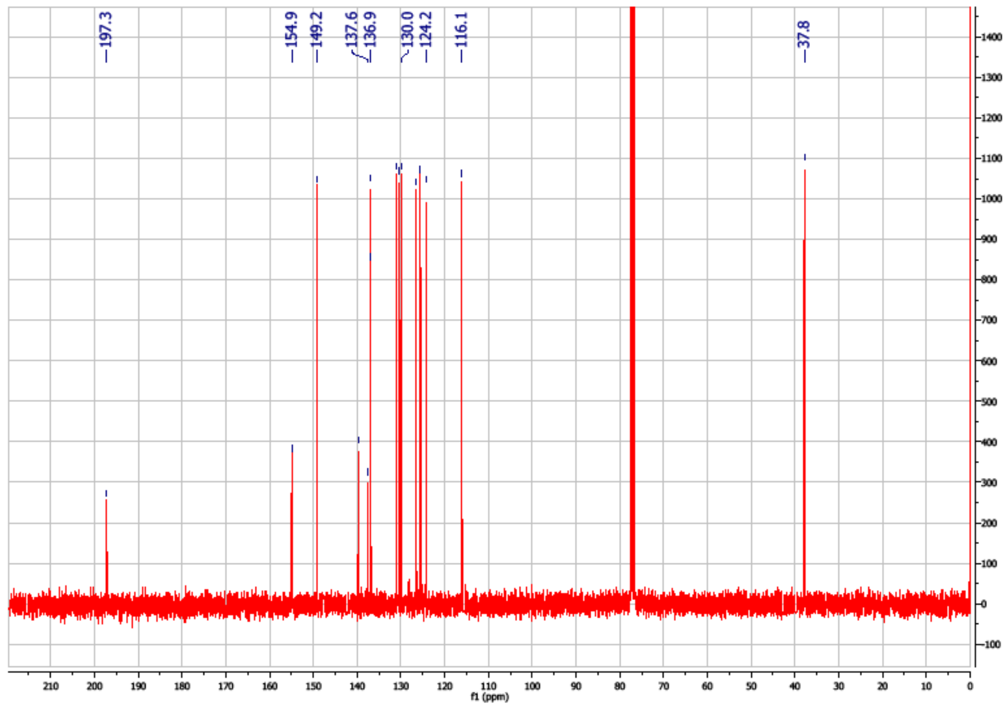

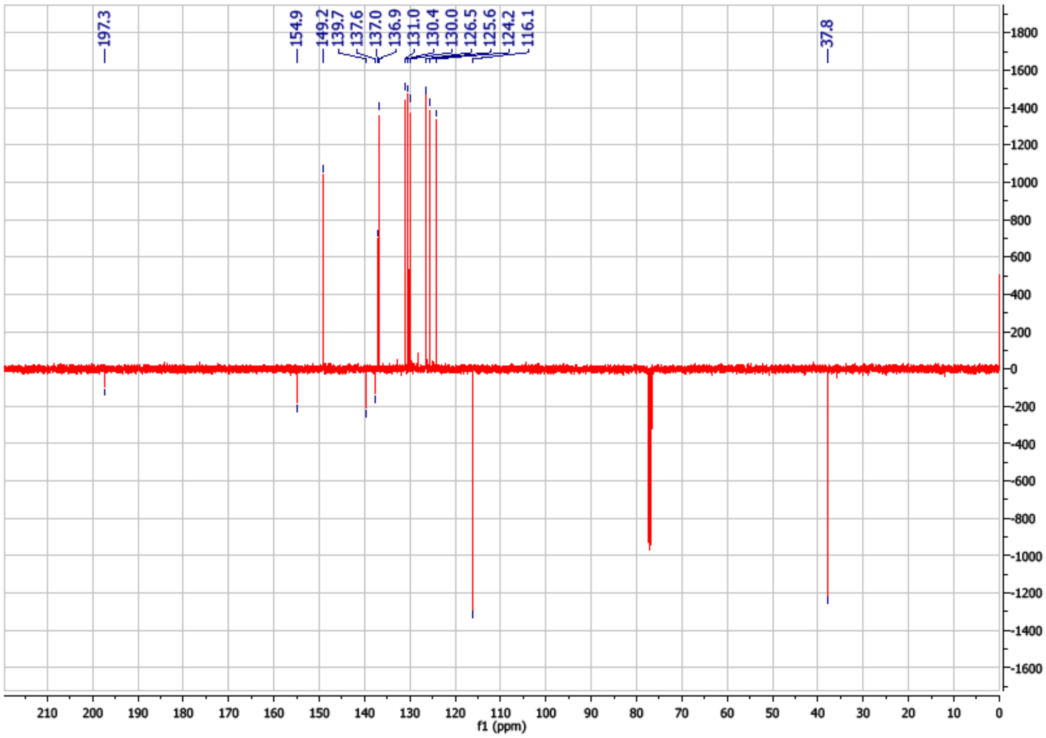

197.3
154.9
149.2
139.7
137.6
137.0
136.9
131.0
130.4
130.0
126.5
125.6
124.2
116.1
37.8
f1 (ppm)

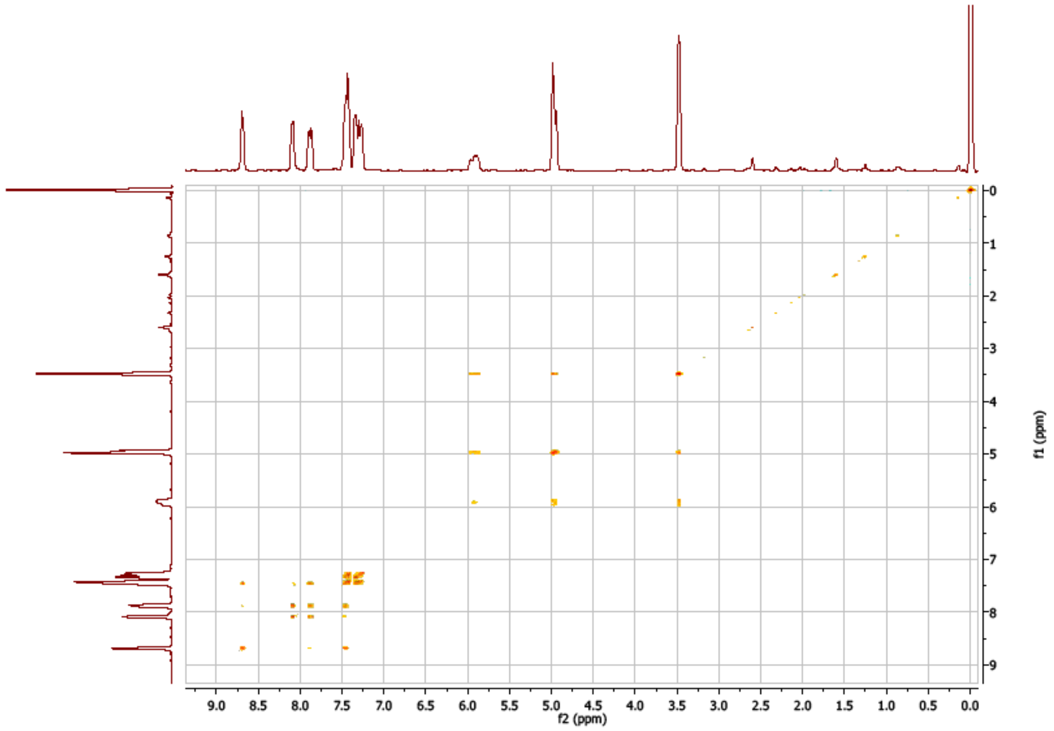

f2 (ppm)
f1 (ppm)
